# Cooperative C–H Bond Activation by a Low-Spin d^6^ Iron–Aluminum Complex

**DOI:** 10.1021/jacs.2c02662

**Published:** 2022-05-05

**Authors:** Nikolaus Gorgas, Andrew J. P. White, Mark R. Crimmin

**Affiliations:** Department of Chemistry, Imperial College London, White City, London W12 0BZ, United Kingdom

## Abstract

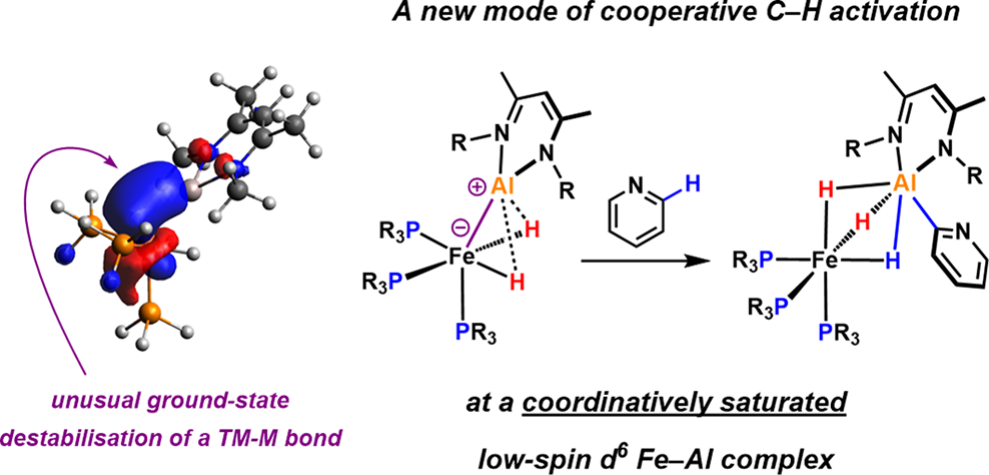

The reactions of transition metal complexes underpin numerous synthetic processes and catalytic transformations. Typically, this reactivity involves the participation of empty and filled molecular orbitals centered on the transition metal. Kinetically stabilized species, such as octahedral low-spin *d*^6^ transition metal complexes, are not expected to participate directly in these reactions. However, novel approaches that exploit metal–ligand cooperativity offer an opportunity to challenge these preconceptions. Here, we show that inclusion of an aluminum-based ligand into the coordination sphere of neutral low-spin *d*^6^ iron complex leads to unexpected reactivity. Complexes featuring an unsupported Fe–Al bond are capable of the intermolecular C–H bond activation of pyridines. Mechanistic analysis suggests that C–H activation proceeds through a reductive deprotonation in which the two metal centers (Fe and Al) act like a frustrated Lewis pair. The key to this behavior is a ground state destabilization of the *d*^6^ iron complex, brought about by the inclusion of the electropositive aluminum-based ligand. These findings have immediate implications for the design of reagents and catalysts based on first-row transition metals.

## Introduction

**Figure 1:**
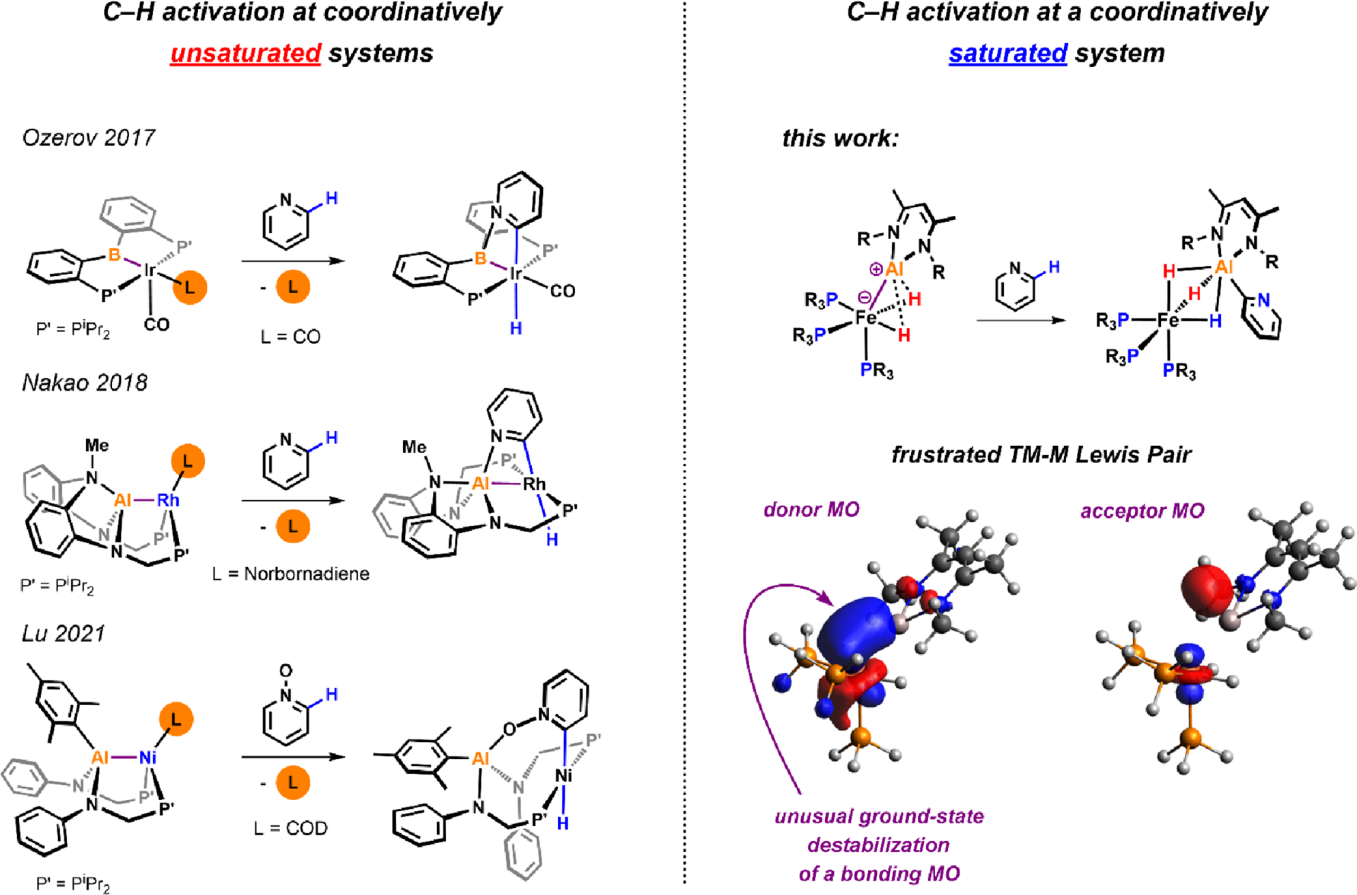
Well-defined bimetallic systems for the selective ortho C–H activation of pyridines. Current systems that require ligand dissociation prior to bond activation *vs* a novel pathway occurring at a coordinatively saturated iron aluminylene system.

The concept of metal–ligand cooperativity has greatly enriched the chemistry and catalytic applications of transition metal complexes.^1,2^ Cooperative strategies that take advantage of the Lewis acidic nature of a transition metal center in combination with a Lewis basic ligand have become established features, frequently employed in the design of novel catalytic systems. Equally attractive but less common are transition metal complexes that bear a Lewis acidic functionality in the ligand.^3−5^ These new design principles offer an opportunity to overturn existing paradigms in transition metal chemistry. This is particularly important for applications of inexpensive and sustainable first-row transition metals (*e.g.*, Fe).^6−9^ Despite the exciting opportunities in this field, many of the emerging reactions that involve metal–ligand cooperativity still proceed through established mechanisms, such as oxidative addition. For example, transition metal complexes bearing Lewis acidic ligands (based on B or Al) can activate the ortho C–H bond of pyridine substrates (Figure 1).^10−13^ C–H activation is believed to take place by an oxidative addition mechanism at the transition metal center, leading to products in which the pyridyl group is directly bonded to this metal.^13−15^ The main group ligand plays a role in substrate coordination and determining the ortho selectivity but itself does not lead to new types of reactivities.

In this paper, we report the synthesis and characterization of bimetallic complexes in which an unsupported^16^ aluminum-based ligand is bound to an iron(II) dihydride fragment. These complexes are neutral low-spin *d*^6^ species based on an octahedral parent geometry and as such are expected to be chemically inert.^17^ Textbook examples of such compounds with more classical ligand systems possess a stable 18-electron configuration and a large highest occupied molecular orbital (HOMO) (*t*_2g_)—lowest unoccupied molecular orbital (LUMO) (e_g_) gap, limiting direct reactivity at the metal site.^18^ Inclusion of the aluminum-based ligand results in a significant distortion of the geometry away from octahedral due to favorable, but weak, interactions in the secondary coordination sphere. This distortion leads to an unusual ground state destabilization and raises the energy of the HOMO of the iron center. This effect exposes an entirely new type of reactivity of the neutral low-spin *d*^6^ center. These complexes were found to selectively break the ortho C–H bond in pyridine. Mechanistic studies reveal that the two metal centers act as a frustrated Fe–Al Lewis pair (FLP)^19−23^ leading to a deprotonation rather than oxidative addition pathway.^24^

The findings both compliment and expand upon the known nucleophilicity of anionic analogues such as [Fe(η^5^-C_5_H_5_)(CO)_2_]^−^. More broadly, these results suggest that the shape and electronic structure of transition metal centers can be modulated through incorporation of Lewis acidic ligands, leading to perturbation of the electronic structure and exposing new types of reactivities.

## Results and Discussion

### Synthesis and Characterization

**Figure 2:**
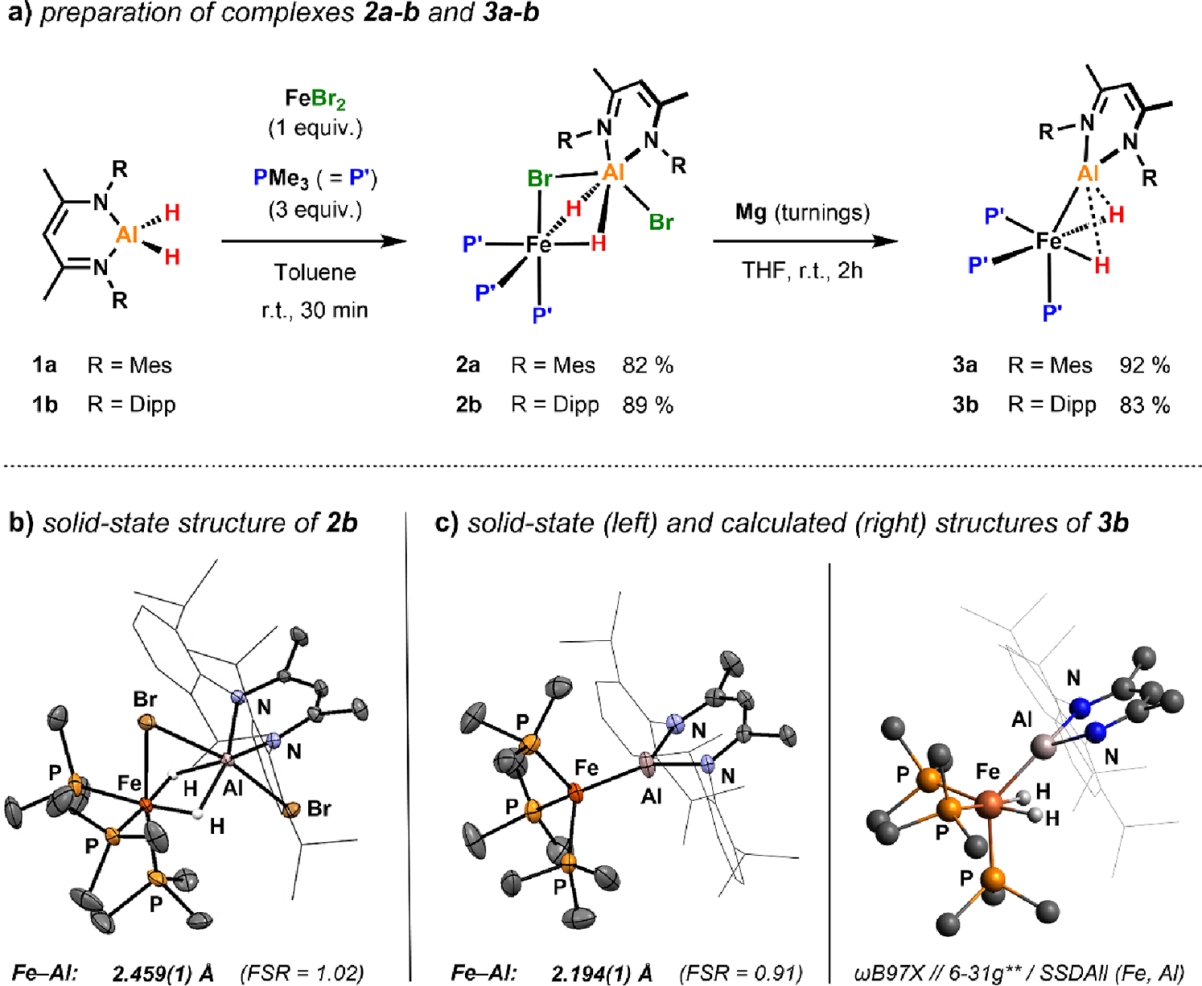
(a) Preparation of complexes **2a-b** and **3a-b**. (b) X-ray structure of **2b**. (c) X-ray and calculated structure of **3b**.

Complexes **2a** and **2b** could be readily prepared by reacting FeBr_2_, PMe_3_, and respective β-diketiminate aluminum hydrides **1a** (R = Mesityl or Mes) and **1b** (R = 2,6-diisopropylphenyl or Dipp) in toluene or benzene and were isolated in 80–90% yield (Figure 2). Both complexes exhibit a mutually coupled spin system comprised of one triplet and one doublet resonance in the ^31^P{^1^H} NMR spectra with an integration ratio of 1:2. These resonances are consistent with the magnetic non-equivalence of the axial and equatorial phosphine ligands. The most characteristic features in the ^1^H NMR spectra are broad signals at δ_H_ = −13.08 (**2a**) or −13.20 (**2b**) ppm assigned to the bridging hydrides which are coupled to the quadrupolar *I* = 5/2 ^27^Al nucleus.

Reduction of these precursors using magnesium turnings in tetrahydrofuran affords complexes **3a** and **3b** in almost quantitative NMR yield. Both **3a** and **3b** can be isolated as dark red/orange crystalline solids. In contrast to complexes **2a-b**, **3a-b** both show only one singlet resonance in the ^31^P{^1^H} and a well resolved quartet hydride resonance in the ^1^H NMR spectra. The data are consistent with a highly symmetric structure in solution—an observation that could suggest fast ligand exchange on the NMR timescale.

### Structure and Bonding

Single crystals suitable for X-ray diffraction could be obtained for all four compounds. Data for **2b** and **3b** are depicted in Figure 2. The position of the hydride ligands in **3b** could not be refined, but their presence is evident from the corresponding ^1^H NMR spectrum. When comparing these structures, it becomes apparent that drastic changes in the Fe···Al intermetallic distances occur upon reduction. For example, a decrease in the Fe···Al distance from 2.459(1) Å in **2b** to 2.194(1) Å in **3b** becomes visible in the solid-state structures. Although the Fe···Al separation of **2b** almost matches the sum of their covalent radii^25^ [formal shortness ratio^26^ (FSR) = 1.02], it is clearly below this value in **3b** (FSR = 0.91). Such short Fe···Al bond lengths appear to be diagnostic for an aluminylene^27^ metalloligand covalently bound to iron.^28^

**Figure 3:**
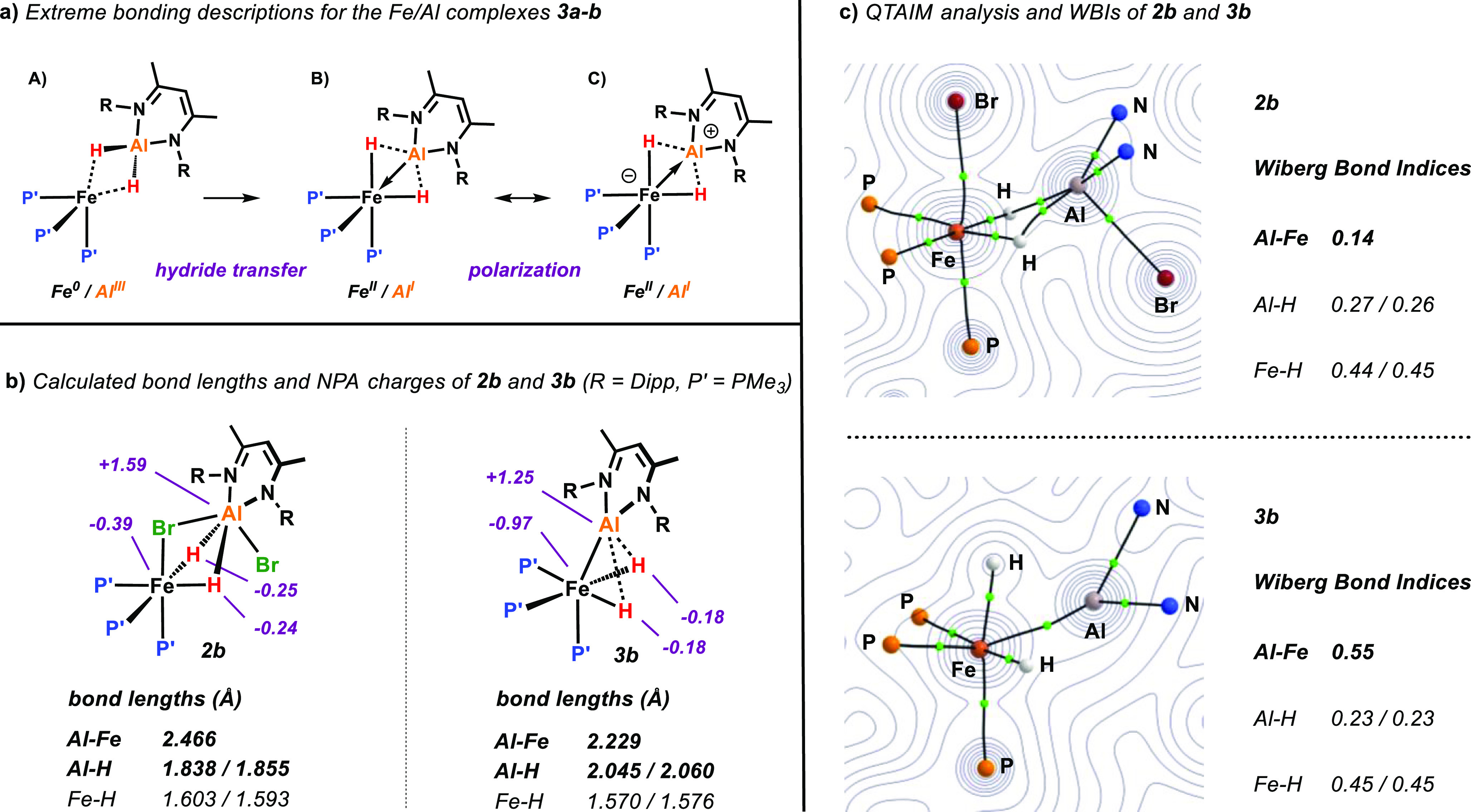
Analysis of the bonding in **3a-b**. (a) Extreme bonding descriptions. (b) Calculated Fe···Al distances and NPA charges of **2a** and **3a** [ωB97X//6-31g** (H,C,N,P)/SDDAll (Fe,Al)]. (c) QTAIM analysis and WBIs of **2a** and **3a**.

Based on the experimental data, it is evident that **2a-b** and **3a-b** are low-spin and diamagnetic. These species can thus be assigned as 18-valence electron complexes. Although **2a-b** can be confidently described as σ-alane^29^ complexes [see the quantum theory of atoms in molecules (QTAIM) analysis below], three extreme bonding scenarios^16^ may be considered for bimetallic species **3a-b** (Figure 3a): (**A**) a four-electron η^2^:η^2^-coordination of H–Al–H to the 14-electron fragment [Fe(PMe_3_)_3_], (**B**) two-electron coordination of an aluminylene metalloligand to a 16-electron Fe(II) dihydride fragment, and (**C**) strong polarization of the TM–M bond resulting in a cationic Al metalloligand and an anionic iron dihydride fragment due to the electronegativity difference between the two metals (Δχ_P_ = 0.22).^30,31^ According to the computational results presented below, an intermediate between structures B and C in Figure 3a appears as the most appropriate bonding description for complexes **3a-b**.

More insights into the structure and bonding in these complexes were gained by density functional theory (DFT) calculations, and the optimized structure of **3b** is depicted in Figure 2c. In complexes **3a-b**, the iron fragment adopts a pseudo-octahedral coordination geometry in an all-cis configuration of the hydride and phosphine ligands, respectively (**3b**: L-Fe-L = 83.1–101.6°; L = H, PMe_3_). Only the Al metalloligand deviates from this geometry being bent away from its axial position by about 45°. The calculated structures also reflect the short Fe···Al distances observed in the solid-state structure of **3b** (Figure 3b). The Al–H bonds in these species appear to be significantly longer in comparison to the parent dibromide complexes (*e.g.*, 1.838 *vs* 2.045 Å), and the Wiberg bond indices (WBIs) for the Fe···Al bond drastically increase from 0.14 in **2b** to 0.55 in **3b**.

These findings are further underpinned by QTAIM calculations (Figure 3c).^36^ In **2b**, bond critical points (BCPs) are found between Fe and H and Al and H but not between Fe and Al. In **3b**, the QTAIM analysis reveals BCPs between Fe and Al and Fe and H, whereas no BCPs are found between Al and H. These data suggest the presence of a direct metal–metal bond due to a preceding double Al–H bond activation at the transition metal center.^30,32−35^

Analysis of the NPA charges in **3b** reveals significant polarization of the Fe–Al bond showing values of −0.97 for Fe and 1.25 for Al, while negative charges on the hydrides are low (−0.18/0.18). The large negative charge accumulation on Fe likely results from the strong electron-donating nature of both the two hydrides and aluminylene ligand. This assumption is supported by extended transition state-natural orbitals for chemical valence (ETS-NOCV) calculations^37^ on **3b** revealing that the donation from the hydrides and the aluminylene ligand to iron accounts for more than 64% (Δρ_1_ = −74.7 kcal/mol) of the total orbital interaction energy (Δ*E*_orb_ = −117.1 kcal/mol), whereas backdonation from the Fe–H bonds to the empty Al p-orbitals was identified as the second (Δρ_2_ = −16.6 kcal/mol, 14%) and third (Δρ_3_ = −14.2 kcal/mol, 12%) largest contributions to Δ*E*_orb_ (see Table S5 in the Supporting Information). These secondary interactions are likely responsible for the bent position of the aluminylene metalloligand.

**Figure 4:**
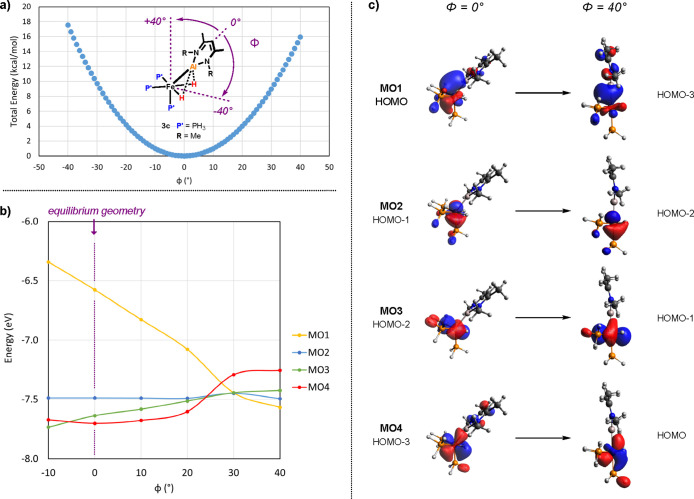
(a) Energy profile for the relaxed scan of the Al–Fe–P_ax_ angle in simplified model complex **3c** [ωB97X//6–31g** (H,C,N,P)/SDDAll (Fe,Al)]. (b) Energies of the occupied frontier orbitals in **3c** as function of the Al–Fe–P_ax_ bending angle. (c) Orbital isosurfaces (isovalue = 0.05) at 0° (equilibrium structure) and 40° (metalloligand in the axial position) bending angles.

Natural bond orbital (NBO) calculations were used to gain further insights into the bonding in these complexes. For the sake of clarity, we considered simplified model complex **3c** (P’ = PH_3_, R = Me) which, however, gives rise to highly similar frontier MOs as in **3a-b** (see Table S6 in the Supporting Information). The NBO analysis identifies a σ-bond between Fe (4s, 40.8%, 3d 57.8%) and Al (3s 73.6%, 3p 26.4%) as the main contributor to the HOMO, while the LUMO + 2 comprises an empty p-orbital on aluminum and possesses antibonding character with respect to the Fe–Al σ-bond. The HOMO is significantly higher (about 1.0 eV) in energy than the lower MOs which are predominantly non-bonding and possess largely 3d character (HOMO-1 to HOMO-3). This difference appears to be a consequence of the distorted coordination geometry and vanishes when the metalloligand is moved toward the axial position (Figure 4).

### Intramolecular C–H Activation

**Figure 5:**
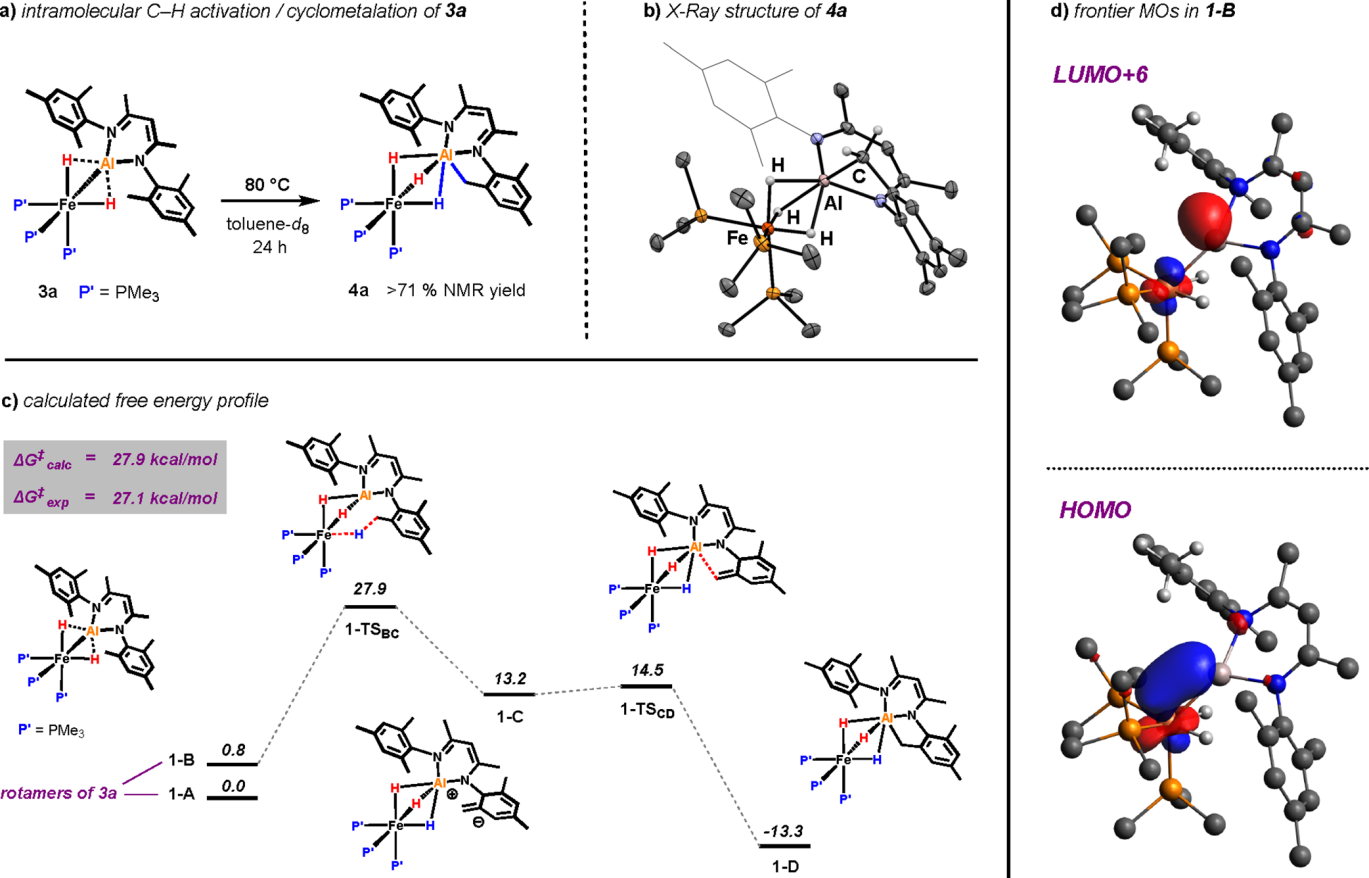
(a) Intramolecular C–H activation of **3a**. (b) X-ray structure of **4a**. (c) calculated free energy profile for the conversion of **3a** to **4a** [B3PW91/PCM (toluene)/D3//6–31g** (H,C,N,P)/SDDAll (Fe,Al)]. Energies are given in kcal/mol. (d) Acceptor (LUMO + 6) and donor (HOMO) orbitals in intermediate **1-B**.

Complex **3a** was found to undergo an intramolecular C–H activation affording cyclometalated complex **4a** (Figure 5a). Heating a toluene-*d*_8_ solution of **3a** to 80 °C results in slow formation of a new species as revealed by a growing new singlet resonance at δ_P_ = 30.0 ppm in the ^31^P NMR spectrum. In the ^1^H NMR, **4a** gives rise to a new hydride signal (broadened quartet) that integrates to 3H. The reaction was monitored over time, and complete consumption of the starting material was observed after 24 h. The new species was formed in 71% NMR yield. Crystals suitable for X-ray diffraction could be grown by diffusion of tetramethylsilane into a saturated solution of *n*-pentane, and the solid-state structure of **4a** is depicted in Figure 5b. Heating a solution of **3b** in toluene-*d*_8_ to 80 °C only results in the slow decomposition of this complex and the formation of untraceable species.

More insights into the conversion of **3a** to **4a** were gained from a combination of kinetic experiments and DFT calculations.^13^ Experimentally, the reaction was found to be first-order with respect to **3a**. An Eyring analysis over a temperature range of 60–100 °C gave the activation parameters Δ*H*^‡^ = 23.4 ± 0.5 kcal mol^–1^ and Δ*S*^‡^ = 12.5 ± 1.3 cal mol^–1^ K^–1^ which correspond to an associated Δ*G*_298K_^‡^ = 27.1 ± 0.8 kcal mol^–1^. DFT calculations were benchmarked against the experimental data. Best results were obtained by using the B3PW91 functional and a 6–31g** (H,C,N,P)/SDDAll (Fe,Al) basis set. Solvent (PCM, Benzene) and dispersion corrections (D3) were directly included in the optimization of the stationary points.

The calculated free energy profile for the intramolecular C–H activation is shown in Figure 5c. The reaction sequence is initiated by a slight rotation of the aluminylene ligand around the Fe···Al axis (**1-A** to **1-B**) followed by a deprotonation of the mesityl CH_3_ group by the iron metal center (**1-TS**_**BC**_). Intermediate **1-C** features the Fe-(μ-H_3_)-Al bridging motif and the deprotonated mesityl residue. Another slight conformational change initiates the Al–C bond formation (**1-TS**_**CD**_) and leads to the cyclometalated product (**1-D**). Formation of the product is exergonic by −13.3 kcal mol^–1^ with an overall barrier of 27.9 kcal mol^–1^ being in good agreement with the experimental value (27.1 ± 0.8 kcal mol^–1^).

Analysis of the frontier MOs reveals that the HOMO in the ground state of **3a** (Figure 5d) acts as the electron donor for the reductive deprotonation of the CH_3_ group in **1-TS**_**BC**_, while an empty p-orbital at Al (LUMO + 6, Figure 5d) acts as the electron acceptor for the deprotonated CH_2_ group (**1-TS**_**CD**_) to form the Al–C bond in the final product (**1-D**).

### Intermolecular C–H Activation

**Figure 6:**
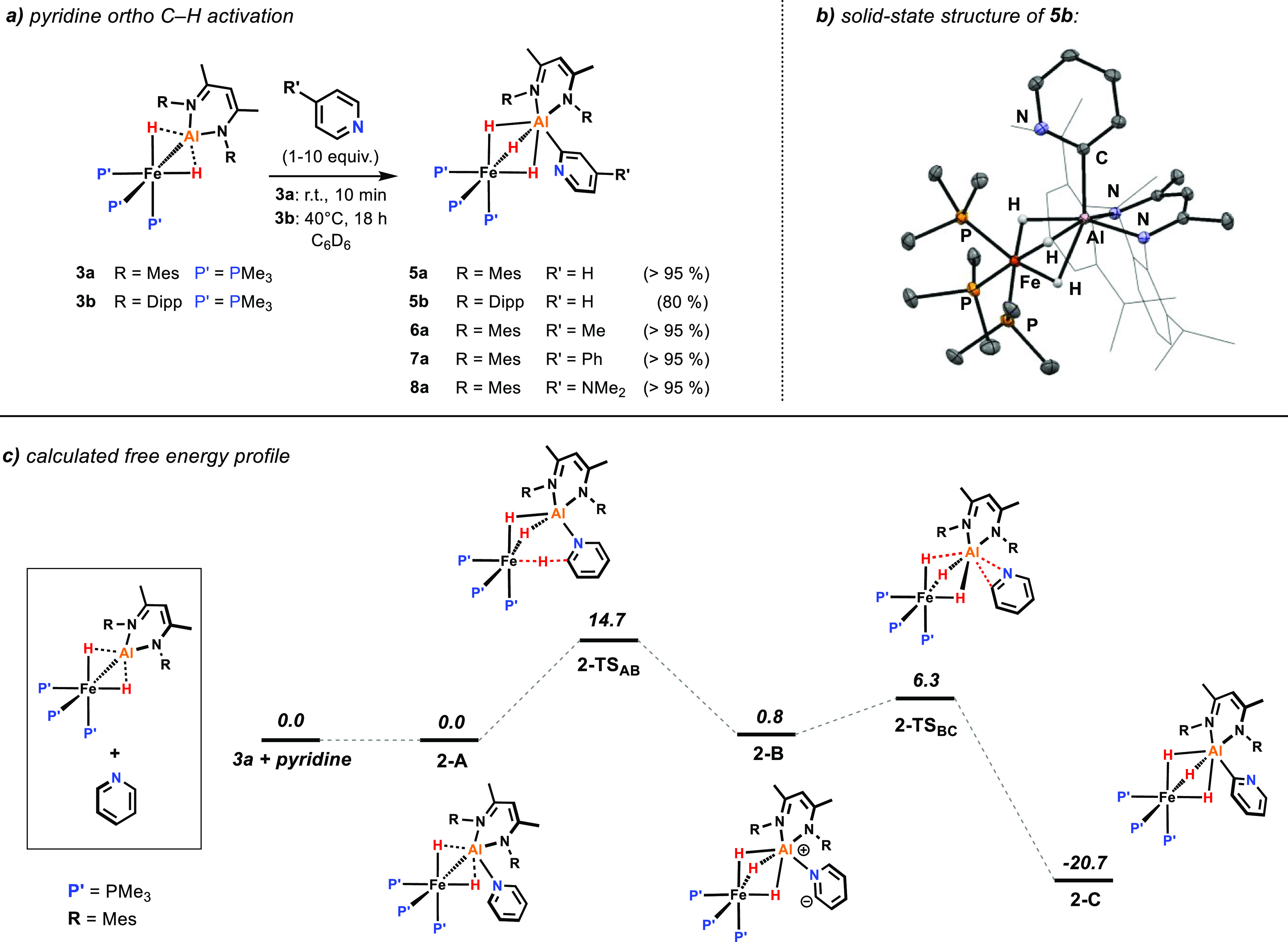
(a) Pyridine ortho C–H activation with **3a-b** (NMR yields are given in parentheses), (b) X-ray structure of **5b**, and (c) calculated free energy profile for the conversion of **3a** to **5a** [B3PW91/PCM (benzene)/D3//6–31g** (H,C,N,P)/SDDAll (Fe,Al)]. Energies are given in kcal/mol.

Complexes **3a** and **3b** are also capable of promoting intermolecular C–H activation. For example, **3a** readily reacts with pyridine (1 equiv, C_6_D_6_, room temperature) resulting in the selective C–H activation in the 2-position of the heterocycle (>95% NMR yield, Figure 6a). Like **4a**, **5a** exhibits a sharp singlet resonance in the ^31^P{^1^H} NMR spectrum at δ_P_ = 29.6 ppm and a broadened quartet hydride signal at δ_H_ = −15.33 ppm in the ^1^H NMR spectrum. The reaction of **3b** with pyridine required more forcing reaction conditions (40 °C, 10 equiv of pyridine, 18 h) but led to analogue product **5b** in about 80% NMR yield. Crystals suitable for X-ray diffraction could be obtained for **5b,** and the solid-state structure confirms the ortho C–H activation and C–Al bond formation in the 2-position of the pyridine heterocycle (Figure 6b).

A small array of substrates was investigated. Employing pyridines with different substituents in the 4-postion (R = Me, Ph, and NMe_2_) under the same conditions did not show any change in the reactivity or selectivity of the C–H activation with **3a**, in all cases giving products that arise from substitution at the 2-position (Figure 6a). On the other hand, 2-phenylpyridine did not react with **3a**, presumably due to steric interference of the phenyl substituent with the ligand system on aluminum.

DFT calculations on the ortho C–H activation of pyridine suggest a similar reaction pathway as for the intramolecular C–H activation of **3a** (Figure 6c). Again, a two-step mechanism has been identified that proceeds *via* the deprotonation of the pyridine substrate. Coordination of the pyridine nitrogen to the aluminum center controls the site selectivity of the reaction. NBO calculations reveal that the coordination of pyridine also facilitates bond breaking. In **2-A**, the Fe–Al bond becomes more polarized in comparison to **1-B**, and the WBI drops (see Tables S7 and S8 in the Supporting Information). The following transition state **2-TS**_**AB**_ is significantly lower in energy than for the intramolecular deprotonation of the mesityl CH_3_ group in **3a** (14.7 *vs* 27.9 kcal/mol) being in line with a fast reaction at room temperature. Both the intermediates after coordination (**2-A**, 0.0 kcal/mol) and deprotonation (**2-B**, 0.8 kcal/mol) of the pyridine appear to be almost thermoneutral in comparison to the starting materials. However, a final switch from N- to C-coordination of the deprotonated pyridine is facile (**2-TS**_**BC**_, a barrier of 5.5 kcal/mol) and highly exergonic due to formation of the Al–C bond in **2-C** (−20.7 kcal/mol).

A comparison of the reaction rates using an excess of pyridine or pyridine-*d*_5_ in two independent reactions gave an unusually large kinetic isotope effect (KIE) of 14.0 ± 0.2 at 297 K. The experimentally observed *k*_H_/*k*_D_ value likely results from a large classical KIE including a significant contribution of quantum tunneling.^38^ The rate-determining elementary step (**2-A** to **2-B**) is nearly thermoneutral with a centered transition state along the reaction coordinate, and thus, a maximum primary KIE (up to 7–8) may be expected.^38,39^ On the other hand, KIEs greater than 10 are generally considered to be caused by quantum tunneling and are diagnostic for proton transfer reactions.^40−43^ Both assumptions are in agreement with the calculated mechanism in Figure 6c and support a non-oxidative addition pathway.

## Conclusions

In summary, we described two well-defined bimetallic Fe–Al complexes that possess a distorted octahedral geometry. This distortion leads to an unusual ground state destabilization and raises the energy of the HOMO (the Fe–Al bond). Consequently, the HOMO becomes accessible for an encounter substrate and leads to an enhanced reactivity of these complexes including intramolecular sp^2^ C–H activation and the selective ortho C–H activation of pyridine substrates. These reactions were found to follow a novel cooperative mechanism in which a reductive deprotonation of the C–H bond was identified as a key elementary step. We could show that the Fe–Al bonds in these complexes are highly polarized and react as a bimetallic FLP in which the destabilized HOMO acts as a Lewis donor orbital, while empty p-orbitals on Al serve as Lewis acceptors. The reaction leads to the formation of a Fe–H and Al–C bonds. These results may lay the foundation for the rational design of future catalytic systems with prospects in the field of base-metal catalysis.

## Supporting Information Available

Synthetic procedures, kinetic experiments, NMR spectra of all compounds, crystallographic data, and computational methods (PDF)
Cartesian coordinates of the DFT-optimized structures (XYZ)

## Supplementary Material

ja2c02662_si_001.pdf

ja2c02662_si_002.xyz

## Accession Codes

CCDC 2127534–2127539 contain the supplementary crystallographic data for this paper. These data can be obtained free of charge via www.ccdc.cam.ac.uk/data_request/cif, or by emailing data_request@ccdc.cam.ac.uk, or by contacting The Cambridge Crystallographic Data Centre, 12 Union Road, Cambridge CB2 1EZ, UK; fax: +44 1223 336033.
