# Supplementary material for: Cooperative C–H Bond Activation by a Low-Spin d6 Iron–Aluminum Complex
Source: J Am Chem Soc. 2022 May 5;144(19):8770–7. doi: 10.1021/jacs.2c02662 (PMC9121387; doi:10.1021/jacs.2c02662)
Supplement: Supplementary file 1 — ja2c02662_si_001.pdf [file ja2c02662_si_001.pdf]

Cooperative C–H Bond Activation by a Low-Spin d^6 Iron–Aluminium Complex

*Nikolaus Gorgas, Andrew J. P. White, and Mark R. Crimmin**
Department of Chemistry, Imperial College London, White City, London, W12 0BZ

1. General Experimental S2

2. Synthetic Procedures S2

3. X-Ray Data S12

4. Kinetic Experiments S16

5. Computational Methods S18

6. NMR Spectra of the Isolated Compounds S24

7. Selected IR Spectra S39

8. References S42

1. General Experimental

All manipulations were carried out using standard Schlenk-line and glovebox techniques under an inert atmosphere of argon or dinitrogen. A MBraun Labmaster glovebox was employed, operating at <0.1 ppm O_2 and <0.1 ppm H_2O. Solvents were dried over activated alumina from a SPS (solvent purification system) based upon the Grubbs design and degassed before use. Glassware was dried for 12 h at 120°C prior to use. C_6D_6 was dried over 3 Å molecular sieves and freeze-pump-thaw degassed thrice before use. Chemicals were purchased from Sigma Aldrich, Fluorochem, Alfa Aesar, and VWR. Pyridine was dried over CaH_2, distilled, and stored over activated 3 Å molecular sieves. **1a** and **1b** were synthesised according to literature procedures.[1] NMR Spectra were recorded on Bruker 400 MHz or 500 MHz at 298 K unless otherwise stated and values recorded in ppm. Data were processed in MestReNova software. Where needed, chemical shifts were assigned with the assistance of 2D NMR (HSQC, HMBC, COSY) spectra. IR spectra were recorded on an Agilent Cary630 ATR FTIR spectrometer. Elemental analyses were performed by Elemental Labs (https://www.elementallab.co.uk/).

2. Synthetic Procedures

*Synthesis of **2a**:*

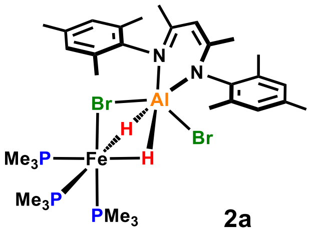

2a

In a glovebox, a 100 mL Schlenk-tube was charged with anhydrous $FeBr_2$ (324 mg, 1.50 mmol). A solution of trimethyl phosphine (493 µL, 4.80 mmol) in toluene (15 mL) was added resulting in the formation of a deep purple solution which was stirred for 5 min untill all solids were dissolved. **1a** (543 mg, 1.50 mmol) was dissolved in toluene (15 mL) and slowly added to the reaction solution over 5 min. Upon addition, the reaction becomes a clear reddish solution which was stirred for additional 20 min at room temperature. The solvent was removed in vacuo, the remaining solid washed with small portions of n-pentane and dried under high vacuum. Yield: 992 mg, 1.23 mmol, 82 %. Crystals suitable for X-ray diffraction were grown from a concentrated solution of **2a** in diethyl ether at -35 °C.

^{1}H NMR (400 MHz, C_6D_6, 298 K): δ 6.82 (s, 2H, Mes-C***H***), 6.79 (s, 2H, Mes-C***H***), 5.47 (s, 1H, BDI-C***H***), 3.07 (s, 6H, Mes-CC***H***$_3$), 2.48 (s, 6H, , Mes-CC***H***$_3$), 2.23 (s, 6H, Mes-CC***H***$_3$), 1.63 (s, 6H, BDI-C***H***$_3$), 1.30 (br, 9H, P-***C***H$_3$), 0.83 (m, 18H, P-***C***H$_3$), -13.08 (br, 2H, Fe-***H***-Al).

^{31}P{^{1}H} NMR (162 MHz, C_6D_6, 298 K): δ 47.8 (t, J_{PP} = 44.6 Hz, 1P), 16.6 (d, J_{PP} = 44.6 Hz, 2P).

^{13}C{^{1}H} NMR (101 MHz, C_6D_6, 298 K): δ 169.2 (2C, BDI-***C***N), 144.1 (2C, Mes-***C***N), 135.8 (2C, Mes-***C***CH$_3$), 135.3 (2C, Mes-***C***CH$_3$), 133.8 (2C, Mes-***C***CH$_3$), 129.3 (2C, Mes-***C***H), 129.1 (2C, Mes-***C***H), 100.9 (1C, BDI-***C***H), 24.2 (2C, BDI-***C***H$_3$), 22.5 (2C, Mes-C***C***H$_3$), 21.7 (m, P-***C***H$_3$), 20.5 (2C, Mes-C***C***H$_3$), 19.7 (2C, Mes-C***C***H$_3$).

Anal. Calc. ($C_{32}H_{58}AlBr_2FeN_2P_3$): C, 47.66; H, 7.25; N, 3.47. Found: C, 48.41; H, 7.11; N, 3.41.

Synthesis of ***2b:***

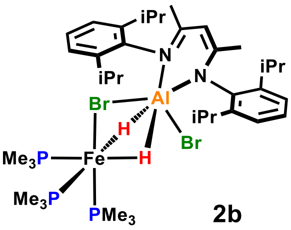

2b

2b was prepared analogously to **2a** starting from 173 mg of $FeBr_2$ (0.80 mmol), 247 µL (2.4 mmol) of trimethyl phosphine and 357 mg of **1b** (0.8 mmol) in toluene (10 mL). The crude product was washed with pentane (3 x 2 mL) and dried under vacuum to give pure **2b** as a bright orange powder. Yield: 520 mg (0.71 mmol, 89 %). Crystals suitable for X-ray diffraction could be grown from a concentrated solution of **2b** in toluene at -35 °C.

^{1}H NMR (400 MHz, C_6D_6, 298 K): δ 7.29 – 7.18 (m, 6H, Dipp-C***H***), 5.31 (s, 1H, BDI-C***H***), 4.80 (hept, *J* = 6.8 Hz, 2H, $(CH_3)_2C$***H***), 3.79 (hept, *J* = 6.8 Hz, 2H, Dipp-C***H***$(CH_3)_2$), 1.71 (s, 6H, Dipp-CH(C***H***$_3$)$_2$), 1.65 (d, *J* = 6.8 Hz, 6H, Dipp-CH(C***H***$_3$)$_2$), 1.63 (d, *J* = 6.8 Hz, 6H, Dipp-CH(C***H***$_3$)$_2$), 1.43 (d, *J* = 6.8 Hz, 6H, Dipp-CH(C***H***$_3$)$_2$), 1.21 (d, J_{HP} = 8.3 Hz, 9H, P-C***H***$_3$), 1.19 (d, *J* = 6.8 Hz, 6H, Dipp-CH(C***H***$_3$)$_2$), 0.87 (m, 18H, P-C***H***$_3$), -13.20 (br, 2H, Fe-***H***-Al).

^{31}P{^{1}H} NMR (162 MHz, C_6D_6, 298 K): δ 44.5 (t, J_{PP} = 43.8 Hz, 1P), 15.4 (d, J_{PP} = 43.8 Hz, 2P).

^{13}C{^{1}H} NMR (101 MHz, C_6D_6, 298 K): δ 169.7 (2C, BDI-***C***N), 146.0 (2C, Dipp-***C***CH$(CH_3)_2$), 145.8 (2C, Dipp-***C***CH$(CH_3)_2$), 145.3 (2C, Dipp-***C***N), 126.2 (2C, Dipp-***C***H), 124.2 (2C, Dipp-***C***H), 123.9 (2C, Dipp-***C***H), 100.7 (1C, BDI-***C***H), 28.8 (2C, Dipp-C***C***H$(CH_3)_2$), 28.4 (2C, Dipp-C***C***H$(CH_3)_2$), 26.1 (2C, BDI-C***C***H$_3$), 25.7 (2C, Dipp-CCH(***C***H$_3$)$_2$), 25.5 (2C, Dipp-CCH(***C***H$_3$)$_2$), 25.2 (2C, Dipp-CCH(***C***H$_3$)$_2$), 24.6 + 24.4 (5C, Dipp-CCH(***C***H$_3$)$_2$ + P-C***H***$_3$), 22.1 (m, 6C, P-C***H***$_3$).

Anal. Calc. ($C_{38}H_{70}AlBr_2FeN_2P_3$): C, 51.25; H, 7.92; N, 3.15. Found: C, 51.36; H, 7.91; N, 3.39.

*Synthesis of **3a**:*

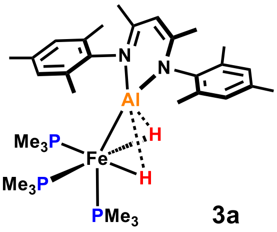

3a

In a glovebox, Magnesium turning (~ 30 mg, 1.23 mmol) were added to a solution of **2a** (200 mg, 0.248 mmol) in THF (5.0 mL). The mixture was stirred for 2 h during which time the solution became dark red/orange. The unreacted magnesium turnings were filtered off and the solvent removed under reduced pressure. The resulting solid was extracted with *n*-pentane (5 x 2.0 mL), the combined extracts filtered, and the solvent removed *in vacuo* to give **3a** as dark orange crystalline solid. Yield: 148 mg (0.228 mmol, 92 %). Crystals suitable for X-ray diffraction were obtained by slow evaporation of a concentrated solution in n-pentane.

^{1}H NMR (400 MHz, C_6D_6, 298 K): δ 6.85 (s, 4H, Mes-C***H***), 4.97 (s, 1H, BDI-C***H***), 2.34 (s, 12H, Mes-CC***H***$_3$), 2.22 (s, 6H, Mes-CC***H***$_3$), 1.37 (s, 6H, BDI-C***H***$_3$), 1.16 (s, 27H, P-C***H***$_3$), -16.31 (q, J_{HP} = 20.9 Hz, 2H, Fe-***H***-Al).

^{31}P{^{1}H} NMR (162 MHz, C_6D_6, 298 K): δ 26.1 (s, 3P).

^{13}C{^{1}H} NMR (101 MHz, C_6D_6, 298 K): δ 167.4 (2C, BDI-***C***N), 144.2 (2C, Mes-***C***N), 135.0 (2C, Mes-***C***CH$_3$), 133.2 (4C, Mes-***C***CH$_3$), 129.7 (4C, Mes-***C***H), 99.2 (1C, BDI-***C***H), 30.0 (m, 9C, P-***C***H$_3$), 23.5 (2C, BDI-***C***H$_3$), 20.6 (2C, Mes-C***C***H$_3$), 19.4 (4C, Mes-C***C***H$_3$).

Anal. Calc. ($C_{32}H_{58}AlFeN_2P_3$): C, 59.44; H, 9.04; N, 4.33. Found: C, 58.07; H, 8.96; N, 4.45.

*Synthesis of **3b**:*

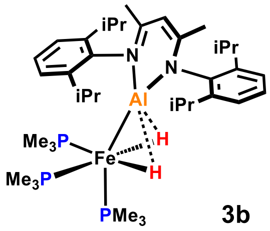

3b

3b was obtained following the same procedure as described for the synthesis of **3a** but starting from **2b** (200 mg, 0.225 mmol) in THF (5.0 mL). **3b** was obtained as dark orange crystalline solid. Yield: 136 mg (0.186 mmol, 83 %). Crystals suitable for X-ray diffraction were obtained by slow evaporation of a concentrated solution in n-pentane.

^{1}H NMR (400 MHz, C_6D_6, 298 K): δ 7.27 – 7.21 (m, 2H, Dipp-C***H***), 7.20 – 7.17 (m, 4H, Dipp-C***H***), 4.95 (s, 1H, BDI-C***H***), 3.52 (hept, *J* = 6.8 Hz, 4H, Dipp-C***H***(CH_3)$_2$), 1.59 (d, *J* = 6.8 Hz, 12H, Dipp-CH(C***H***$_3$)$_2$), 1.46 (s, 6H, BDI-C***H***$_3$), 1.14 (d, *J* = 6.8 Hz, 12H, Dipp-CH(C***H***$_3$)$_2$), 1.11 (s, 27H, P-C***H***$_3$), -16.53 (q, J_{HP} = 21.1 Hz, 2H, Fe-***H***-Al).

^{31}P{^{1}H} NMR (162 MHz, C_6D_6, 298 K): δ 23.3 (s, 3P).

^{13}C{^{1}H} NMR (101 MHz, C_6D_6, 298 K): δ 167.9 (2C, BDI-***C***N), 144.7 (2C, Dipp-***C***N), 143.5 (4C, Dipp-***C***CH(CH_3)$_2$), 126.6 (2C, Dipp-***C***H), 124.6 (4C, Dipp-***C***H), 98.8 (1C, BDI-***C***H), 30.0 (m, 9C, P-***C***H$_3$), 28.7 (4C, Dipp-C***C***H(CH_3)$_2$), 25.3 (4C, Dipp-CCH(***C***H$_3$)$_2$), 24.8 (2C, BDI-***C***H$_3$), 24.3 (4C, Dipp-CCH(***C***H$_3$)$_2$).

Anal. Calc. ($C_{38}H_{70}AlFeN_2P_3$): C, 62.46; H, 9.66; N, 3.83. Found: C, 62.00; H, 9.64; N, 3.82.

*Synthesis of **4a**:*

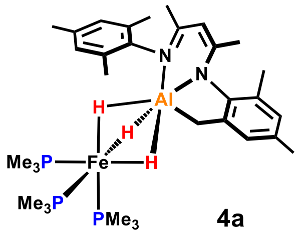

4a

In a glovebox, **3a** (60 mg, 0.093 mmol) was dissolved in toluene-d_8 and the solution transferred to a J-Young NMR tube. The NMR tube was heated for 24 h to 80 °C in an isothermal bath during which time the solution changed from dark red-orange to yellow. NMR analysis of the reaction mixture revealed complete consumption of the starting material and formation of **5a** in 71 % yield. The reaction solution was transferred back to a glovebox and the solvent was removed under reduced pressure. The residue was dissolved in a minimum amount of n-pentane and the solution layered with tetramethylsilane. Slow diffusion of tetramethylsilane into the n-pentane solution at -35 °C led to the formation of pale-yellow crystals (suitable for X-ray diffraction). The mother liquor was decanted, the crystals washed with small amounts of cold tetramethylsilane and dried under vacuum. Isolated yield: 25.3 mg (0.039 mmol, 42 %).

^{1}H NMR (400 MHz, C_6D_6, 298 K): δ 6.84 (s, 1H, Mes-C***H***), 6.80 (s, 1H, Mes-C***H***), 6.77 (s, 1H, Mes-C***H***), 6.75 (s, 1H, Mes-C***H***), 4.90 (s, 1H, BDI-C***H***), 2.48 (s, 3H, Mes-CC***H***$_3$), 2.45 (s, 3H, Mes-CC***H***$_3$), 2.33 (s, 3H, Mes-CC***H***$_3$), 2.25 (d, J = 11.8 Hz, 1H, Mes-CC***H***$_2$-Al), 2.23 (s, 3H, Mes-CC***H***$_3$), 2.20 (s, 3H, Mes-CC***H***$_3$), 1.76 (s, 3H, BDI-C***H***$_3$), 1.50 (s, 2H, BDI-C***H***$_3$), 1.4 (d, J = 11.8 Hz, 1H, Mes-CC***H***$_2$-Al), 0.92 (m, 27H), -15.68 (br, 3H).

^{31}P{^{1}H} NMR (162 MHz, C_6D_6, 298 K): δ 30.0 (s, 3P).

^{13}C{^{1}H} NMR (101 MHz, C_6D_6, 298 K): δ 169.5 (1C, BDI-***C***N), 166.1 (1C, BDI-***C***N), 145.8 (1C, Mes-***C***N), 144.7 (1C, Mes-***C***CH$_2$-Al), 142.5 (1C, Mes-***C***N), 134.3 (1C, Mes-***C***CH$_3$), 134.1 (1C, Mes-***C***CH$_3$), 133.6 (1C, Mes-***C***CH$_3$), 133.5 (1C, Mes-***C***CH$_3$), 129.6 (1C, Mes-***C***H), 129.1 (1C, Mes-***C***H), 128.7 (1C, Mes-***C***H), 128.3 (1C, Mes-***C***CH$_3$), 124.3 (1C, Mes-***C***H), 97.8 (1C, BDI-***C***H), 28.1 (located from the ^{1}H/^{13}C HSQC spectrum, 1C, Mes-C***C***H$_2$-Al), 26.2 (m, 9C, P-***C***H$_3$), 24.1 (1C, BDI-***C***H$_3$), 22.9 (1C, BDI-***C***H$_3$), 21.3 (1C, Mes-C***C***H$_3$), 20.6 (1C, Mes-C***C***H$_3$), 20.5 (1C, Mes-C***C***H$_3$), 19.3 (1C, Mes-C***C***H$_3$), 19.1 (1C, Mes-C***C***H$_3$).

Anal. Calc. ($C_{32}H_{58}AlFeN_2P_3$): C, 59.44; H, 9.04; N, 4.33. Found: C, 58.21; H, 8.87; N, 4.31.

*Synthesis of **5a**:*

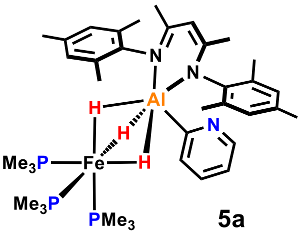

5a

In a glovebox, a stock solution of pyridine in C_6D_6 (0.1 M, 230 µL, 0.023 mmol) was added to a solution of **3a** (15 mg, 0.023 mmol) in C_6D_6 (0.6 mL) and transferred to a J. Young NMR tube. Within 10 min, the colour of the reaction solution changed from red-orange to yellow. NMR analysis of the reaction solution revealed formation of **5a** in >95 % yield. The NMR tube was transferred back to the glovebox and the solvent removed under reduced pressure. The remaining sticky solid was triturated in n-pentane and the solvent again removed under reduced pressure to afford **5a** as foamy solid which was further dried under high vacuum. Isolated yield: 14.9 mg (0.020 mmol, 89 %).

^{1}H NMR (400 MHz, C_6D_6, 298 K): δ 9.00 (d, *J* = 4.6 Hz, 1H, Py-C***H***), 8.22 (d, *J* = 7.6 Hz, 1H, Py-C***H***), 7.34 (td, *J* = 7.6, 1.8 Hz, 1H, Py-C***H***), 6.88 (m, 1H, Py-C***H***), 6.78 (s, 2H, Mes-C***H***), 6.75 (s, 2H, Mes^{3,5}-C***H***), 5.22 (s, 1H, {(CH_3)C}$_2$C***H***), 2.47 (s, 6H, Mes-CC***H***$_3$), 2.21 (s, 6H, Mes-CC***H***$_3$), 2.17 (s, 6H, Mes^4-CC***H***$_3$), 1.51 (s, 6H, {(C***H***$_3$)C}$_2$CH), 1.00 (s, 27H, P-C***H***$_3$), -15.33 (s, 3H, Fe-***H***-Al).

^{31}P{^{1}H} NMR (162 MHz, C_6D_6, 298 K): δ 29.6 (s, 3P).

^{13}C{^{1}H} NMR (101 MHz, C_6D_6, 298 K): δ 167.8 (2C, BDI-***C***N), 147.8 (Py-***C***H), 146.3 (2C, Mes-***C***N), 136.0 (2C, Mes-***C***CH$_3$), 133.8 (2C, Mes-***C***CH$_3$), 133.1 (2C, Mes-***C***CH$_3$), 132.8 (Py-***C***H), 129.8 (2C, Mes-***C***H), 129.7(Py-***C***H), 129.0 (2C, Mes-***C***H), 119.1 (Py-***C***H), 100.3 (1C, BDI-***C***H), 25.8 (m, 9C, P-***C***H$_3$), 23.9 (2C, BDI-***C***H$_3$), 21.1 (2C, Mes-C***C***H$_3$), 20.5 (2C, Mes-C***C***H$_3$), 20.2 (2C, Mes-C***C***H$_3$). The Py^2-***C***-Al resonance could not be observed due to line-broadening associated with coupling to the quadrupolar I = 5/2 ^{27}Al nucleus.

Anal. Calc. ($C_{37}H_{63}AlFeN_3P_3$): C, 61.24; H, 8.75; N, 5.79. Found: C, 60.46; H, 8.35; N, 5.79.

*Synthesis of **5b**:*

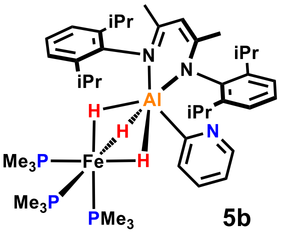

5b

In a glovebox, pyridine (65 µL, 0.840 mmol) was added to a solution of **3b** (60 mg, 0.084 mmol) in C_6D_6 (1.2 mL) and transferred to a J. Young NMR tube. The NMR tube was heated for 24 h to 40 °C in an isothermal bath during which time the solution changed from dark red-orange to dark greenish. NMR analysis of the reaction mixture revealed complete consumption of the starting material and formation of **5a** in about 80 % yield. The reaction solution was transferred back to a glovebox and the solvent was removed under reduced pressure. The residue was recrystallised from *n*-pentane (2 mL) at -35 °C affording a dark greenish solution and an off-white crystalline solid. The supernatant solution was decanted, the solid carefully washed with small amounts of *n*-pentane (4 x 0.5 mL) and dried under vacuum. Isolated yield: 39.5 mg (0.049 mmol, 58 %).

^{1}H NMR (400 MHz, C_6D_6, 298 K): δ 8.89 (d, *J* = 4.2 Hz, 1H, Py-C***H***), 8.15 (d, *J* = 7.5 Hz, 1H, Py-C***H***), 7.33 (t, *J* = 7.5 Hz, 1H, Py-C***H***), 7.26 – 7.12 (6H, Dipp-C***H***, overlapped by the residual signal of C_6D_6), 6.89 (m, 1H, Py-C***H***), 5.13 (s, 1H, BDI-C***H***), 3.63 (two overlapping hept, *J* = 6.9 Hz, 4H, Dipp-C***H***$(CH_3)_2$), 1.53 (s, 6H, BDI-C***H***$_3$), 1.51 (d, *J* = 6.9 Hz, 6H, Dipp-CCH(***C***$H_3)_2$), 1.35 (d, *J* = 6.9 Hz, 6H, Dipp-CCH(***C***$H_3)_2$), 1.23 (d, *J* = 6.9 Hz, 6H, Dipp-CCH(***C***$H_3)_2$), 1.02 – 0.92 (overlapping signals, 33H, P-C***H***$_3$ + Dipp-CCH(***C***$H_3)_2$), -15.59 (br, 3H, Fe-***H***-Al).

^{31}P{^{1}H} NMR (162 MHz, C_6D_6, 298 K): δ 27.1 (s, 3P).

^{13}C{^{1}H} NMR (101 MHz, C_6D_6, 298 K): δ 169.1 (2C, BDI-***C***N), 147.5 (2C, Dipp-***C***N), 146.9 (Py-***C***H), 146.0 (2C, Dipp-***C***$CH(CH_3)_2$), 143.2 (2C, Dipp-***C***$CH(CH_3)_2$), 132.5 (Py-***C***H), 129.2 (Py-***C***H), 125.8 (2C, Dipp-***C***H), 124.5 (2C, Dipp-***C***H), 123.9 (2C, Dipp-***C***H), 119.0 (Py-***C***H), 100.6 (1C, Dipp-***C***H), 28.3 (2C, Dipp-C***C***$H(CH_3)_2$), 27.8 (2C, Dipp-C***C***$H(CH_3)_2$), 26.4 (m, P-***C***H_3), 25.5 + 25.4 (4C, Dipp-CCH(***C***$H_3)_2$ + BDI-***C***H_3), 25.2 (2C, Dipp-CCH(***C***$H_3)_2$), 25.1 (2C, Dipp-CCH(***C***$H_3)_2$), 24.6 (2C, Dipp-CCH(***C***$H_3)_2$). The Py^2-***C***-Al resonance could not be observed due to line-broadening associated with coupling to the quadrupolar I = 5/2 ^{27}Al nucleus.

Anal. Calc. ($C_{43}H_{75}AlFeN_3P_3$): C, 63.77; H, 9.34; N, 5.19. Found: C, 64.59; H, 9.10; N, 5.02.

*Synthesis of **6a**:*

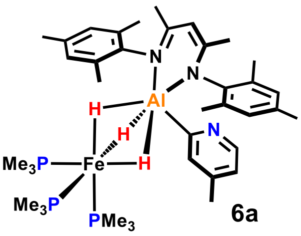

6a

In a glovebox, a stock solution of 4-methylpyridine in C_6D_6 (0.1 M, 230 µL, 0.023 mmol) was added to a solution of **3a** (15 mg, 0.023 mmol) in C_6D_6 (0.6 mL) and transferred to a J. Young NMR tube. Within 10 min, the colour of the reaction solution changed from red-orange to yellow. NMR analysis of the reaction solution revealed formation of **6a** in > 95 % yield. The NMR tube was taken back to the glovebox, the solution transferred into a 10 mL scintillation vial, and the solvent removed under reduced pressure. The residue was triturated in *n*-pentane and the solvent again removed under reduced pressure to afford **6a** as a yellow solid. Yield: 16.0 mg (0.022 mmol, 94 %).

^{1}H NMR (400 MHz, C_6D_6, 298 K): δ 8.93 (d, *J* = 4.9 Hz, 1H, Py-C***H***), 8.00 (s, 1H, Py-C***H***), 6.79 (s, 2H, Mes-C***H***), 6.76 (s, 3H, Mes-C***H*** + Py-C***H***), 5.25 (s, 1H, BDI-C***H***), 2.48 (s, 6H), 2.26 (s, 6H, Mes-C***H***3), 2.21 (s, 3H, Mes-C***H***3), 2.18 (s, 6H, Mes-C***H***3), 1.54 (s, 6H, BDI-C***H***3), 1.00 (m, 27H, P-C***H***3), -15.33 (br q, *J* = 18.2 Hz, 3H, Fe-***H***-Al).

^{31}P{^{1}H} NMR (162 MHz, C_6D_6, 298 K): δ 29.6 (s, 3P).

^{13}C{^{1}H} NMR (101 MHz, C_6D_6, 298 K): δ 167.8 (2C, BDI-***C***N), 147.8 (1C, Py-***C***H), 146.3 (2C, Mes-***C***N), 138.8 (1C, Py-***C***CH3), 136.1 (2C, Mes-***C***CH3), 134.3 (1C, Py-***C***H), 133.8 (2C, Mes-***C***CH3), 133.2 (2C, Mes-***C***CH3), 129.8 (2C, Mes-***C***H), 129.0 (2C, Mes-***C***H), 120.1 (1C, Py-***C***H), 100.4 (1C, BDI-***C***H), 25.8 (m, 9C, P-***C***H3), 23.9 (2C, BDI-***C***H3), 21.5 (1C, Py-C***C***H3), 21.2 (2C, Mes-C***C***H3), 20.5 (2C, Mes-C***C***H3), 20.2 (2C, Mes-C***C***H3). The Py^2-***C***-Al resonance could not be observed due to line-broadening associated with coupling to the quadrupolar I = 5/2 ^{27}Al nucleus.

Anal. Calc. ($C_{38}H_{65}AlFeN_3P_3$): C, 61.70; H, 8.86; N, 5.68. Found: C, 60.40; H, 8.10; N, 5.44.

*Synthesis of **7a**:*

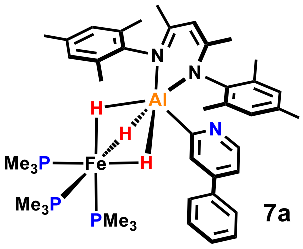

7a

In a glovebox, a stock solution of 4-phenylpyridine in C_6D_6 (0.1 M, 230 µL, 0.023 mmol) was added to a solution of **3a** (15 mg, 0.023 mmol) in C_6D_6 (0.6 mL) and transferred to a J. Young NMR tube. Within 10 min, the colour of the reaction solution changed from red-orange to pale yellow. NMR analysis of the reaction solution revealed formation of **7a** in > 95 % yield. The NMR tube was taken back to the glovebox, the solution transferred into a 10 mL scintillation vial, and the solvent removed under reduced pressure. The residue was triturated in *n*-pentane and the solvent again removed under reduced pressure to afford **7a** as an off-white solid. Yield: 14.8 mg (0.019 mmol, 83 %).

^{1}H NMR (400 MHz, C_6D_6, 298 K): δ 9.07 (d, *J* = 5.0 Hz, 1H, Py-C***H***), 8.55 (s, 1H, Py-C***H***), 7.81 (d, *J* = 7.5 Hz, 2H, Ph-C***H***), 7.29 (t, *J* = 7.6 Hz, 2H, Ph-C***H***), 7.19 (dd, *J* = 5.1, 1.9 Hz, 1H, Ph-C***H***), 6.78 (s, 2H, Mes-C***H***), 6.73 (s, 2H, Mes-C***H***), 5.23 (s, 1H, BDI-C***H***), 2.47 (s, 6H, Mes-CC***H***$_3$), 2.25 (s, 6H, Mes-CC***H***$_3$), 2.17 (s, 6H, Mes-CC***H***$_3$), 1.52 (s, 6H, BDI-C***H***$_3$), 1.01 (s, 27H, P-C***H***$_3$), -15.31 (br q, *J* = 19.9 Hz, 3H, Fe-***H***-Al).

^{31}P{^{1}H} NMR (162 MHz, C_6D_6, 298 K): δ 29.6 (s, 2P).

^{13}C{^{1}H} NMR (101 MHz, C_6D_6, 298 K): δ 168.0 (2C, BDI-***C***N), 148.5 (1C, Py-***C***H), 146.3 (2C, Mes-***C***N), 142.0 (1C, Py-***CC***-Ph), 141.0 (1C, Py-***CC***-Ph), 136.0 (2C, Mes-***C***CH$_3$), 133.8 (2C, Mes-***C***CH$_3$), 133.2 (2C, Mes-***C***CH$_3$), 130.8 (1C, Py-***C***H), 129.8 (2C, Mes-***C***H), 129.0 (2C, Mes-***C***H), 128.7 (2C, Ph-***C***H), 127.0 (2C, Ph-***C***H), 117.1 (1C, Ph-***C***H), 100.3 (1C, BDI-***C***H), 25.8 (m, 9C, P-***C***H$_3$), 23.8 (2C, BDI-***C***H$_3$), 21.3 (2C, Mes-C***C***H$_3$), 20.5 (2C, Mes-C***C***H$_3$), 20.1 (2C, Mes-C***C***H$_3$). The Py^2-***C***-Al resonance could not be observed due to line-broadening associated with coupling to the quadrupolar I = 5/2 ^{27}Al nucleus.

Anal. Calc. ($C_{38}H_{65}AlFeN_3P_3$): C, 64.42; H, 8.42; N, 5.24. Found: C, 65.11; H, 8.49; N, 5.03.

*Synthesis of **8a:***

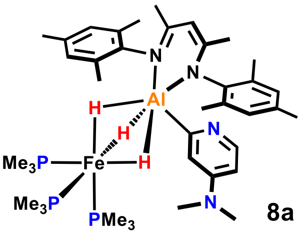

8a

In a glovebox, a stock solution of 4-dimethylaminopyridine in C_6D_6 (0.1 M, 230 µL, 0.023 mmol) was added to a solution of **3a** (15 mg, 0.023 mmol) in C_6D_6 (0.6 mL) and transferred to a J. Young NMR tube. Within 10 min, the colour of the reaction solution changed from red-orange to yellow. NMR analysis of the reaction solution revealed formation of **8a** in > 95 % yield. The NMR tube was taken back to the glovebox, the solution transferred into a 10 mL scintillation vial, and the solvent removed under reduced pressure. The residue was triturated in *n*-pentane and the solvent again removed under reduced pressure to afford **8a** as yellow solid. Yield: 15.8 mg (0.021 mmol, 89 %).

^{1}H NMR (400 MHz, C_6D_6, 298 K): δ 8.82 (d, *J* = 5.6 Hz, 1H, Py-C***H***), 7.64 (d, *J* = 2.8 Hz, 1H, Py-C***H***), 6.79 (s, 2H, Mes-C***H***), 6.75 (s, 2H, Mes-C***H***), 6.33 (dd, *J* = 5.6, 2.8 Hz, 1H, Py-C***H***), 5.25 (s, 1H, BDI-***C***H), 2.67 (s, 6H, Py-N(C***H***$_3$)$_2$), 2.50 (s, 6H, Mes-CC***H***$_3$), 2.32 (s, 6H, Mes-CC***H***$_3$), 2.18 (s, 6H, Mes-CC***H***$_3$), 1.54 (s, 6H, BDI-C***H***$_3$), 1.04 (s, 27H, P-C***H***$_3$), -15.28 (br q, *J* = 20.2 Hz, 3H, Fe-***H***-Al).

^{31}P{^{1}H} NMR (162 MHz, C_6D_6, 298 K): δ 29.8 (s, 2P).

^{13}C{^{1}H} NMR (101 MHz, C_6D_6, 298 K): δ 167.6 (2C, BDI-***C***N), 150.5 (1C, Py-***C***$_q$), 148.0 (1C, Py-***C***H), 146.5 (2C, Mes-***C***N), 136.3 (2C, Mes-***C***CH$_3$), 133.7 (2C, Mes-***C***CH$_3$), 133.1 (2C, Mes-***C***CH$_3$), 129.8 (s, 2H, Mes-C***H***), 128.9 (s, 2H, Mes-C***H***), 117.6 (1C, Py-***C***H), 104.1 (1C, Py-***C***H), 100.2 (1C, BDI-***C***H), 38.6 (2C, Py-N(***C***H$_3$)$_2$), 25.8 (m, P-***C***H$_3$), 23.9 (2C, BDI-***C***H$_3$), 21.5 (2C, Mes-C***C***H$_3$), 20.5 (2C, Mes-C***C***H$_3$), 20.2 (2C, Mes-C***C***H$_3$). The Py^2-***C***-Al resonance could not be observed due to line-broadening associated with coupling to the quadrupolar I = 5/2 ^{27}Al nucleus.

Anal. Calc. ($C_{38}H_{65}AlFeN_3P_3$): C, 63.77; H, 9.34; N, 5.19. Found: C, 64.59; H, 9.10; N, 5.02.

3. X-Ray Data

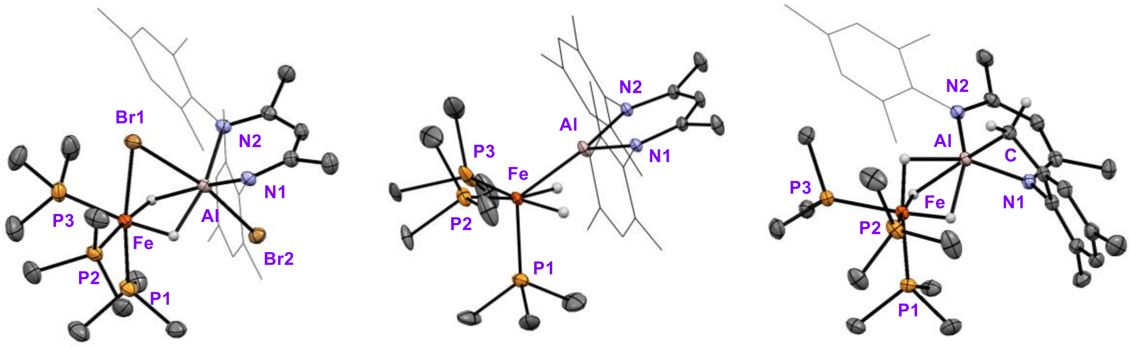

	2a		3a		4a	
**Fe---Al**	***2.453(1)***	***FSR = 1.02***	***2.2176(13)***	***FSR = 0.92***	***2.3495(7)***	***FSR = 0.98***
**Fe---P1**	*2.188(1)*		*2.1665(15)*		*2.1643(7)*	
**Fe---P2**	*2.226(1)*		*2.216(3)*		*2.1776(8)*	
**Fe---P3**	*2.239(1)*		*2.2176(13)*		*2.1768(7)*	
**Fe---X**	*2.4827(6)*	*X = Br1*	----------		----------	
**Al---N1**	*1.959(3)*		*1.949(2)*		*1.984(2)*	
**Al---N2**	*1.961(3)*		*1.949(2)*		*1.963(2)*	
**Al---X**	*2.464(1)*	*X = Br2*	----------		*2.048(2)*	*X = C*
	2.702(1)	*X = Br1*				

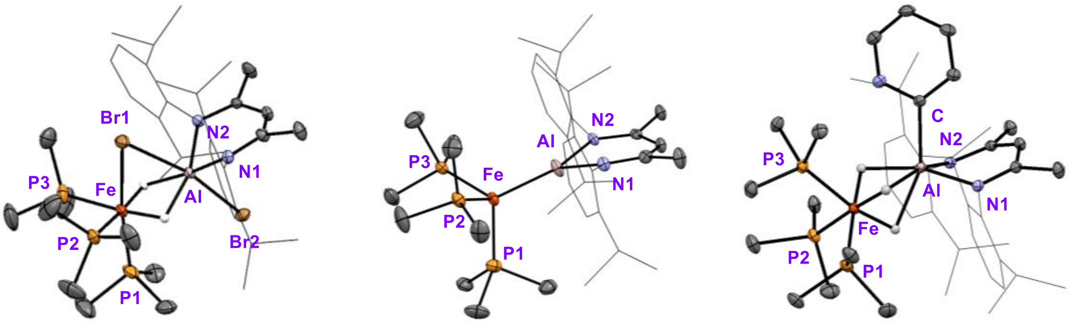

	2b		3b		5b	
**Fe---Al**	***2.459(1)***	***FSR = 1.02***	***2.194(1)***	***FSR = 0.91***	***2.3756(7)***	***FSR = 0.99***
**Fe---P1**	*1.961(3)*		*2.149(1)*		*2.1876(6)*	
**Fe---P2**	*2.233(1)*		*2.169(1)*		*2.1922(7)*	
**Fe---P3**	*2.251(1)*		*2.156(1)*		*2.1807(6)*	
**Fe---X**	*2.4692(6)*	*X = Br1*	----------		----------	
**Al---N1**	*1.959(3)*		*1.963(3)*		*2.012(2)*	
**Al---N2**	*1.961(3)*		*1.950(3)*		*1.996(1)*	
**Al---X**	*2.460(1)*	*X = Br2*	----------		*2.063(2)*	*X = C*

Table S1. Summary of selected bond lengths obtained from X-ray structure analysis (FSR = Formal Shortness Ratio).

Table S2 provides a summary of the crystallographic data for the structures of **2a**, **2b**, **3a**, **3b**, **4a** and **5b**. Data were collected using Agilent Xcalibur 3 E (**2a**, **2b**, **3a** and **3b**) and Xcalibur PX Ultra A (**4a** and **5b**) diffractometers, and the structures were refined using the SHELXTL and SHELX-2013 program systems.[2,3] **CCDC 2127534 to 2127539**.

Table S2. Crystal Data, Data Collection and Refinement Parameters for the structures of **2a**, **2b**, **3a**, **3b**, **4a** and **5b**.

**Compound**	**2a**	**2b**	**3a**
**CCDC No.**	2127534	2127535	2127536
**formula**	$C_{32}H_{58}AlBr_2FeN_2P_3$	$C_{38}H_{70}AlBr_2FeN_2P_3$	$C_{32}H_{58}AlFeN_2P_3$
**solvent**	$0.5(C_4H_{10}O)$	—	—
**formula weight**	843.42	890.52	646.54
**colour, habit**	orange/brown blocks	orange blocks	red blocks
**temperature / K**	173	173	173
**crystal system**	triclinic	monoclinic	monoclinic
**space group**	*P*–1 (no. 2)	$P2_1/c$ (no. 14)	$P2_1/m$ (no. 11)
***a* / Å**	11.4832(5)	20.2610(7)	10.2064(5)
***b* / Å**	11.7899(4)	12.2143(4)	17.3862(10)
***c* / Å**	17.5580(5)	17.5106(7)	10.7541(5)
**α / deg**	104.304(3)	90	90
**β / deg**	96.530(3)	91.526(4)	102.353(5)
**γ / deg**	114.671(4)	90	90
**V / Å^3**	2028.68(14)	4331.9(3)	1864.14(17)
Z	2	4	2 [c]
**D_c / g cm^{-3}**	1.381	1.365	1.152
**radiation used**	Mo-Kα	Mo-Kα	Mo-Kα
**μ / mm^{-1}**	2.508	2.352	0.579
**no. of unique reflns**			
**measured (R_{int})**	8030 (0.0241)	14242 (0.0487)	5919 (0.0538)
**obs, $\|F_o\| > 4\sigma(\|F_o\|)$**	6498	10186	3795
**completeness (%)** [a]	98.7	99.8	98.3
**no. of variables**	436	452	253
**R_1(obs), wR_2(all)** [b]	0.0368, 0.0880	0.0364, 0.0897	0.0418, 0.1089

[a] Completeness to 0.84 Å resolution. [b] $R_1 = \Sigma||F_o| - |F_c||/\Sigma|F_o|$; $wR_2 = \{\Sigma[w(F_o^2 - F_c^2)^2] / \Sigma[w(F_o^2)^2]\}^{1/2}$; $w^{-1} = \sigma^2(F_o^2) + (aP)^2 + bP$. [c] The complex has crystallographic C_S symmetry. [d] There are two crystallographically independent complexes in the asymmetric unit.

Table S2. part 2

Compound	3b	4a	5b
**CCDC No.**	2127537	2127538	2127539
**formula**	$C_{38}H_{70}AlFeN_2P_3$	$C_{32}H_{58}AlFeN_2P_3$	$C_{43}H_{75}AlFeN_3P_3$
**solvent**	—	—	$0.5(C_4H_{10}O)$
**formula weight**	730.70	646.54	846.86
**colour, habit**	red blocks	yellow tablets	yellow/orange tablets
**temperature / K**	173	173	173
**crystal system**	triclinic	monoclinic	triclinic
**space group**	*P*–1 (no. 2)	$P2_1/n$ (no. 14)	*P*–1 (no. 2)
***a* / Å**	9.6825(10)	9.87436(15)	11.7820(5)
***b* / Å**	11.6198(13)	17.8866(3)	21.0271(8)
***c* / Å**	20.235(2)	21.1993(3)	21.7379(9)
**α / deg**	87.786(9)	90	115.188(4)
**β / deg**	86.746(9)	100.7324(16)	91.727(3)
**γ / deg**	66.769(10)	90	91.045(4)
**V / Å^3**	2088.3(4)	3678.69(10)	4868.2(4)
Z	2	4	4 [d]
**D_c / g cm^{-3}**	1.162	1.167	1.155
**radiation used**	Mo-Kα	Cu-Kα	Cu-Kα
**μ / mm^{-1}**	0.524	4.906	3.831
**no. of unique reflns**			
**measured (R_{int})**	12401 (0.0532)	7023 (0.0320)	18507 (0.0298)
**obs, $\|F_o\| > 4\sigma(\|F_o\|)$**	6892	5665	15768
**completeness (%)** [a]	98.0	98.7	98.2
**no. of variables**	427	380	1075
**R_1(obs), wR_2(all)** [b]	0.0468, 0.1060	0.0383, 0.1016	0.0368, 0.0966

The X-ray crystal structure of 2a: The O50-based included diethylether solvent molecule in the structure of **2a** was found to be disordered across a centre of symmetry, and two unique orientations were identified of *ca*. 27 and 23% occupancy (with two further orientations of the same occupancies being generated by operation of the inversion centre). The geometries of the two unique orientations were optimised, the thermal parameters of adjacent atoms were restrained to be similar, and all of the atoms of both unique orientations were refined isotropically. The two Al–H–Fe bridging hydrogen atoms were located from Δ*F* maps and refined freely.

The X-ray crystal structure of 2b: The crystal of **2b** that was studied was found to be a two component twin in a *ca*. 54:46 ratio, with the two lattices related by the approximate twin law [1.00 0.00 0.05 0.00 –1.00 0.00 0.00 0.00 –1.00]. The two Al–H–Fe bridging hydrogen atoms were located from Δ*F* maps and refined freely.

The X-ray crystal structure of 3a: The crystal of **3a** that was studied was found to be a two component twin in a *ca*. 69:31 ratio, with the two lattices related by the approximate twin law [–0.07 0.00 0.93 0.01 –1.00 0.01 1.07 0.00 0.07]. The structure was found to sit across a mirror plane that passes through C2, Al1 and Fe1, and bisects the N1··N1A vector. The $Fe(PMe_3)_3$ group was found to be disordered about this mirror plane and this was modelled by using one complete, 50% occupancy orientation (the iron atom being common to both), with a further orientation being generated by operation of the mirror plane. The geometry of the unique orientation was optimised, and all of the non-hydrogen atoms were refined anisotropically. The unique Al–H–Fe bridging hydrogen atom was located from a Δ*F* map and refined freely.

The X-ray crystal structure of 3b: The crystal of **3b** that was studied was found to be a two component twin in a *ca*. 69:31 ratio, with the two lattices related by the approximate twin law [–1.00 0.00 0.05 0.00 –1.00 0.05 0.00 0.00 1.00]. The presumed two Al–H–Fe bridging hydrogen atoms could not be reliably located, and so the atom list for the asymmetric unit is low by two hydrogen atoms, and that for the unit cell as a whole is low by four hydrogen atoms.

The X-ray crystal structure of 4a: The three Al–H–Fe bridging hydrogen atoms in the structure of **4a** were located from Δ*F* maps and refined freely. Though ultimately handled conventionally, the CH_2 hydrogen atoms on C21 were first located from Δ*F* maps and found to be in almost exactly the same positions as the idealised model subsequently placed them.

The X-ray crystal structure of 5b: The structure of **5b** was found to contain two crystallographically independent complexes (5**b-A** and **5b-B**) in the asymmetric unit. The C12-based *iso*-propyl group in complex **5b-B** was found to be disordered. Two orientations were identified of *ca*. 66 and 34% occupancy, their geometries were optimised, the thermal parameters of adjacent atoms were restrained to be similar, and only the non-hydrogen atoms of the major occupancy orientation were refined anisotropically (those of the minor occupancy orientation were refined isotropically). The O60- and O70-based included diethylether solvent molecules were both found to be disordered across centres of symmetry, and in each case two unique orientations were identified of *ca*. 31:19 and 25:25% occupancy (with two further orientations of the same occupancies being generated by operation of the associated inversion centres). The geometries of the two pairs of unique orientations were optimised, the thermal parameters of adjacent atoms were restrained to be similar, and all of the atoms of the four unique orientations were refined isotropically. The six unique Al–H–Fe bridging hydrogen atoms (three per independent complex) were located from Δ*F* maps and refined freely.

4. Kinetic Experiments

Eyring Analysis: In a glovebox, a solution of **3a** (29.1 mg, 0.045 mmol) in toluene-d_8 (2.0 mL) was equally split into four J. Young NMR tubes. Capillary inserts containing PPh_3 in toluene-d_8 as standard were added to each NMR tube. The J. Young tubes were sealed, taken out of the glovebox, and heated in isothermal baths, each at the specified temperature (60, 80, 90, 100 °C). The reactions were interrupted from time to time by taking the NMR tubes out of the heating baths and ^{1}H as well as ^{31}P NMR spectra were recorded to monitor the progress at each temperature.

Four (first order) rate constants were obtained (Figure S1) and plotting the ln(k/T) against 1/T (Figure S2) allowed for the calculation of the thermodynamic parameters using the Eyring equation. The activation parameters for the reaction were found to be **ΔH‡ = 23.4 ± 0.5 kcal mol^{-1}**, **ΔS‡ = 12.5 ± 1.3 cal mol^{-1} K^{-1}** and an associated **ΔG‡(298) = 27.1 ± 0.8 kcal mol^{-1}**.

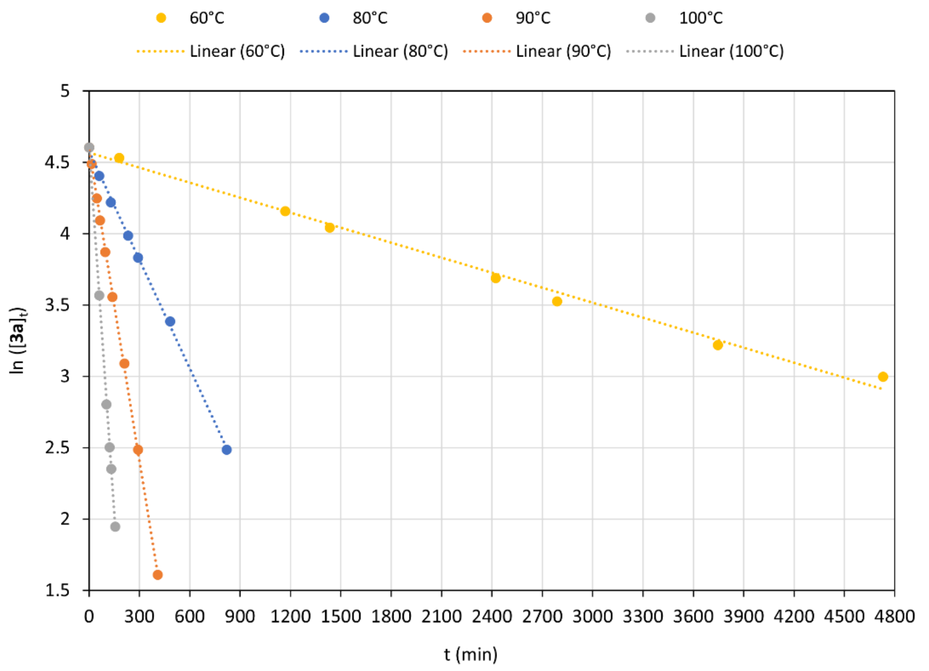

Figure S1. Kinetic data for the reaction of **3a** to **4a** in a temperature range of 60-100 °C.

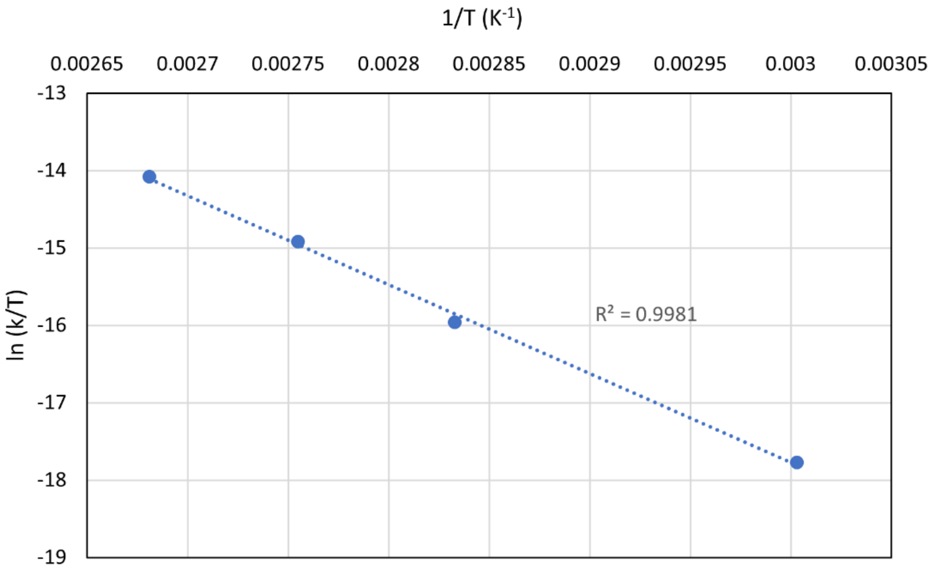

Figure S2. Eyring plot for the reaction of **3a** to **4a** in the temperature range of 60-100 °C.

KIE measurements: The H/D-KIE for the reaction of **3a** to **5a** was determined by comparison of the relative first order rate constants (k_H/k_D) obtained for pyridine (k_H) and pyridine-d_5 (k_D) in separate runs. In a glovebox, 2.0 mL of a 0.008 M solution of **3a** in C_6D_6 was prepared and equally split into two NMR tubes containing capillary inserts with a PPh_3/ C_6D_6 standard. The NMR tubes were sealed with a septum screw cap and taken to the NMR spectrometer. For each run 10 equiv. of pyridine or pyridine-d_5 (200 µL of a 0.4 M stock solution in C_6D_6) were injected through the septum using a syringe (t = 0), the NMR tube immediately inserted into the magnet and the reaction monitored by ^{31}P NMR. The measurement of k_H and k_D were conducted twice (Figure S3) giving a KIE of **14.0 ± 0.2**.

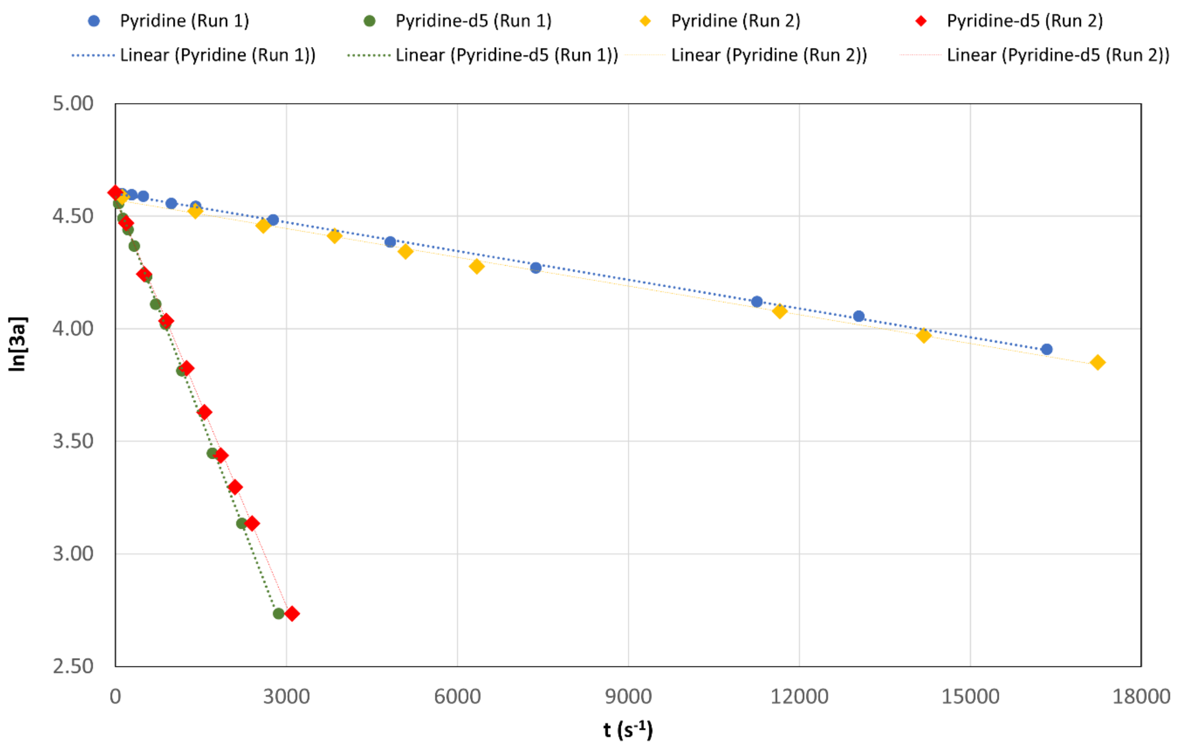

Figure S3. Kinetic data for the determination of the KIE in the reaction of **3a** to **5a** at 297 K.

5. Computational Methods

DFT calculations were run using Gaussian 09 (Revision D.01).[4] NBO analysis was performed using the NBO 6.0 version program.[5] QTAIM analysis was conducted with the AIMAll package.[6]

Al and Fe centres were described with Stuttgart SDDAll ECP and associated basis sets, and the 6-31G** basis sets were used for all other atoms. Geometry optimisation calculations were performed without symmetry constraints. Frequency analyses for all stationary points were performed using the enhanced criteria to confirm the nature of the structures as either minima (no imaginary frequency) or transition states (only one imaginary frequency). Solvent corrections were applied using the polarizable continuum model (PCM). Dispersion corrections were applied using Grimme's D3 correction.

Ground state structures were optimised at a ωB97x // 6-31G** / SSDAll level of theory (see Table S2 for the functional screening). These structures were used as input for NBO, QTAIM and ETS-NOCV calculations. Free energy profiles were calculated at a B3PW91 // 6-31G** / SSDAll level of theory including solvent and dispersion corrections directly in the optimisations (see Table S3 for the functional screening). All calculation were carried out on the singlet energy surface. The triplet energy surface was found to be about 10 kcal/mol higher in energy for both intermediate **1-A** ($\Delta G_{singlet\text{-}triplet}$ = 10.8 kcal/mol) and the transition state **1-TS$_{AB}$** ($\Delta G^{‡}_{singlet\text{-}triplet}$ = 10.6 kcal/mol).

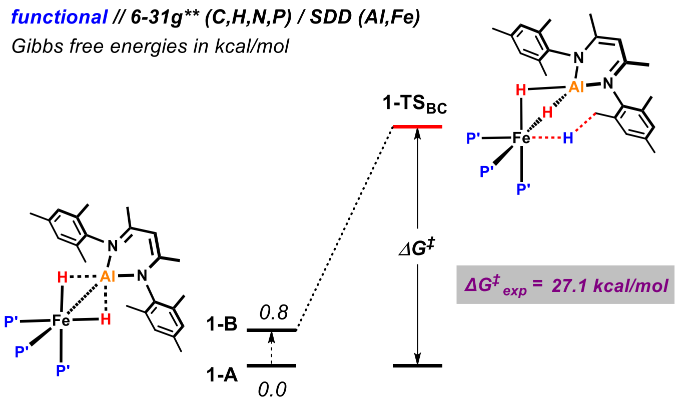

functional	*ΔG‡*	*ΔG‡ (cor.)* [a]	*ΔG‡ (cor.)* [b]
**wB97X**	**36.9**	**---**	**---**
**wB97X-D** [c]	**34.6**	**34.3**	**35.4**
**M06L**	**29.1**	**28.8**	**29.1**
**B3PW91**	**33.7**	**28.9**	**27.9**

a) solvation (PCM, Toluene) and dispersion (D3 except wB97X-D) included as single point correction; b) solvation and dispersion included in the optimisation; c) dispersion already included in the functional

Table S3. Functional testing using the intramolecular C–H activation of **3a** as benchmark reaction.

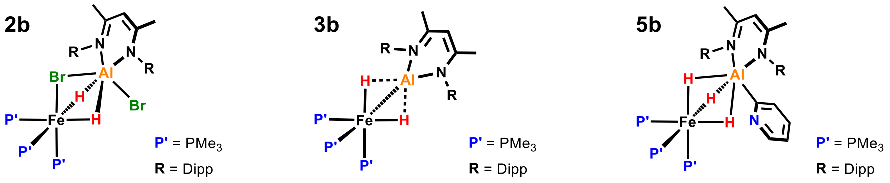

	**2b** Fe---Al (Å)		**3b** Fe---Al (Å)		**5b** Fe---Al (Å)	
**exp.**	*2.459(1)*	*FSR = 1.02*	*2.194(1)*	*FSR = 0.91*	*2.3756(7)*	*FSR = 0.98*
**wB97-X**	*2.465*		*2.229*		*2.366*	
**M06L**	*2.475*		*2.248*		*2.383*	
**B3PW91**	*2.479*		*2.250*		*2.380*	
**wB97-XD**	*2.441*		*2.208*		*2.345*	
**M06L / D3**	*2.473*		*2.246*		*2.382*	
**B3PW91 / D3**	*2.421*		*2.210*		*2.335*	

Table S4. Functional testing on the ground state structures of **2b**, **3b** and **5b** using the Fe---Al distance determined by X-ray diffraction as benchmark.

ETS-NOCV calculations (Table S5) were performed in the Orca 4.2.1 quantum chemistry software package with optimised geometries obtained at a ωB97x // 6-31G** / SSDAll level of theory.[7,8] The calculations were run using the ωB97x functional with the def2-tzvpp basis set. Calculations were performed with the resolution of identity approximation for the Coulomb integrals, and chain of spheres approximation for the exchange integrals (RIJCOSX) with the def2/j auxiliary basis set.[9]

	ΔE_{orb}	$\Delta\rho_1$	$\Delta\rho_2$	$\Delta\rho_3$
**3a**	***-118.1***	***-74.7*** *(63 %)*	***-19.0*** *(16 %)*	***-13.7*** *(12 %)*
**3b**	***-117.1***	***-74.7*** *(64 %)*	***-16.6*** *(14 %)*	***-14.2*** *(12 %)*
**3c**	***-117.1***	***-78.7*** *(67 %)*	***-17.8*** *(15 %)*	***-14.3*** *(12 %)*

*deformation density plots of **3c**:*

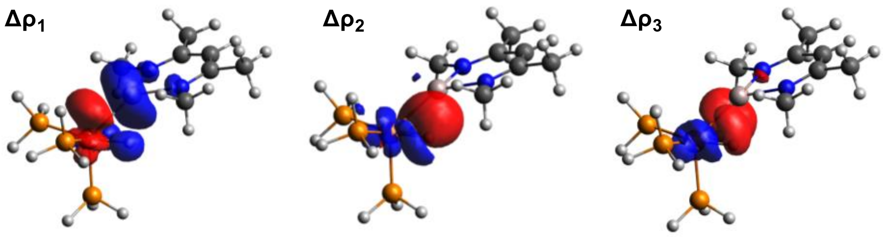

Table S5. ETS-NOCV deformation densities of the main contributions to the bonding between the Fe {P'$_3$Fe(H)$_2$} and the Al {^{R}*BDI*Al:} fragment. Charge flow from blue to red. All energies are given in kcal/mol.

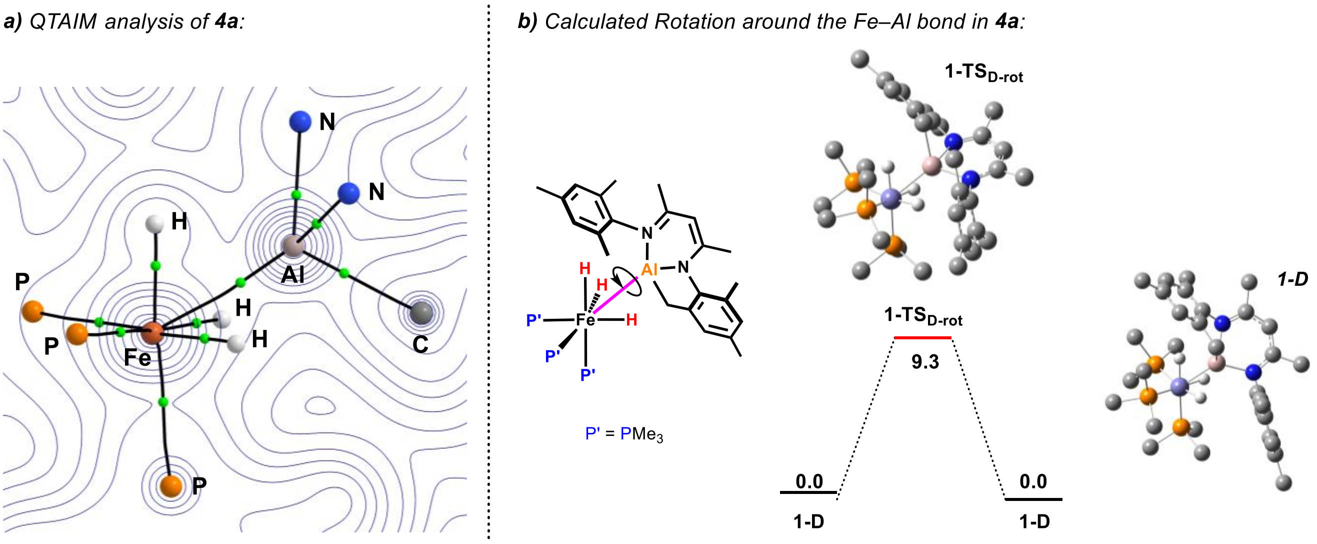

Figure S4. a) QTAIM analysis of complexes **4a** showing bond critical points (green) and paths (black lines). b) Calculated barrier for the rotation around the Fe–Al bond in 4a (B3PW91 // 6-31G** (non-metals) / SSDAll (Fe,Al)). Gibbs free energies are given in kcal/mol.

	3c		3a		3b
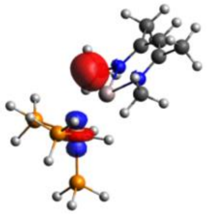	**LUMO+2**<br>+2.004 eV	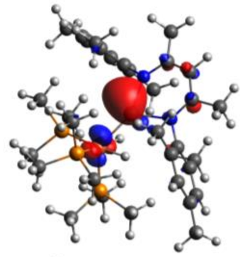	**LUMO+6**<br>+2.549 eV	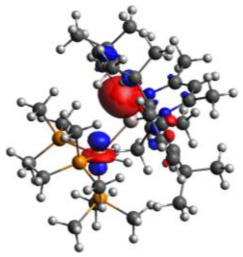	**LUMO+6**<br>+2.461 eV
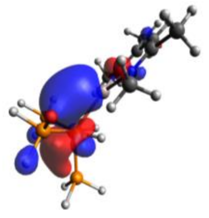	**HOMO**<br>-6.576 eV	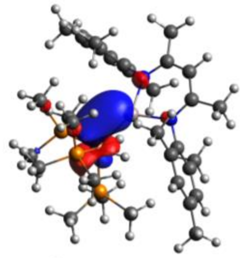	**HOMO**<br>-5.774 eV	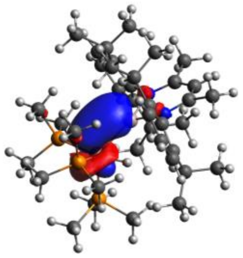	**HOMO**<br>-5.846 eV
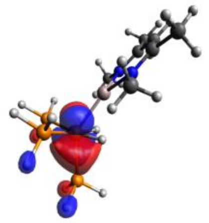	**HOMO-1**<br>-7.487 eV	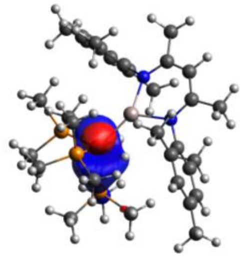	**HOMO-1**<br>-6.861 eV	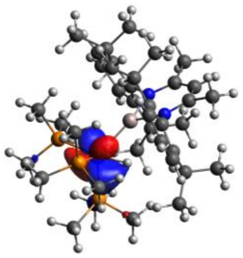	**HOMO-1**<br>-6.988 eV
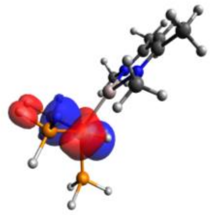	**HOMO-2**<br>-7.653 eV	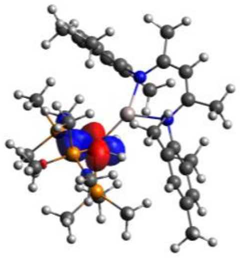	**HOMO-2**<br>-7.090 eV	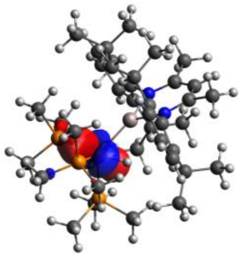	**HOMO-2**<br>-7.154 eV
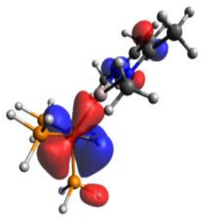	**HOMO-3**<br>-7.700 eV	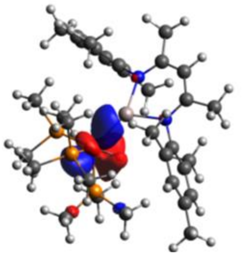	**HOMO-3**<br>-7.123 eV	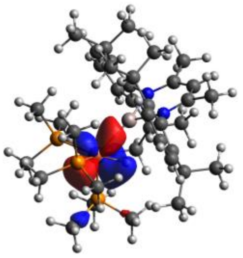	**HOMO-3**<br>-7.274 eV

Table S6. Frontier orbitals of the simplified model complex **3c** in comparison to those of complexes **3a** and **3b**.

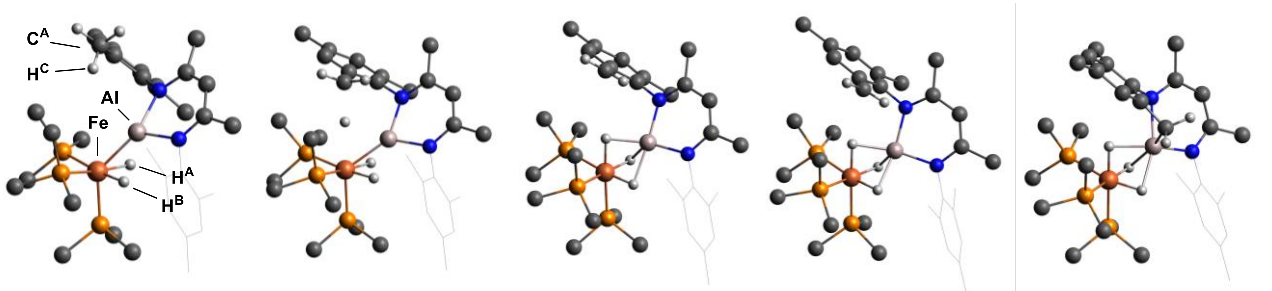

	1-B	$1\text{-}TS_{BC}$	1-C	$1\text{-}TS_{CD}$	1-D
**Fe---Al (Å)**	*2.207*	*2.206*	*2.221*	*2.224*	*2.322*
$\mathbf{WI_{Fe\text{-}Al}}$	*0.51*	*0.25*	*0.18*	*0.18*	*0.15*
NPA charges:					
**Fe**	*-1.03*	*-0.89*	*-0.83*	*-0.82*	*-0.86*
**Al**	*1.32*	*1.61*	*1.74*	*1.73*	*1.70*
$\mathbf{H^A}$	*-0.14*	*-0.17*	*-0.18*	*-0.19*	*-0.18*
$\mathbf{H^B}$	*-0.22*	*-0.21*	*-0.21*	*-0.22*	*-0.16*
$\mathbf{H^C}$	*0.26*	*-0.21*	*-0.16*	*-0.14*	*-0.16*
$\mathbf{C^A}$	*-0.71*	*-0.82*	*-0.74*	*-0.72*	*-1.07*

Table S7. Length and Wiberg bond indices of the Fe–Al bond as well as selected NPA charges along the reaction coordinate of the intramolecular C–H activation of the mesityl CH_3 group.

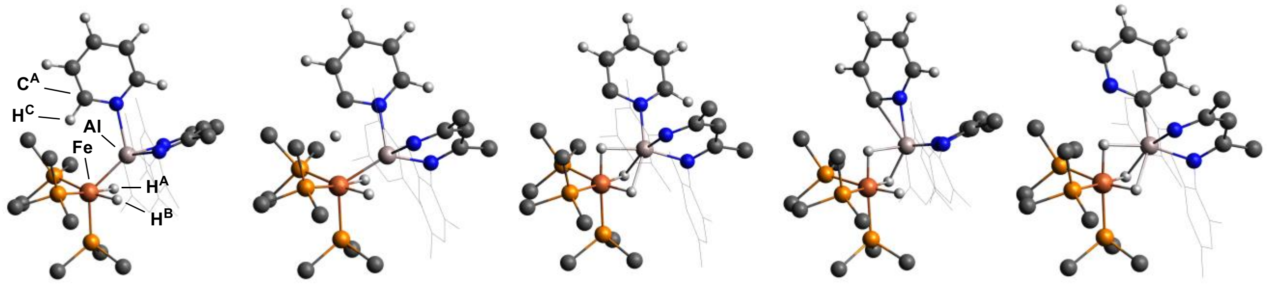

	2-A	$2\text{-}TS_{AB}$	2-B	$2\text{-}TS_{BC}$	1-C
**Fe---Al (Å)**	*2.246*	*2.269*	*2.298*	*2.355*	*2.328*
$\mathbf{WI_{Fe\text{-}Al}}$	*0.43*	*0.25*	*0.15*	*0.14*	*0.15*
NPA charges:					
**Fe**	*-1.13*	*-1.00*	*-0.85*	*-0.88*	*-0.85*
**Al**	*1.48*	*1.70*	*1.80*	*1.78*	*1.71*
$\mathbf{H^A}$	*-0.17*	*-0.21*	*-0.19*	*-0.13*	*-0.18*
$\mathbf{H^B}$	*-0.15*	*-0.20*	*-0.20*	*-0.19*	*-0.19*
$\mathbf{H^C}$	*0.27*	*0.11*	*-0.16*	*-0.20*	*-0.17*
$\mathbf{C^A}$	*0.05*	*-0.10*	*-0.12*	*-0.13*	*-0.33*

Table S8. Length and Wiberg bond indices of the Fe–Al bond as well as selected NPA charges along the reaction coordinate of the intermolecular *ortho* C–H activation of pyridine.

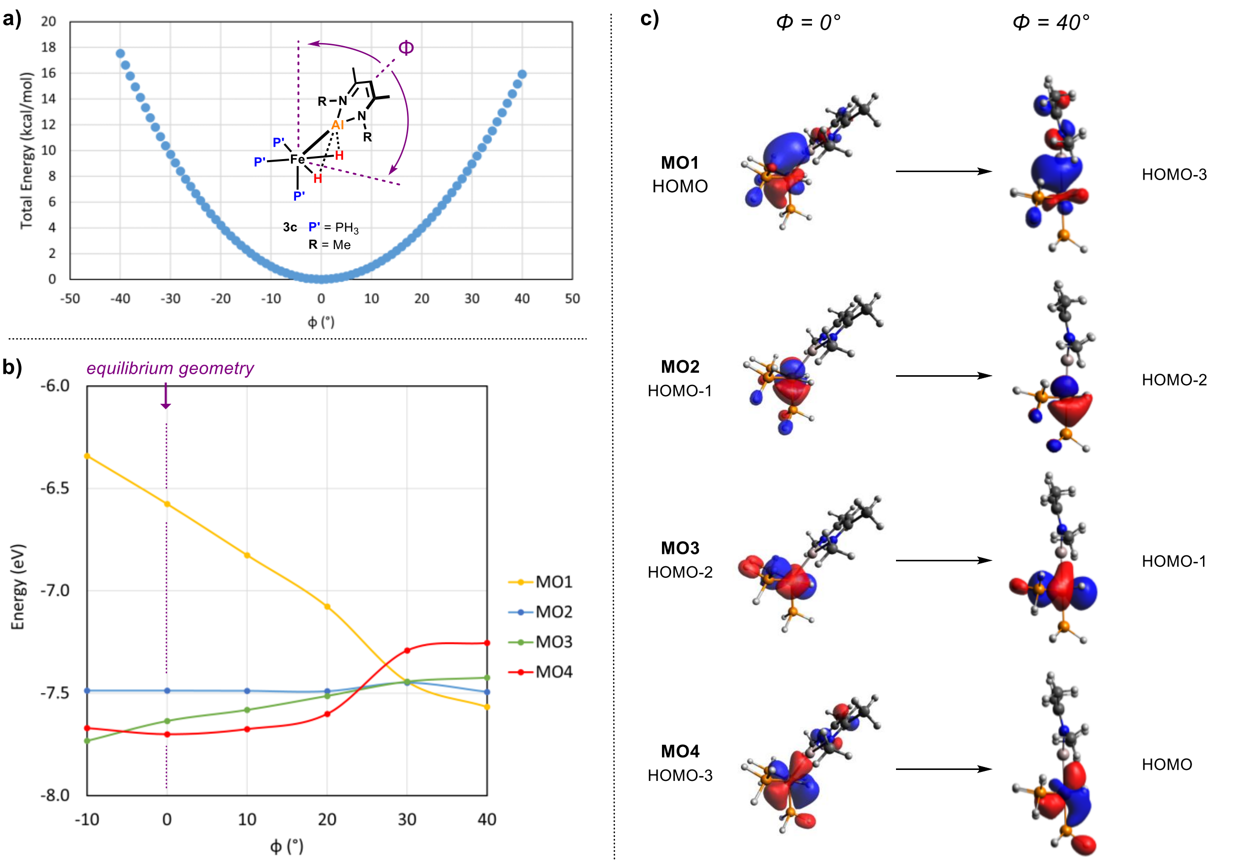

Figure S5. a) Energy profile for the relaxed scan of the Al-Fe-P_{ax} angle in **3c**. b) energies of the occupied frontier orbitals in **3c** as function of the Al-Fe-P_{ax} bending angle. c) Orbital isosurfaces (isovalue = 0.05) at 0° (equilibrium structure) and 40° (metalloligand in the axial position) bending angles.

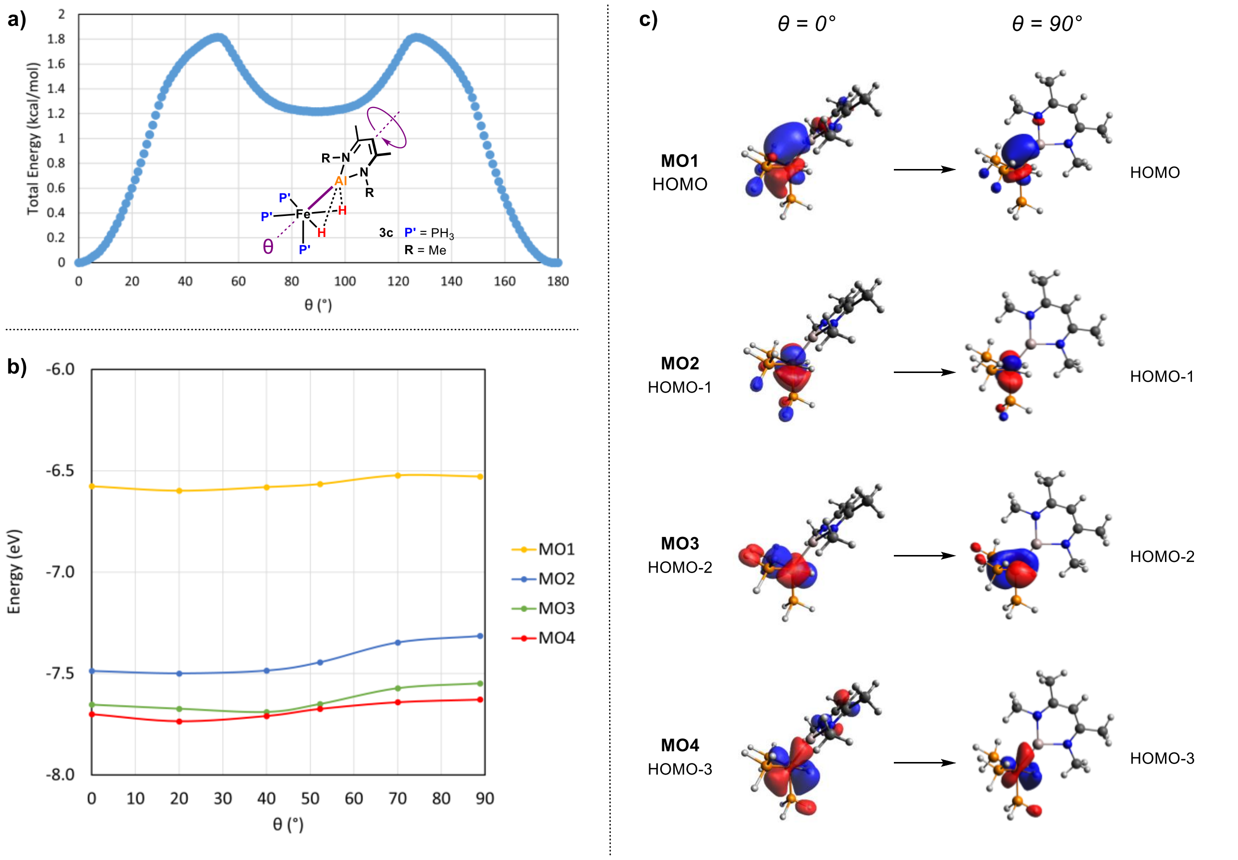

Figure S6. a) Energy profile for the relaxed scan of the rotation of the metalloligand around the Fe–Al bond in **3c**. b) energies of the occupied frontier orbitals in **3c** as function of the (dihedral) rotation angle. c) Orbital isosurfaces (isovalue = 0.05) at 0° (equilibrium structure) and 90° (second rotamer) rotation angles.

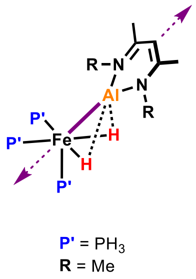

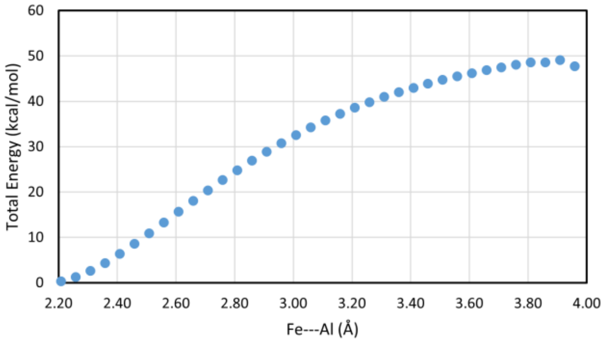

LUMO+2

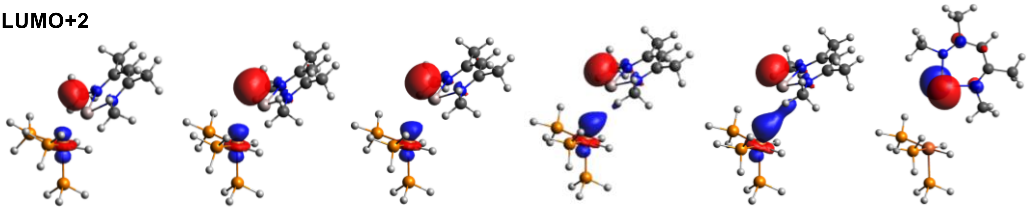

HOMO

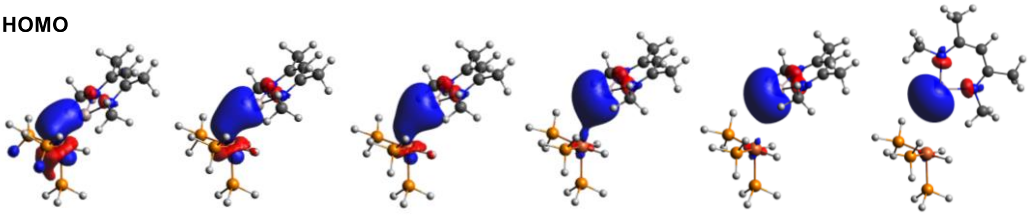

Fe---Al (Å)					
2.209	*2.709*	*2.959*	*3.459*	*3.709*	*3.959*
C_{Fe} / C_{Al}					
-1.01 / 1.34	*-0.80 / 1.16*	*-0.65 / 1.04*	*-0.39 / 0.84*	*-0.31 / 0.78*	*-0.26 / 0.74*
$\Delta C_{Fe/Al}$					
2.35	*1.96*	*1.69*	*1.23*	*1.09*	*1.00*

Figure S7. Relaxed scan of the potential energy surface regarding the Fe–Al bond distance in **3c** showing isosurfaces of the frontier orbitals and NPA charges along the redundant coordinate.

6. NMR Spectra of the Isolated Compounds

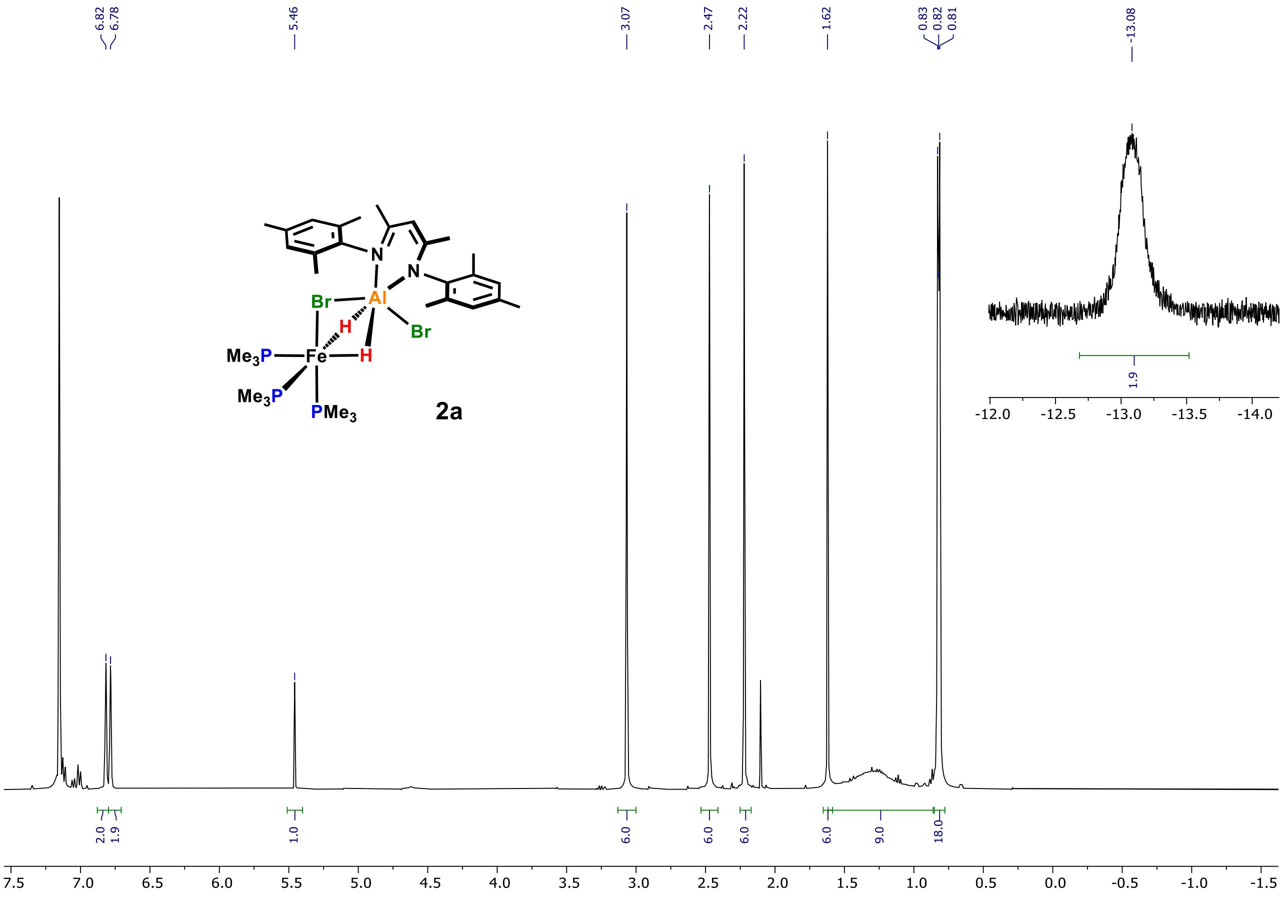

Figure S8. ^{1}H NMR of **2a** (400 MHz, C_6D_6, 298 K).

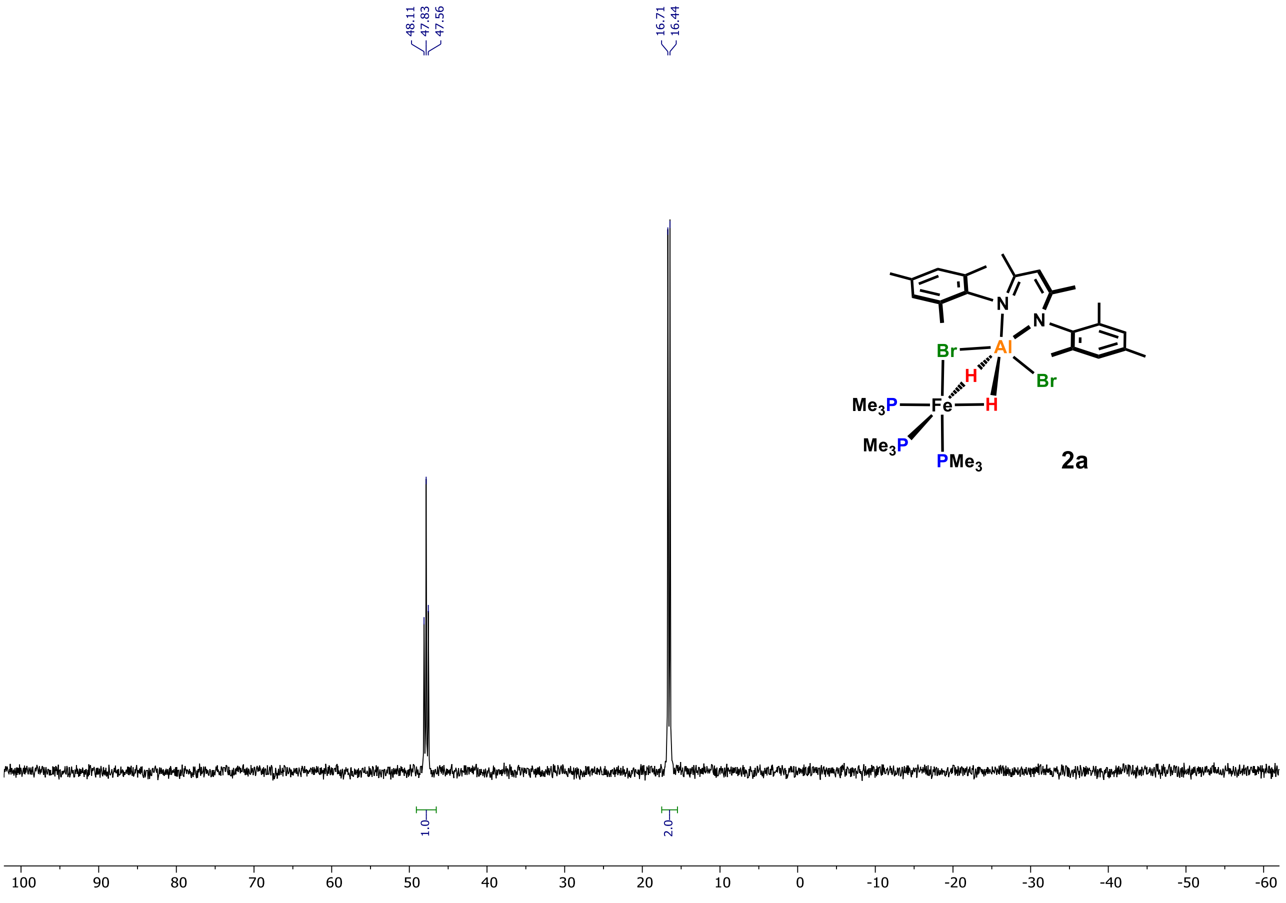

Figure S9. $^{31}P\{^{1}H\}$ NMR of **2a** (162 MHz, C_6D_6, 298 K).

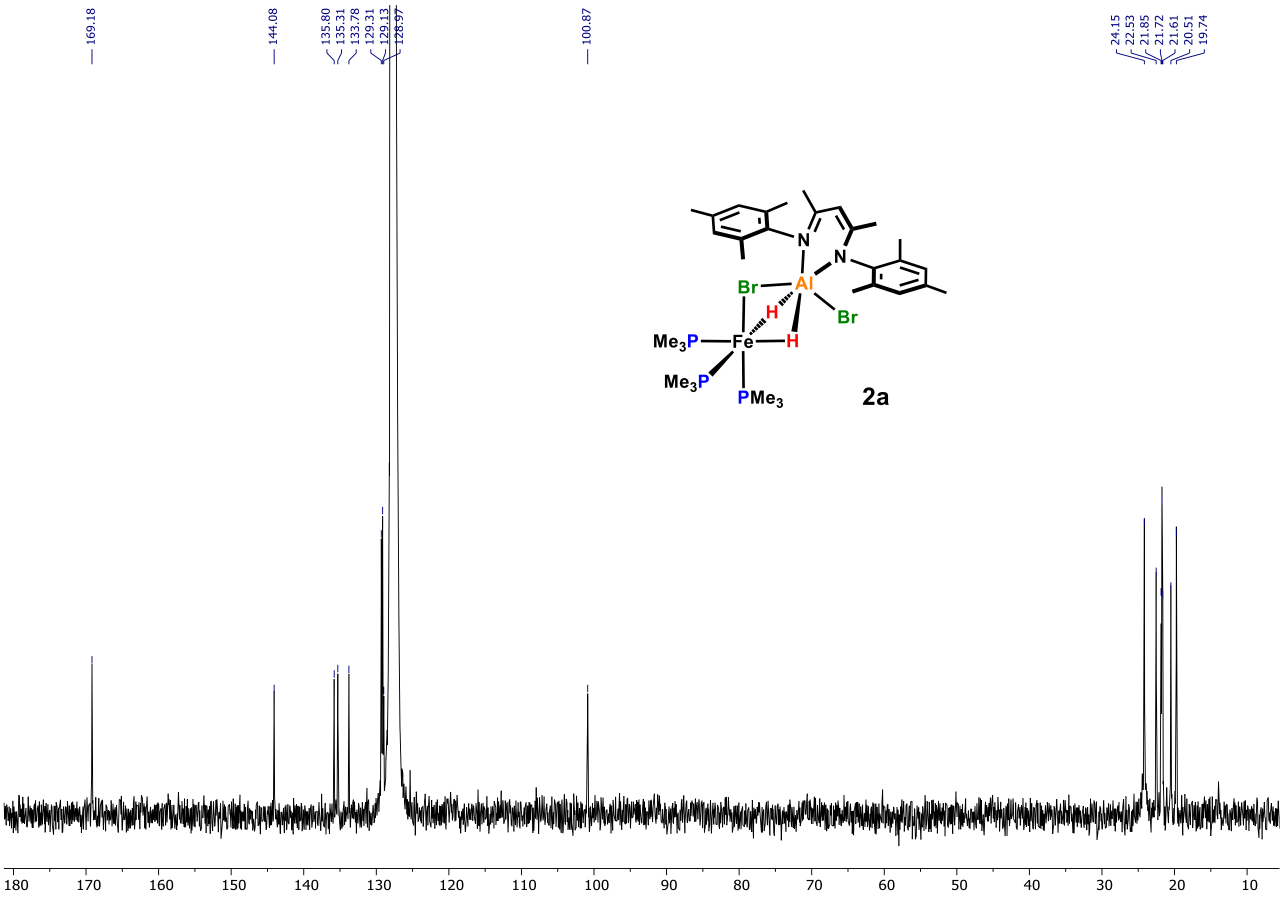

Figure S10. $^{13}C\{^{1}H\}$ NMR of **2a** (101 MHz, C_6D_6, 298 K).

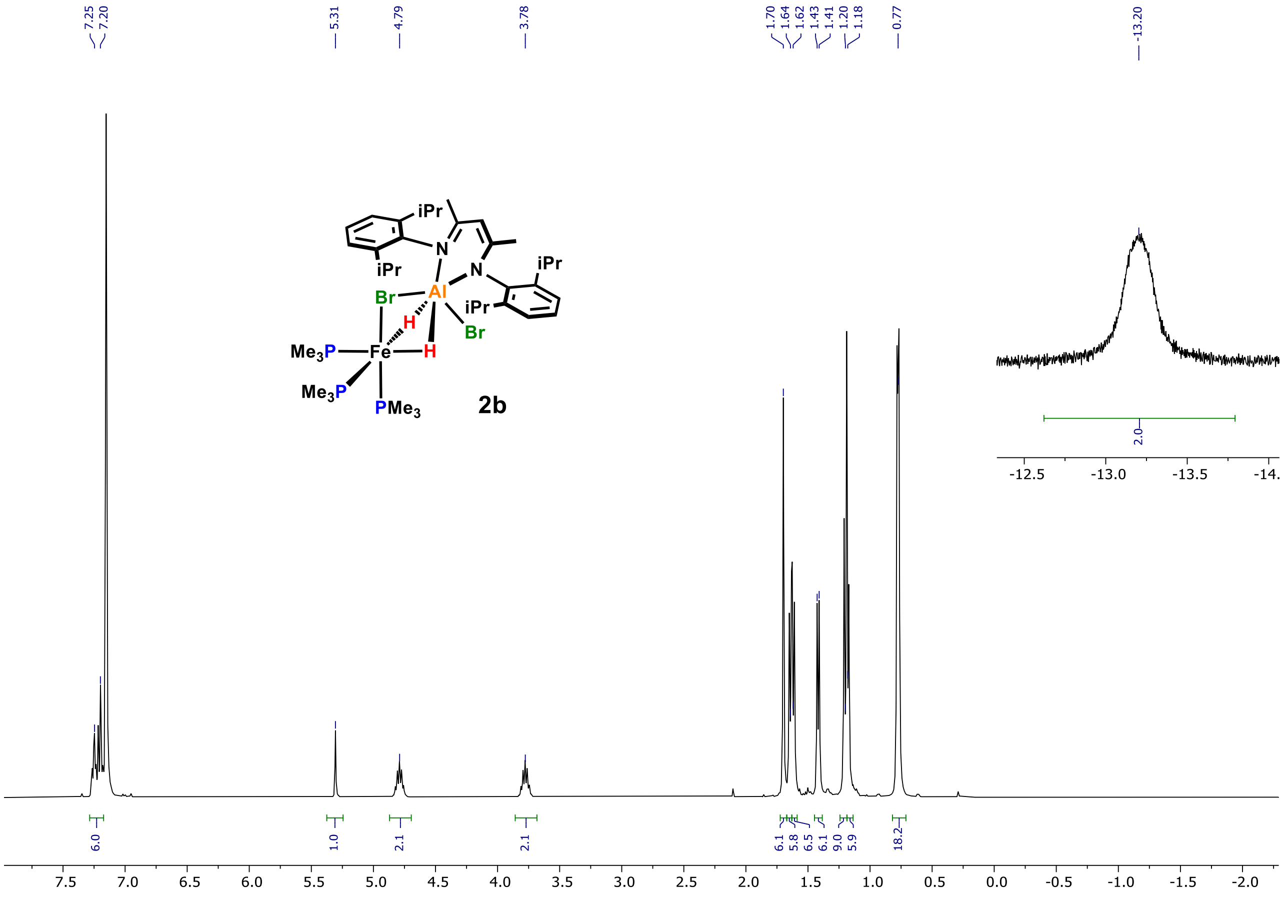

Figure S11. ^{1}H NMR of **2b** (400 MHz, C_6D_6, 298 K).

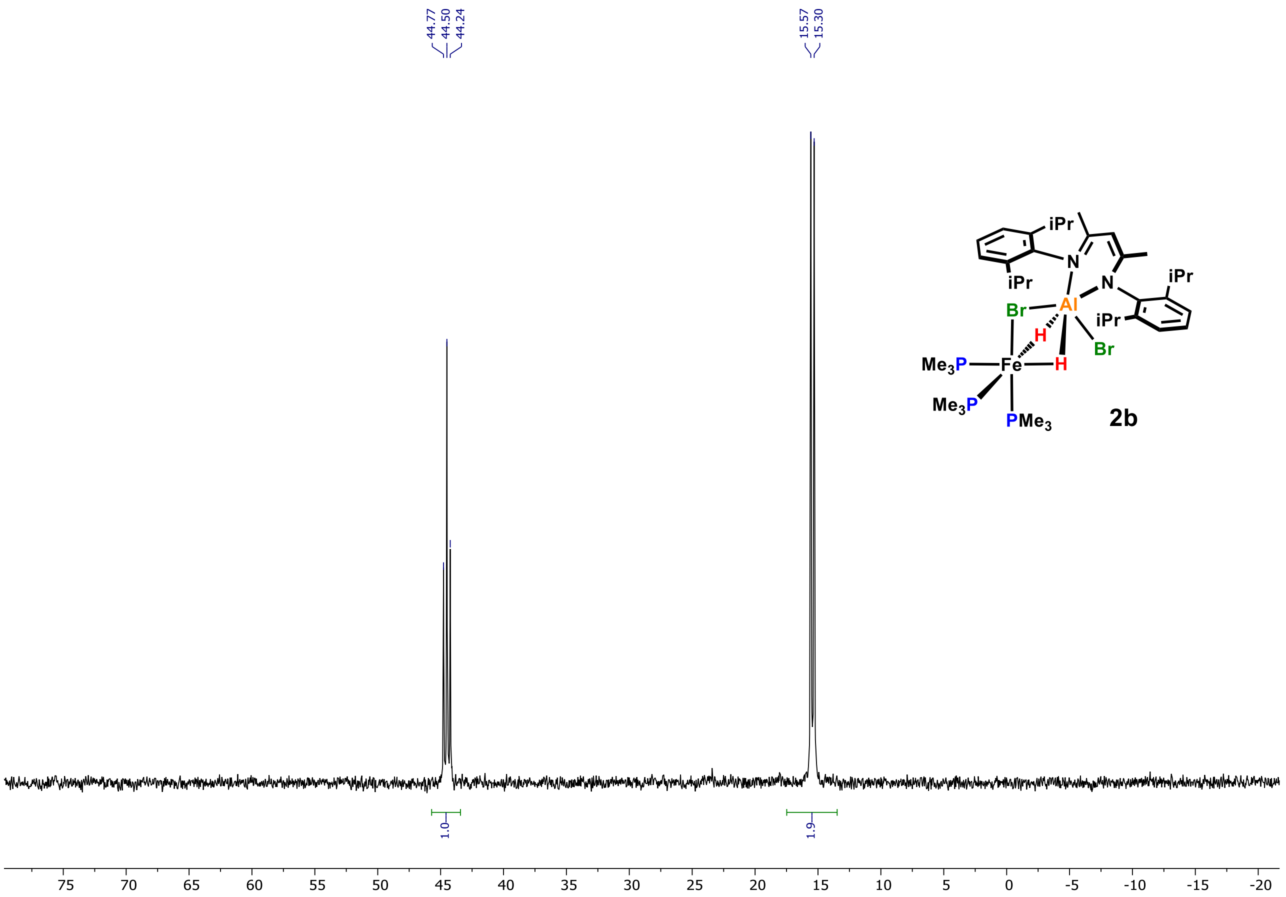

Figure 12. $^{13}C\{^{1}H\}$ NMR of **2b** (101 MHz, C_6D_6, 298 K).

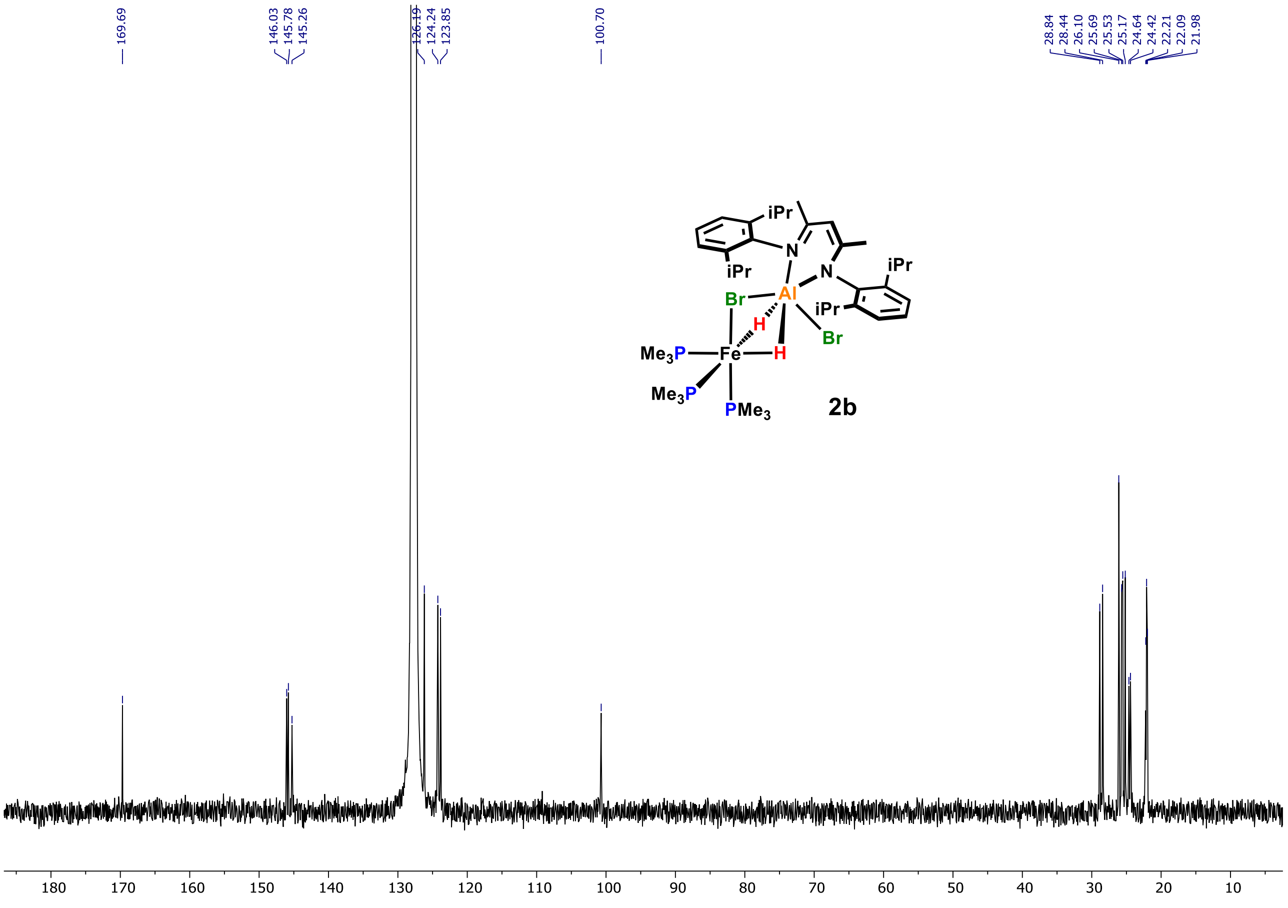

Figure S13. $^{13}C\{^{1}H\}$ NMR of **2b** (101 MHz, C_6D_6, 298 K).

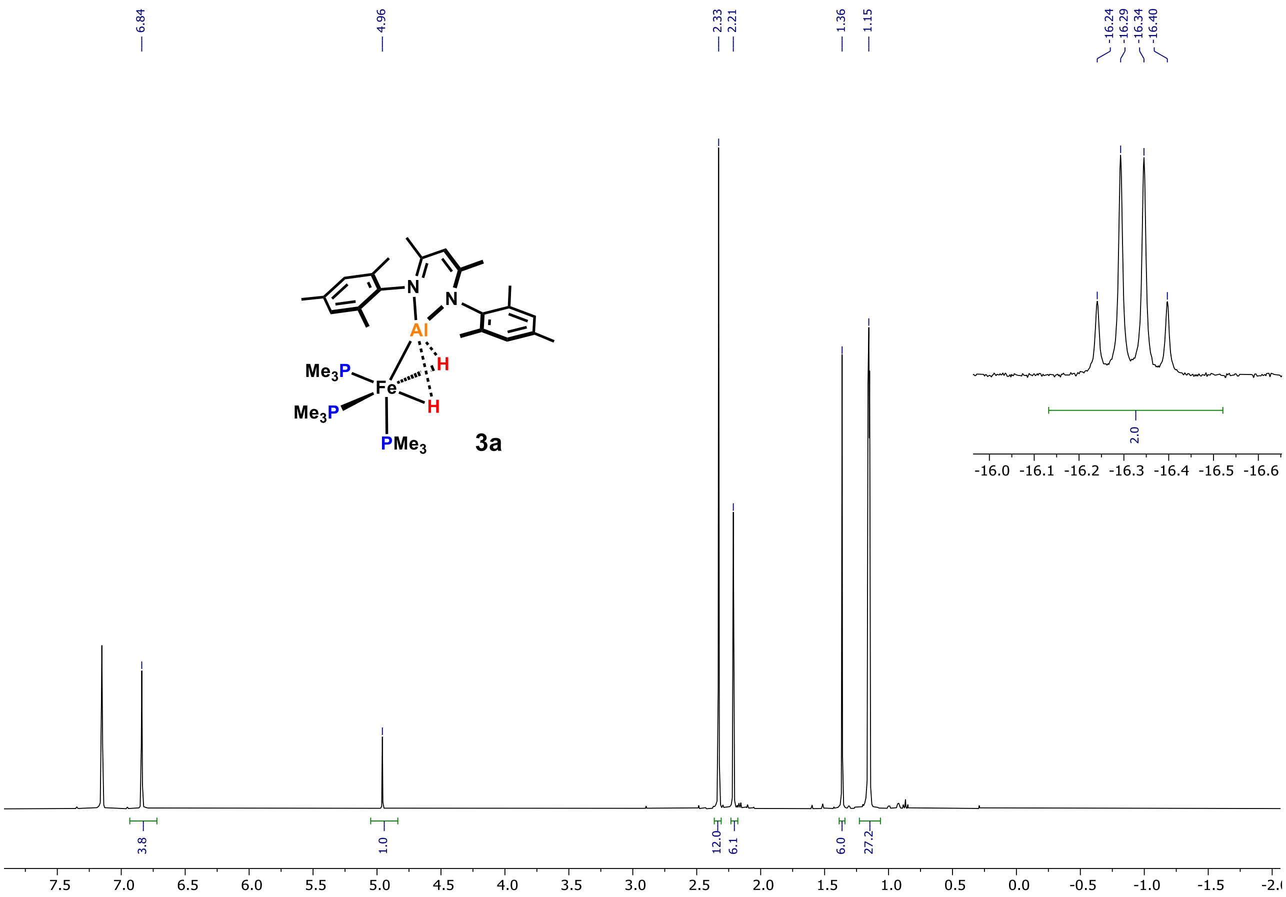

Figure S14. ^{1}H NMR of **3a** (400 MHz, C_6D_6, 298 K).

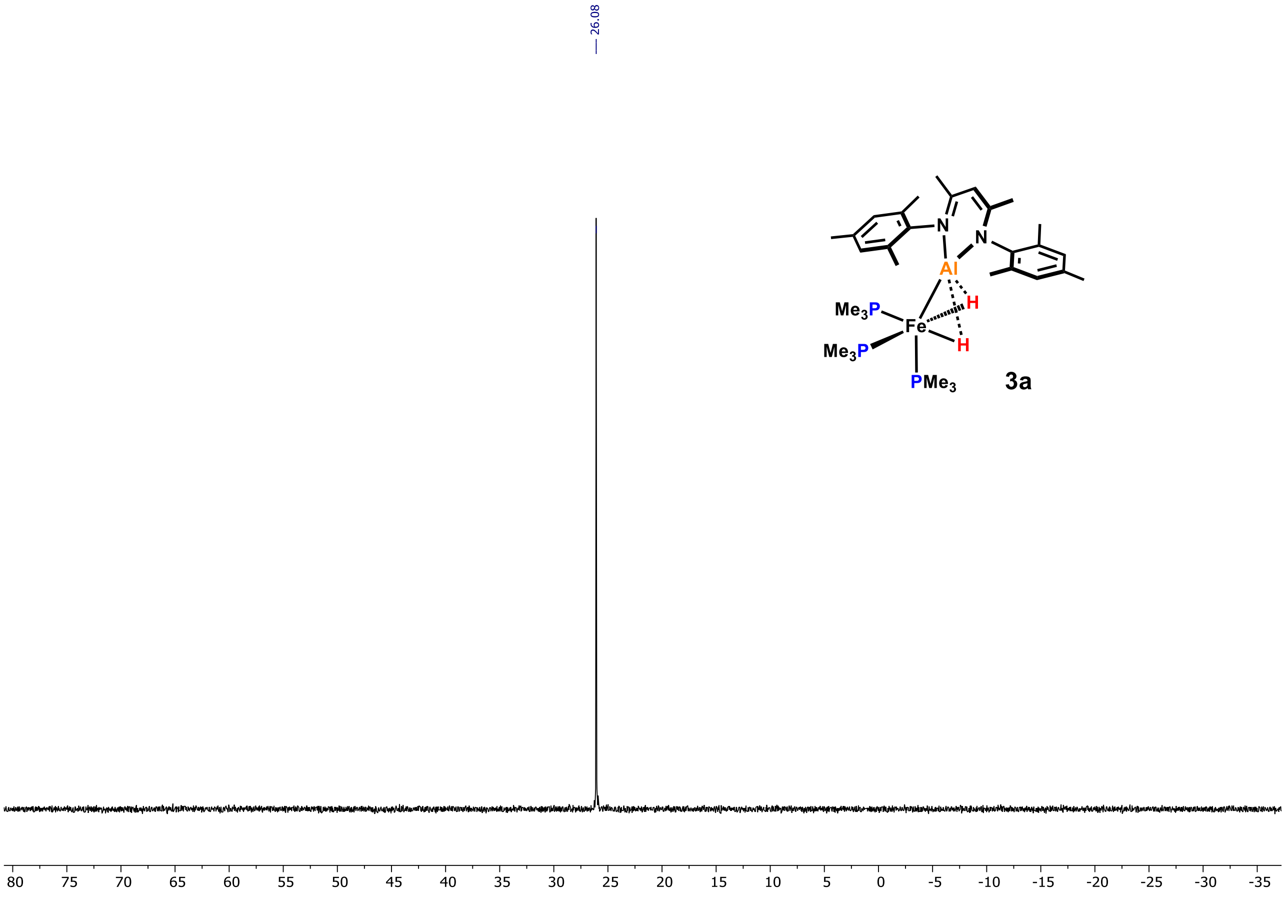

Figure S15. $^{31}P\{^{1}H\}$ NMR of **3a** (162 MHz, C_6D_6, 298 K).

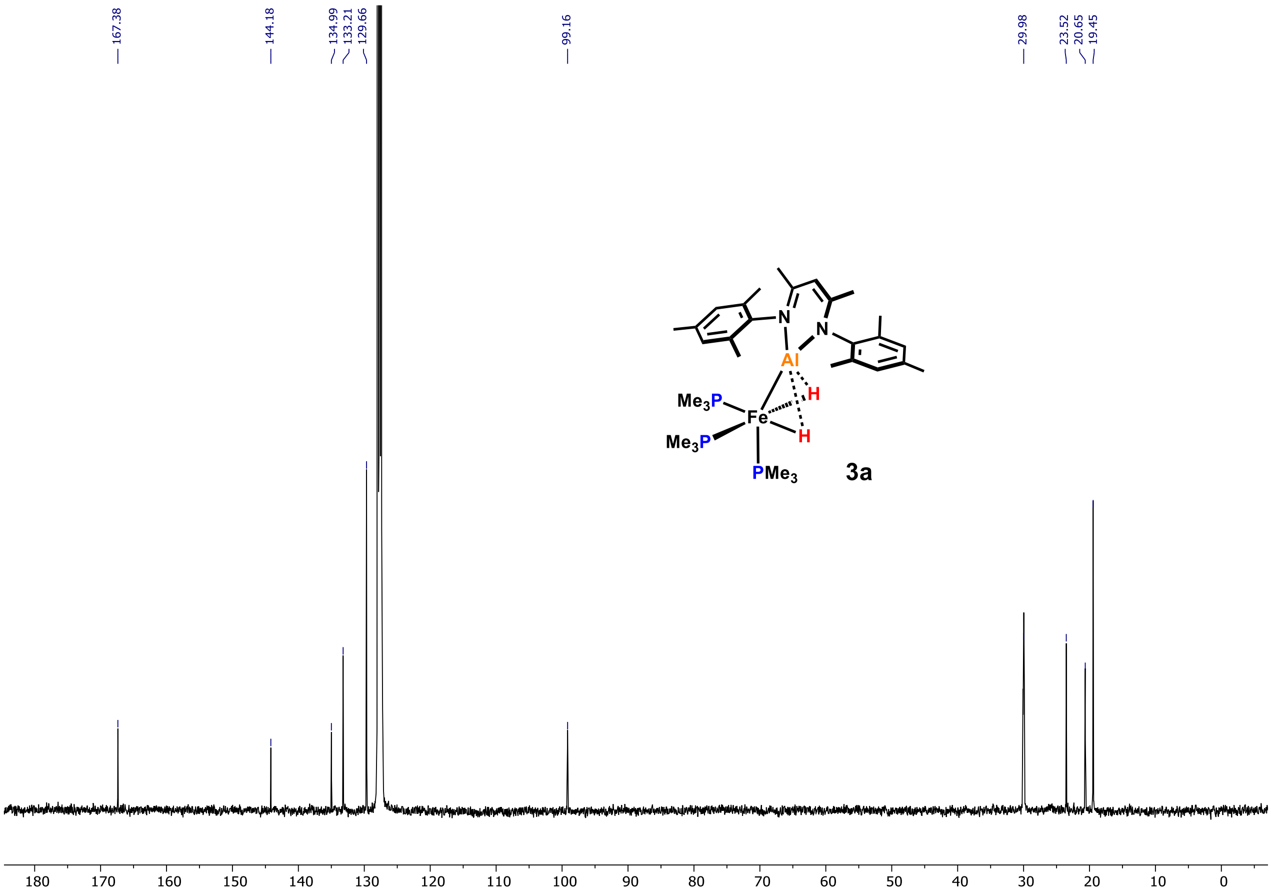

Figure S16. $^{13}C\{^{1}H\}$ NMR of **3a** (101 MHz, C_6D_6, 298 K).

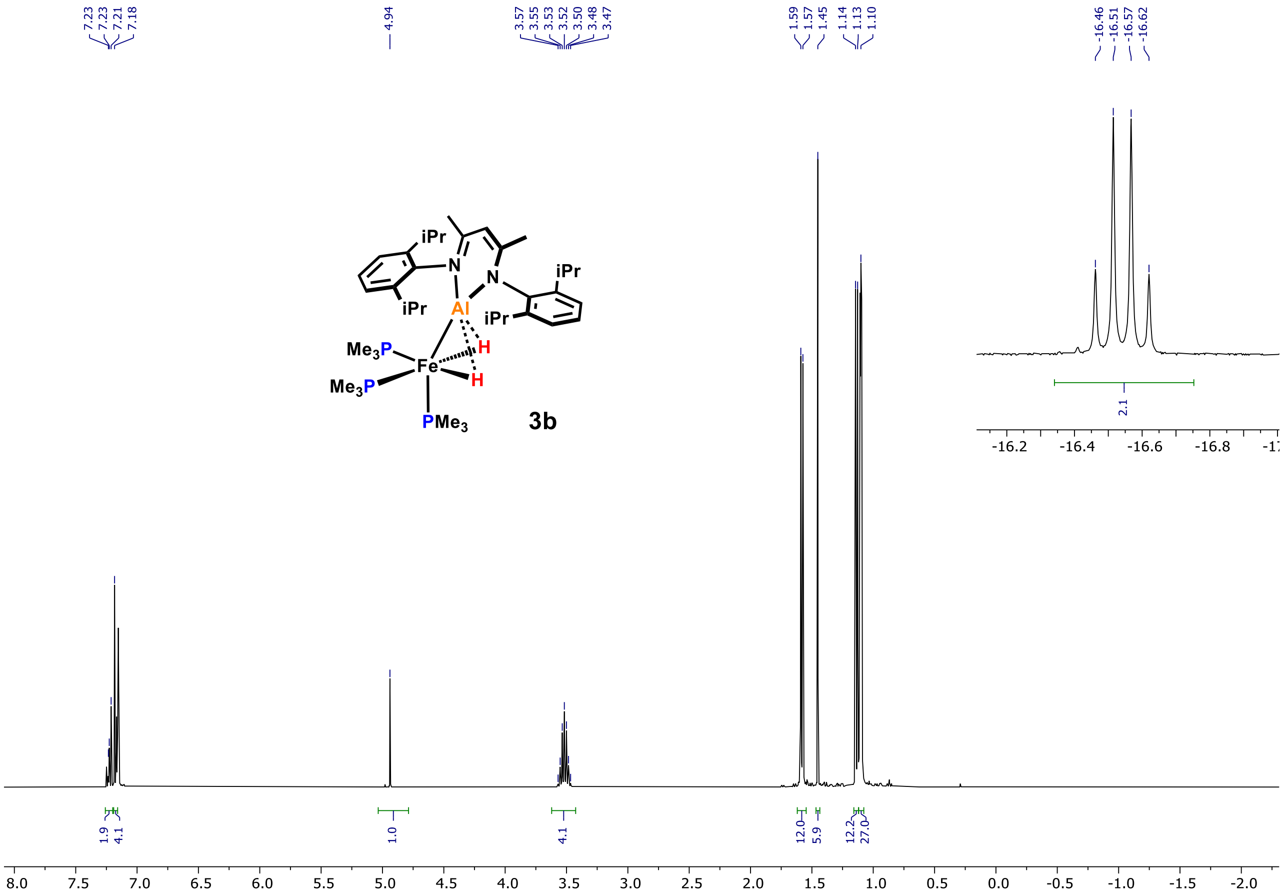

Figure S17. ^{1}H NMR of **3a** (400 MHz, C_6D_6, 298 K).

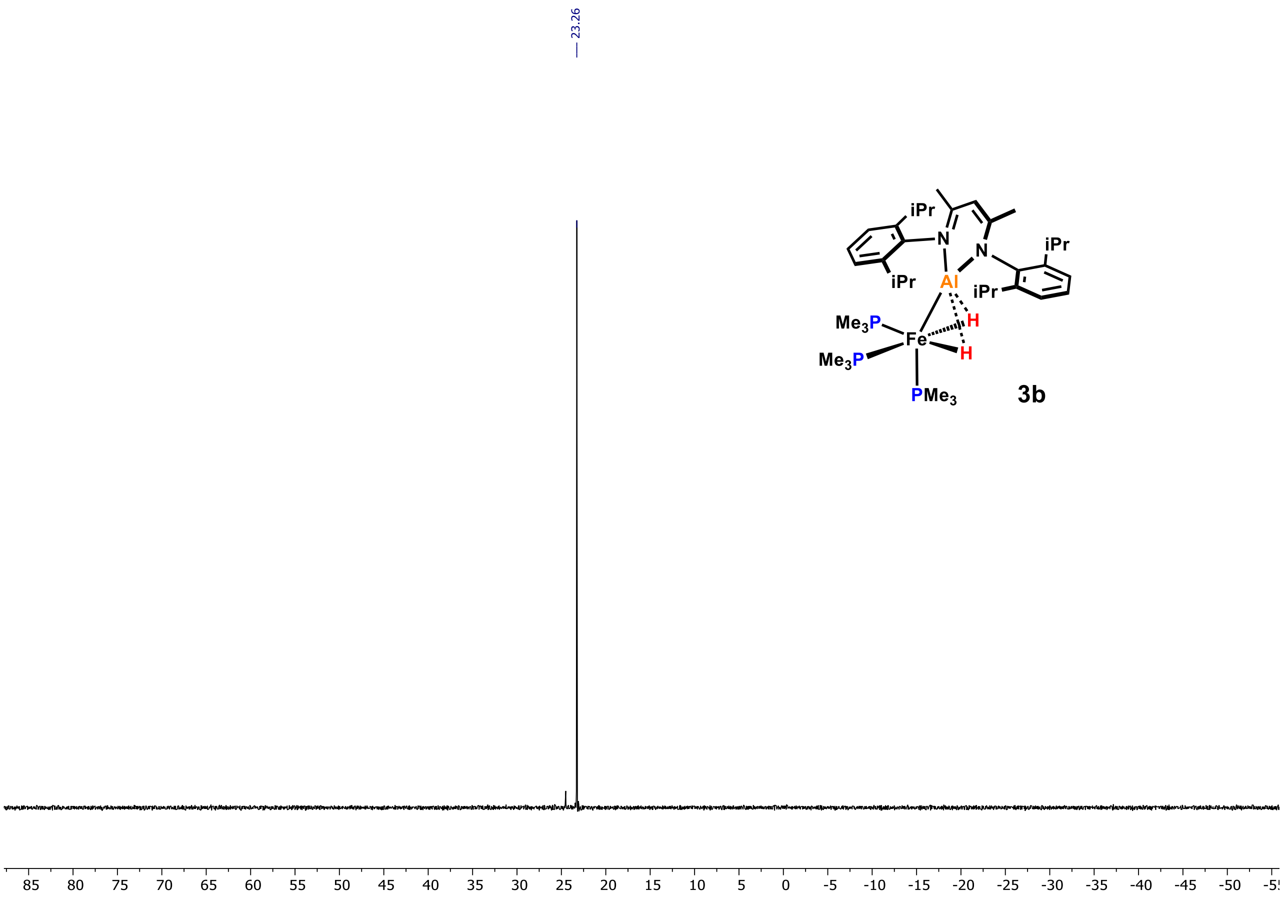

Figure S18. $^{13}C\{^{1}H\}$ NMR of **3b** (101 MHz, C_6D_6, 298 K).

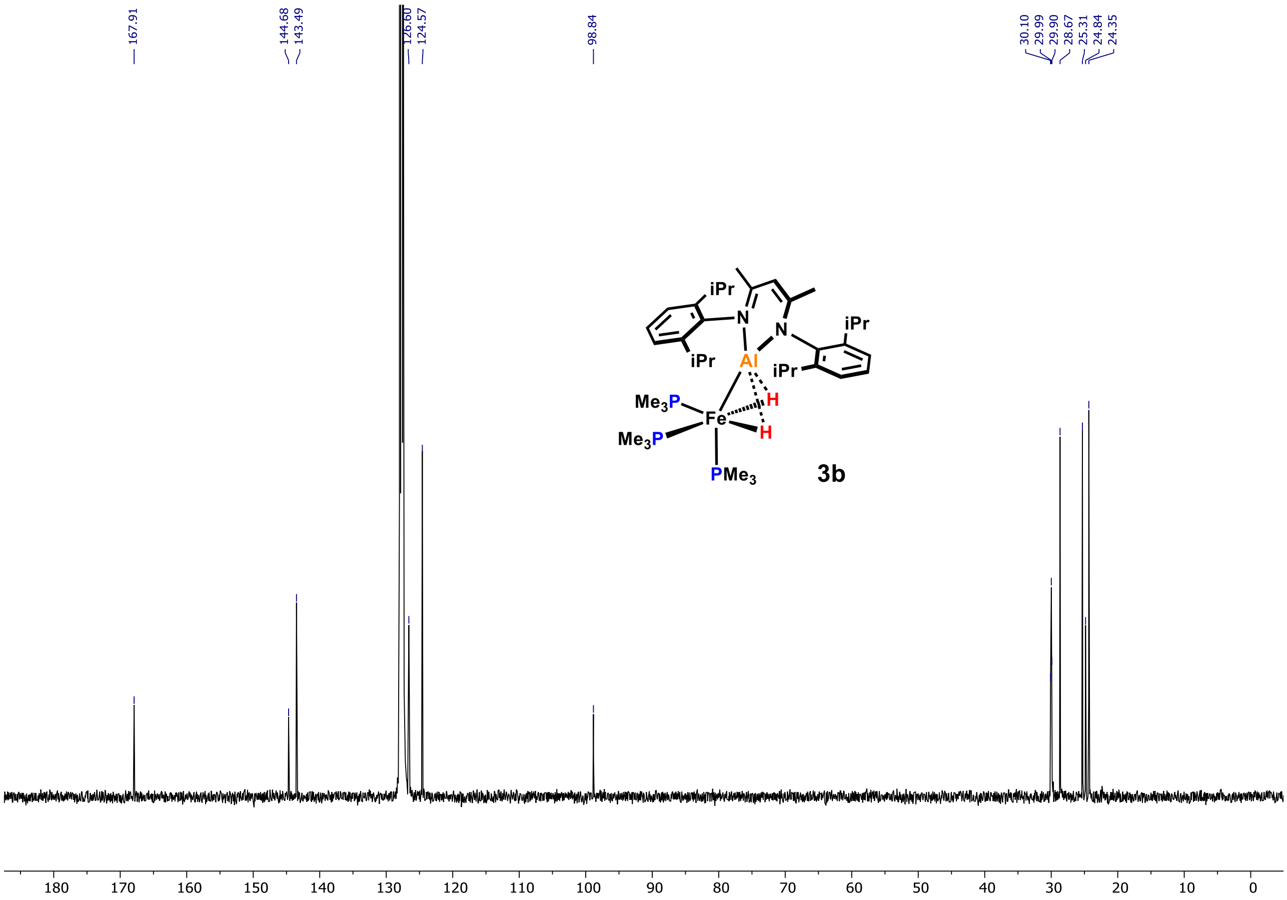

Figure S19. $^{13}C\{^{1}H\}$ NMR of **3b** (101 MHz, C_6D_6, 298 K).

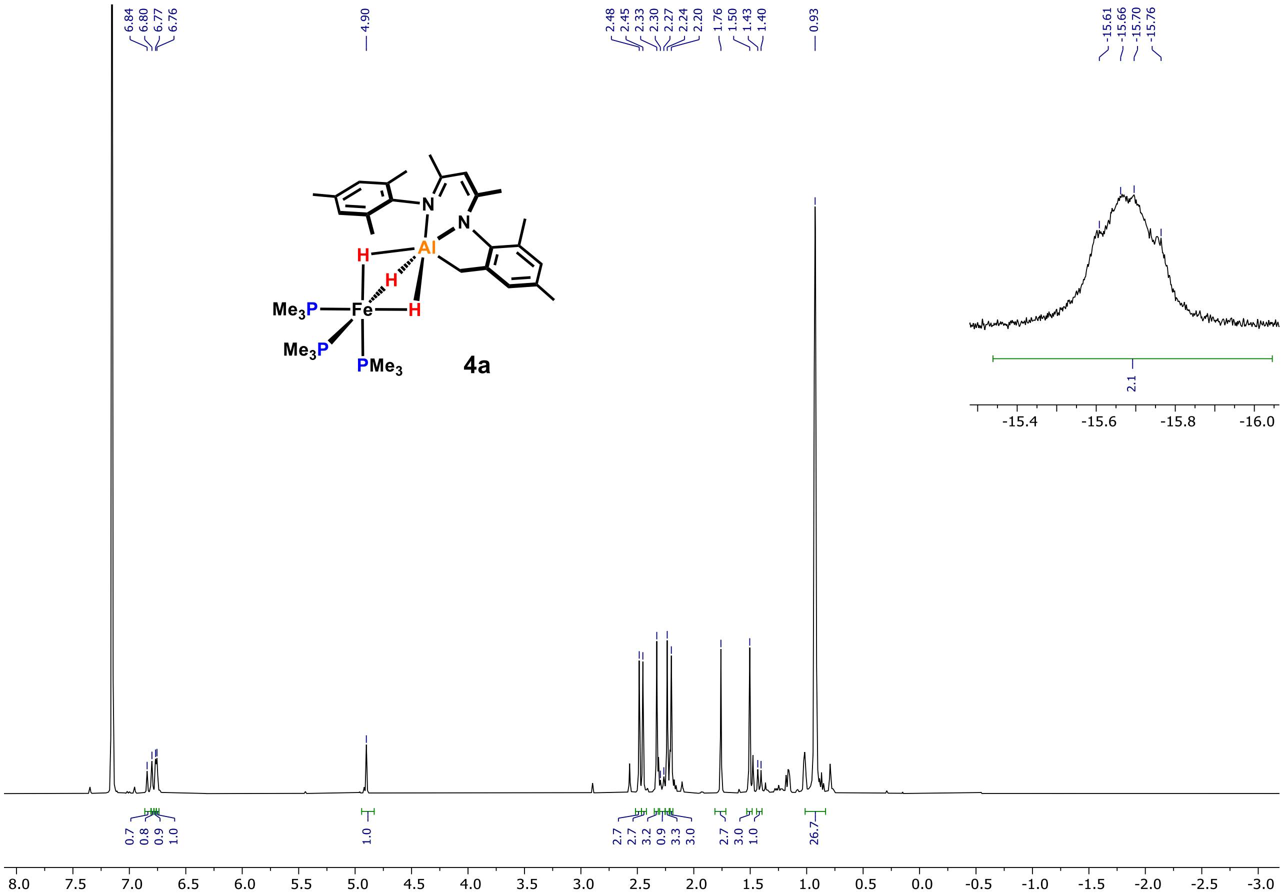

Figure S20. ^{1}H NMR of **4a** (400 MHz, C_6D_6, 298 K).

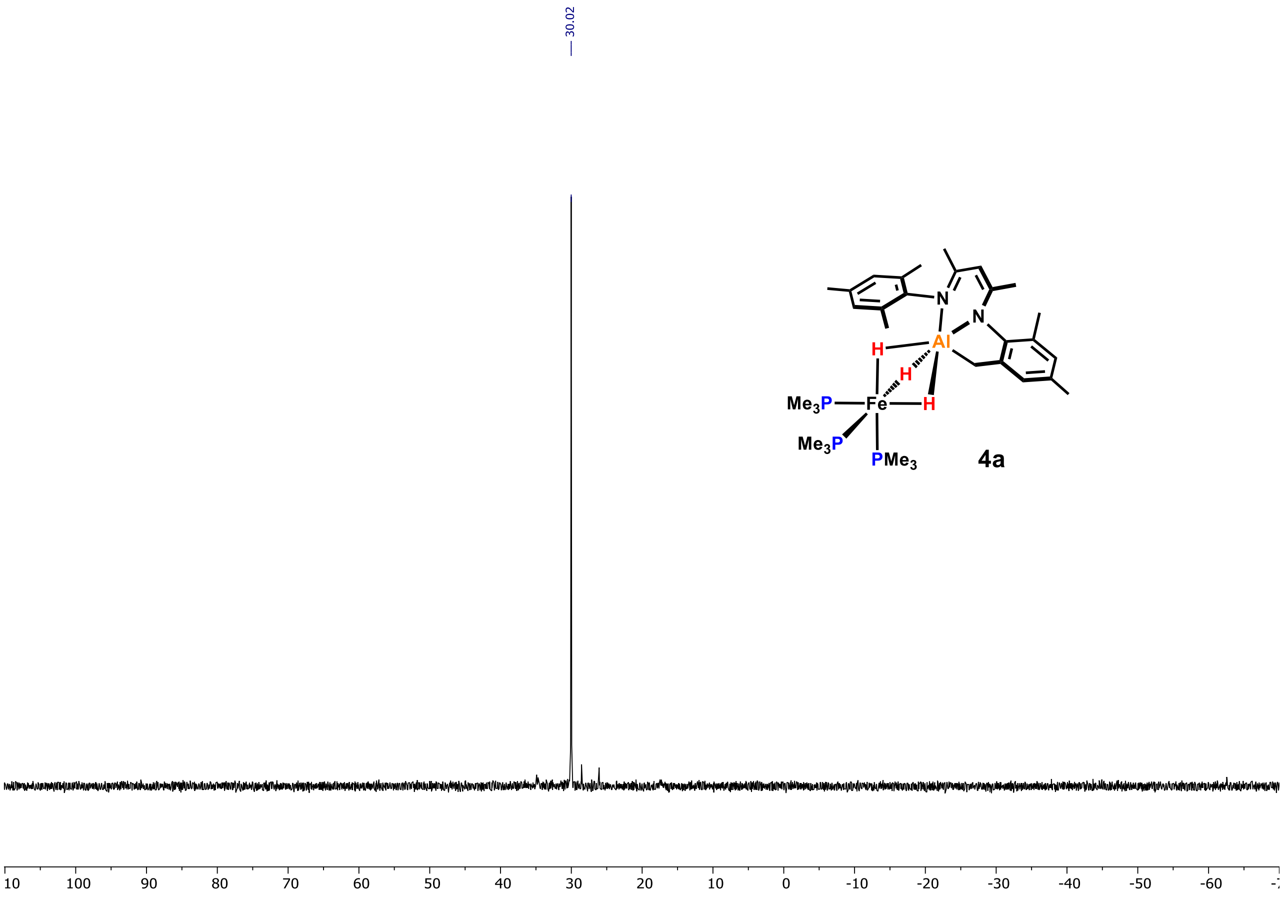

Figure S21. $^{31}P\{^{1}H\}$ NMR of **4a** (162 MHz, C_6D_6, 298 K).

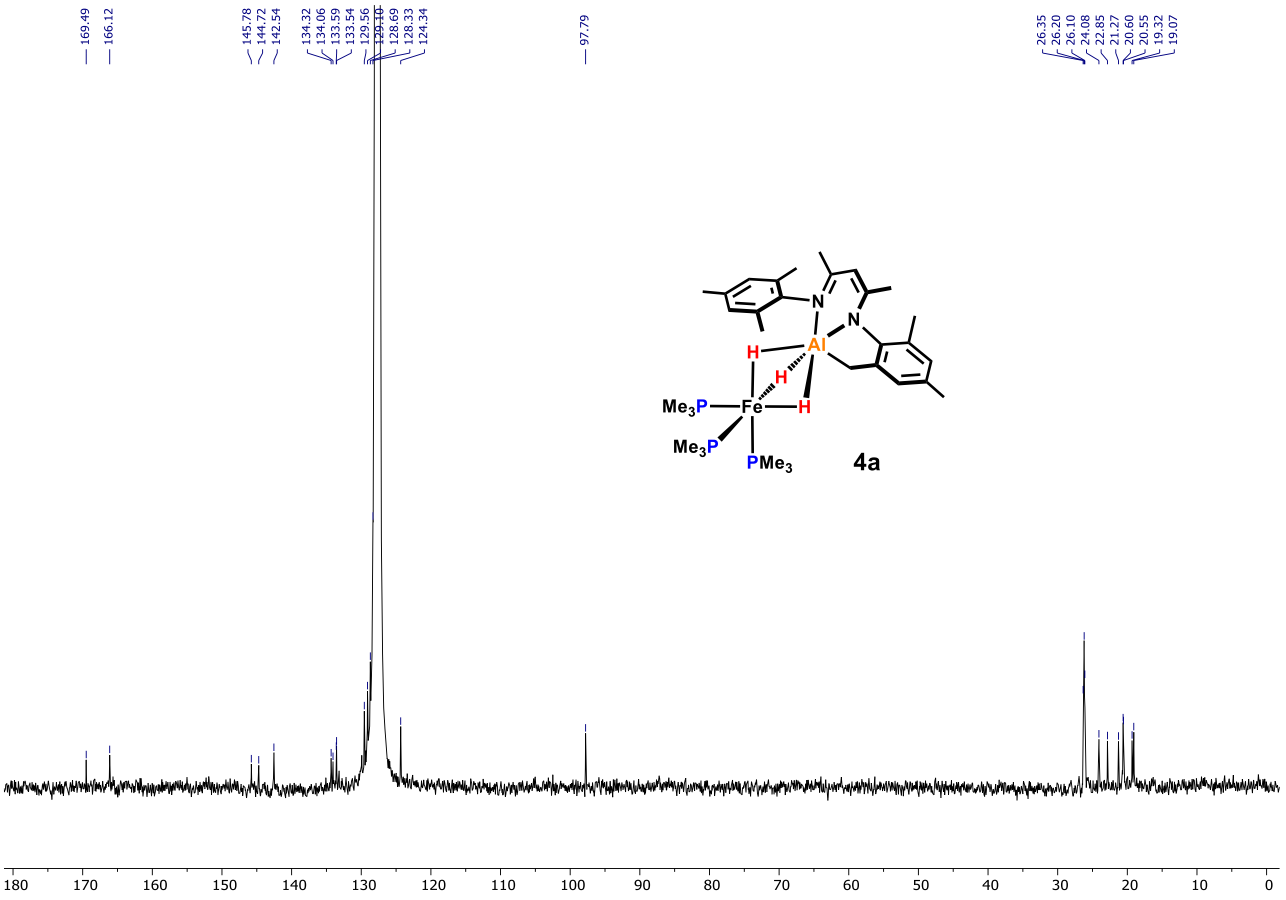

Figure S22. $^{13}C\{^{1}H\}$ NMR of **4a** (101 MHz, C_6D_6, 298 K).

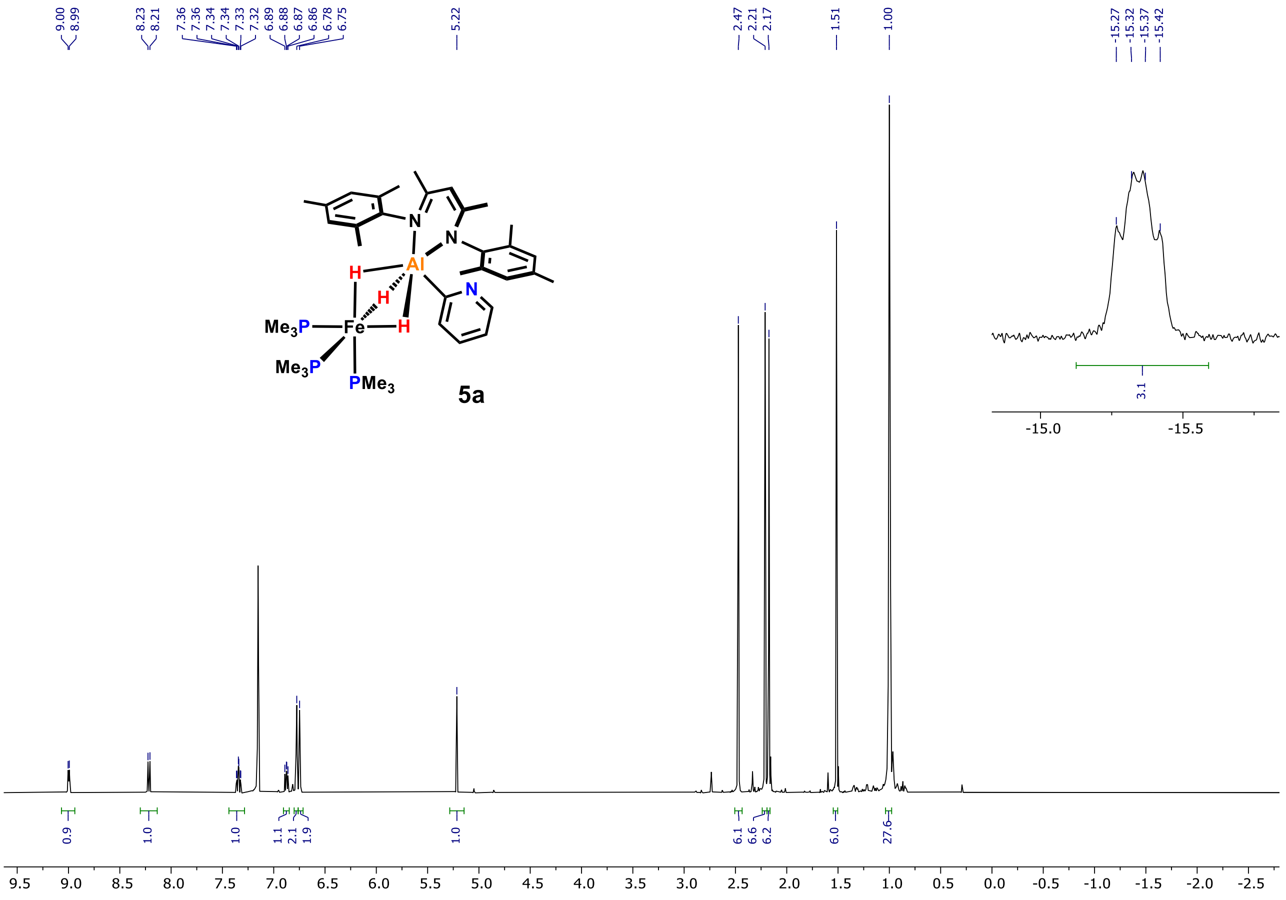

Figure S23. ^{1}H NMR of **5a** (400 MHz, C_6D_6, 298 K).

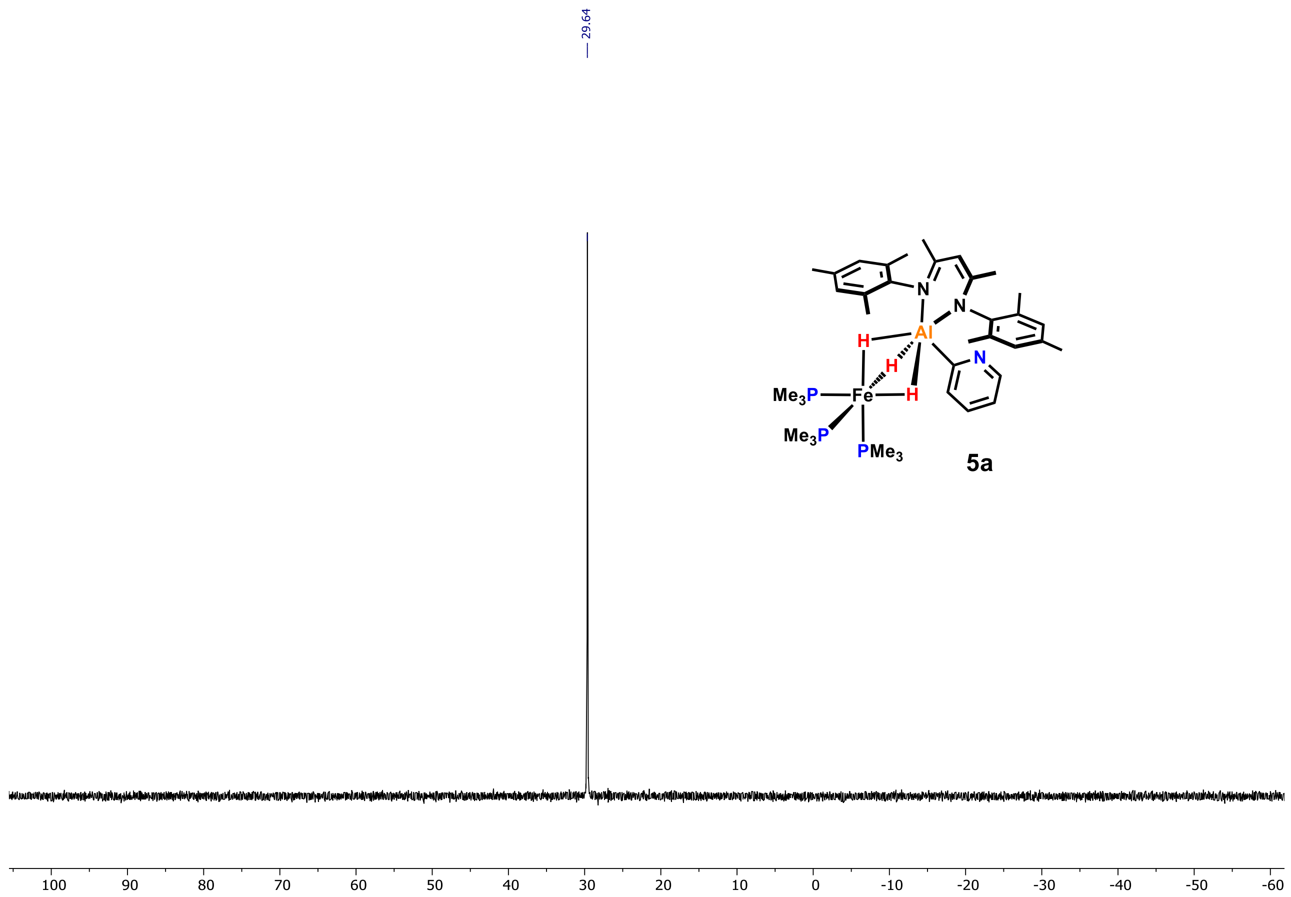

Figure S24. $^{31}P\{^1H\}$ NMR of **5a** (162 MHz, C_6D_6, 298 K).

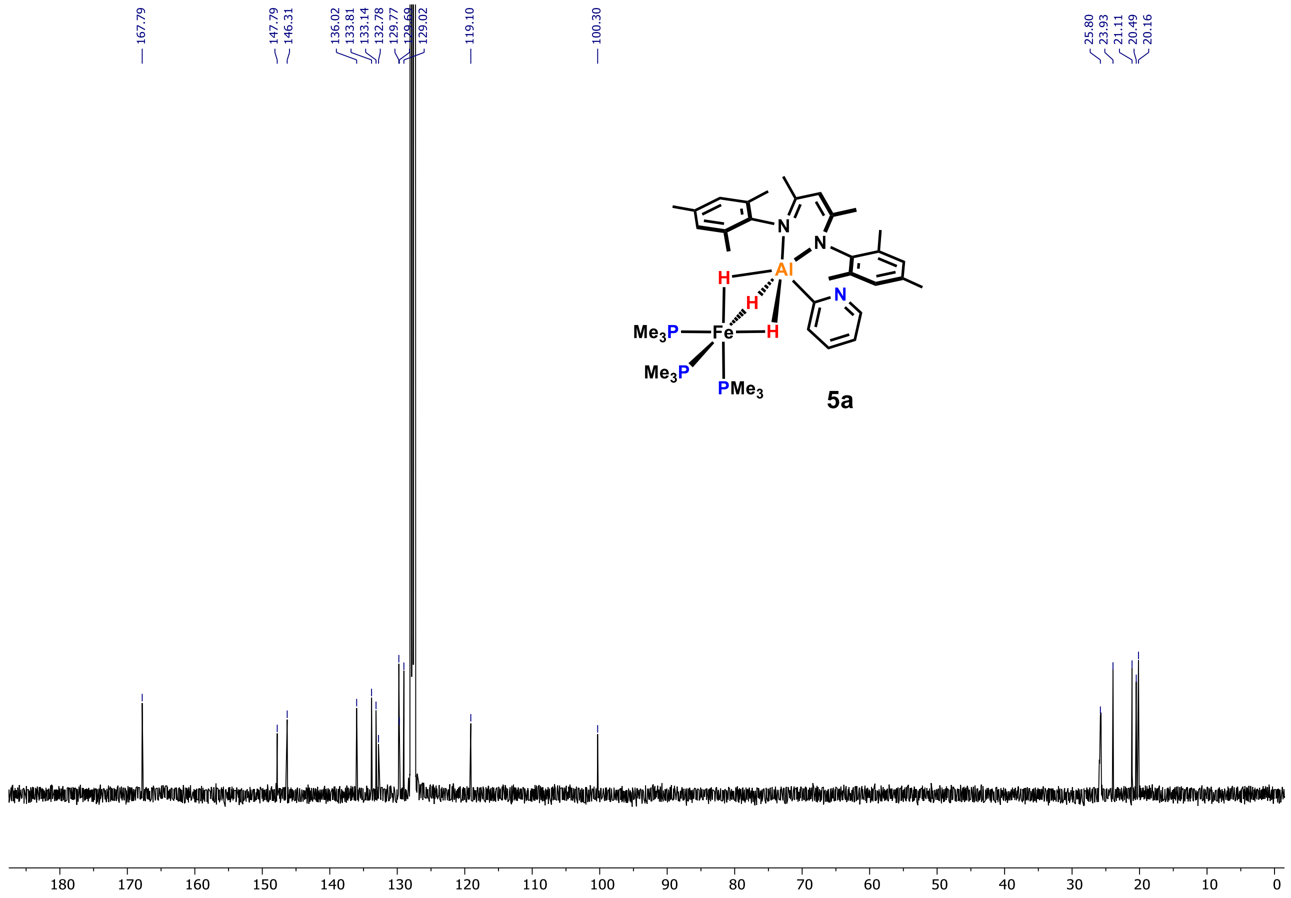

Figure S25. $^{13}C\{^1H\}$ NMR of **4a** (101 MHz, C_6D_6, 298 K).

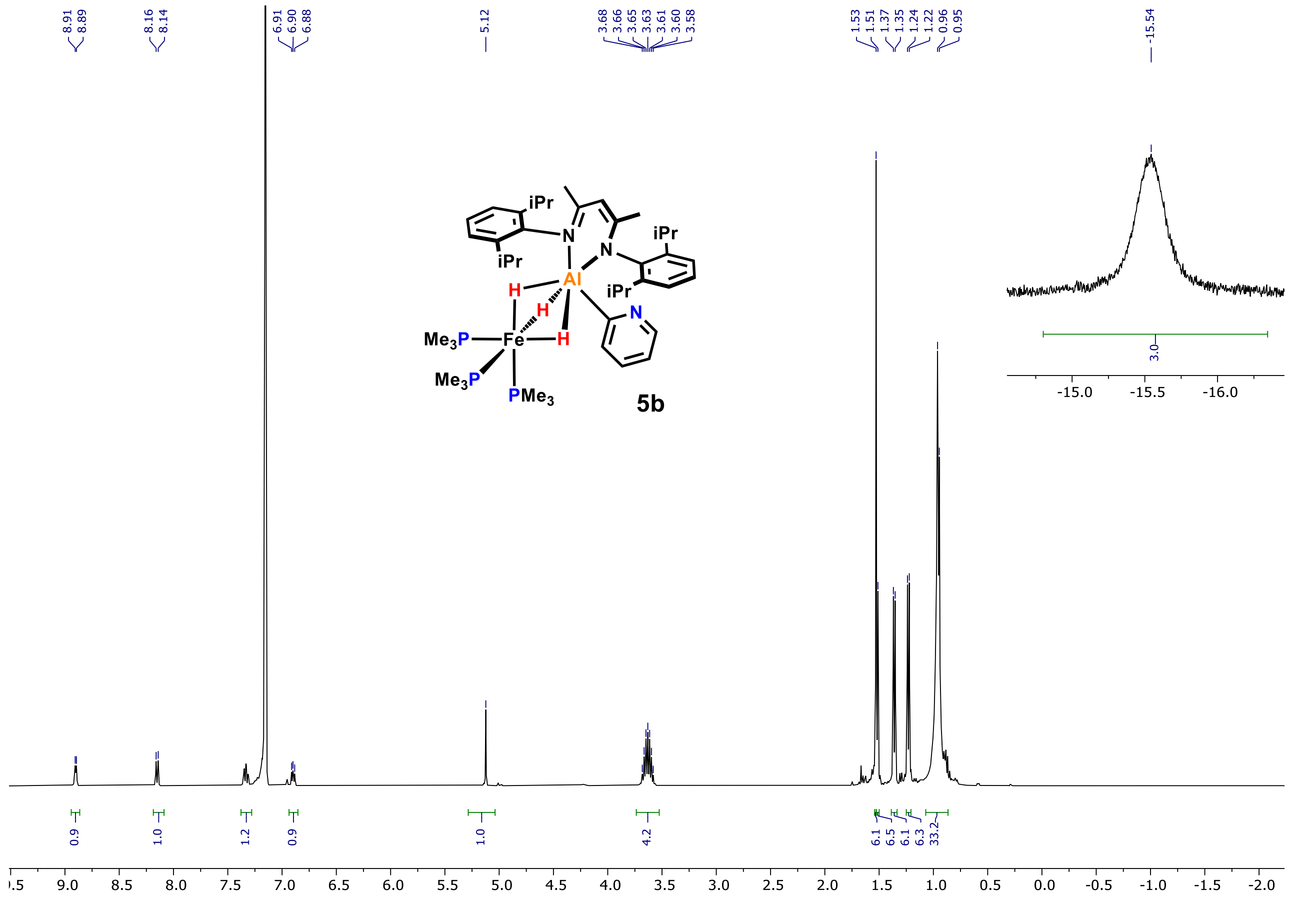

Figure S26. ^{1}H NMR of **5b** (400 MHz, C_6D_6, 298 K).

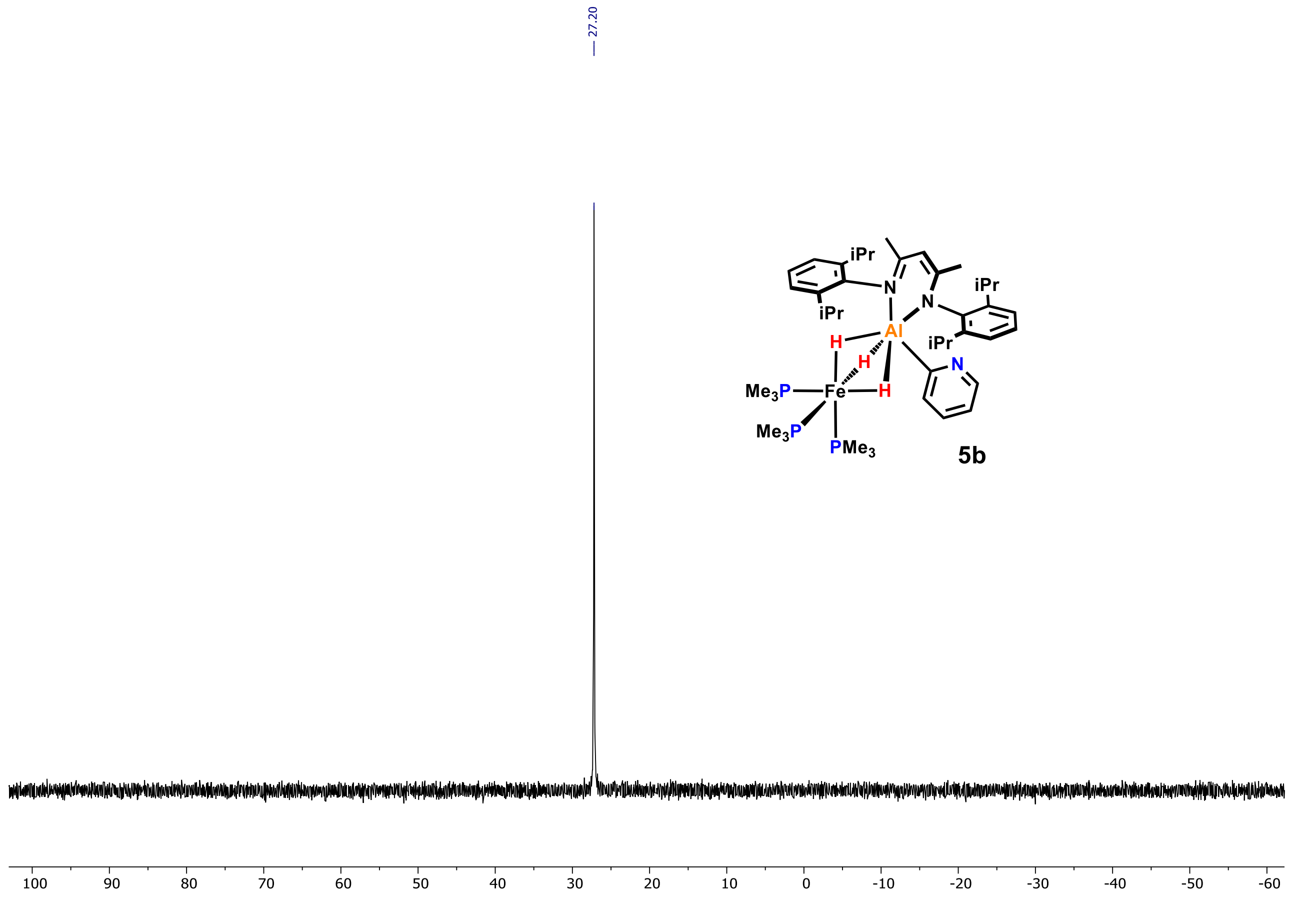

Figure S27. $^{31}P\{^{1}H\}$ NMR of **5b** (162 MHz, C_6D_6, 298 K).

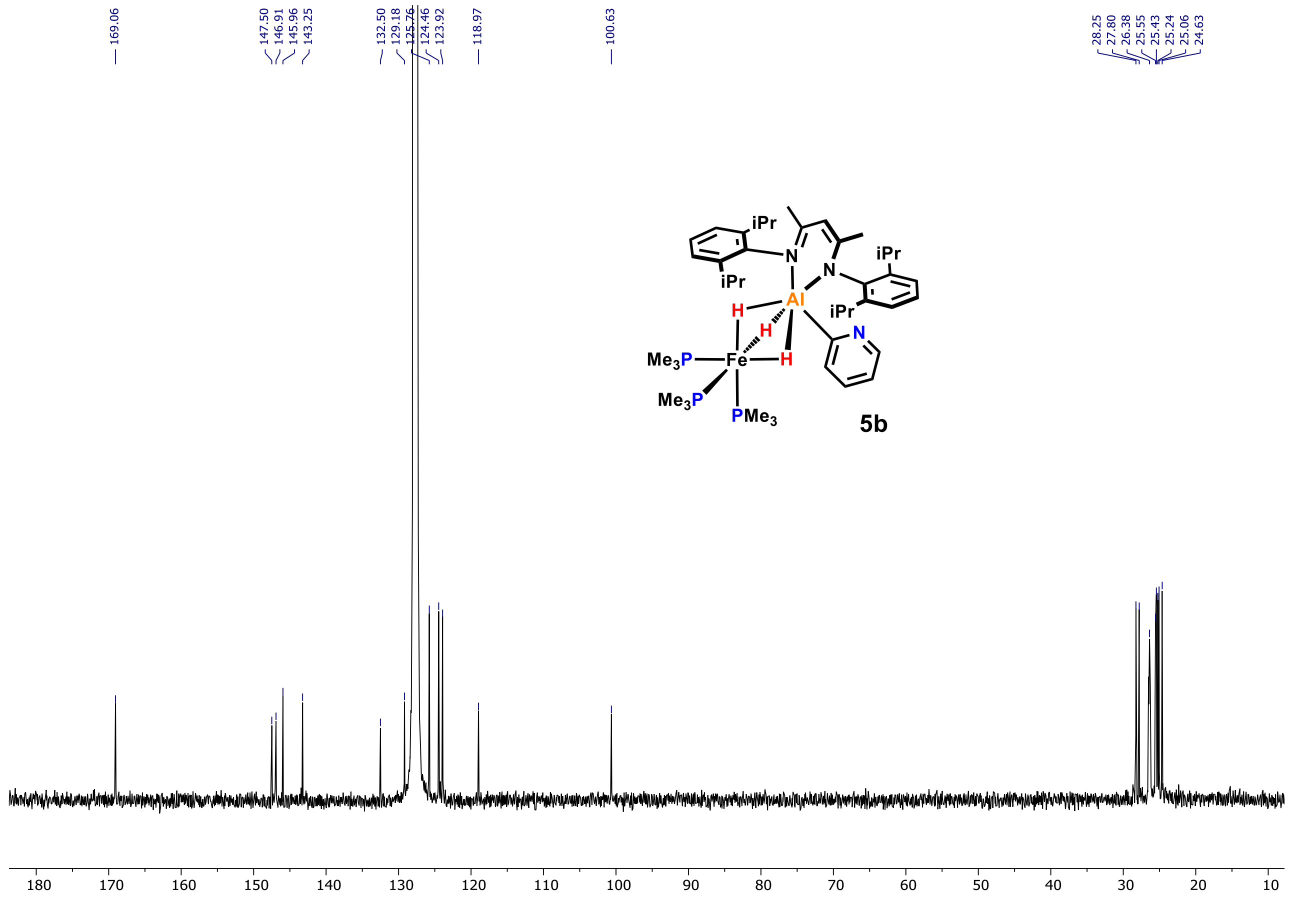

Figure S28. $^{13}C\{^1H\}$ NMR of **5b** (101 MHz, C_6D_6, 298 K).

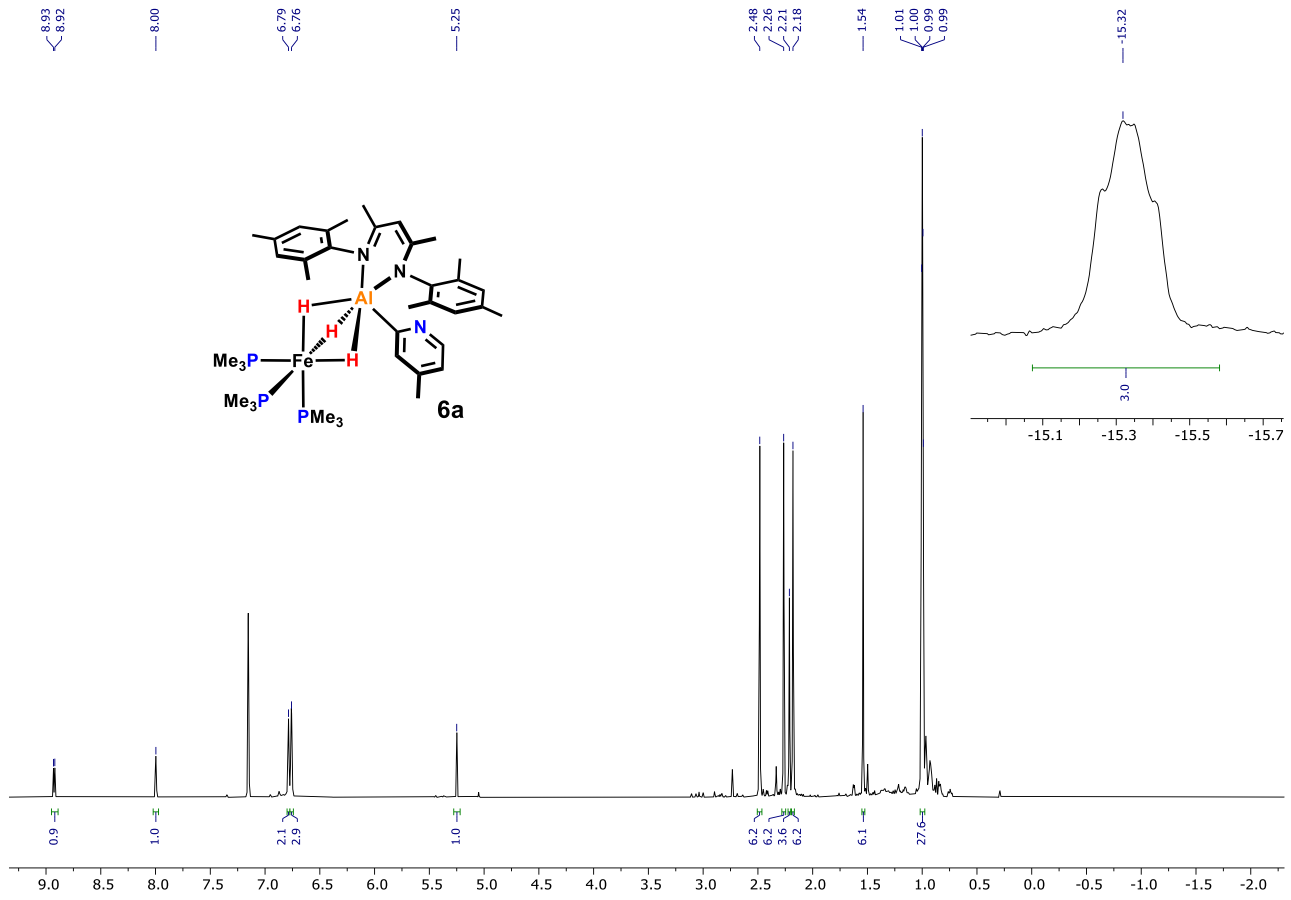

Figure S29. ^1H NMR of **6a** (400 MHz, C_6D_6, 298 K).

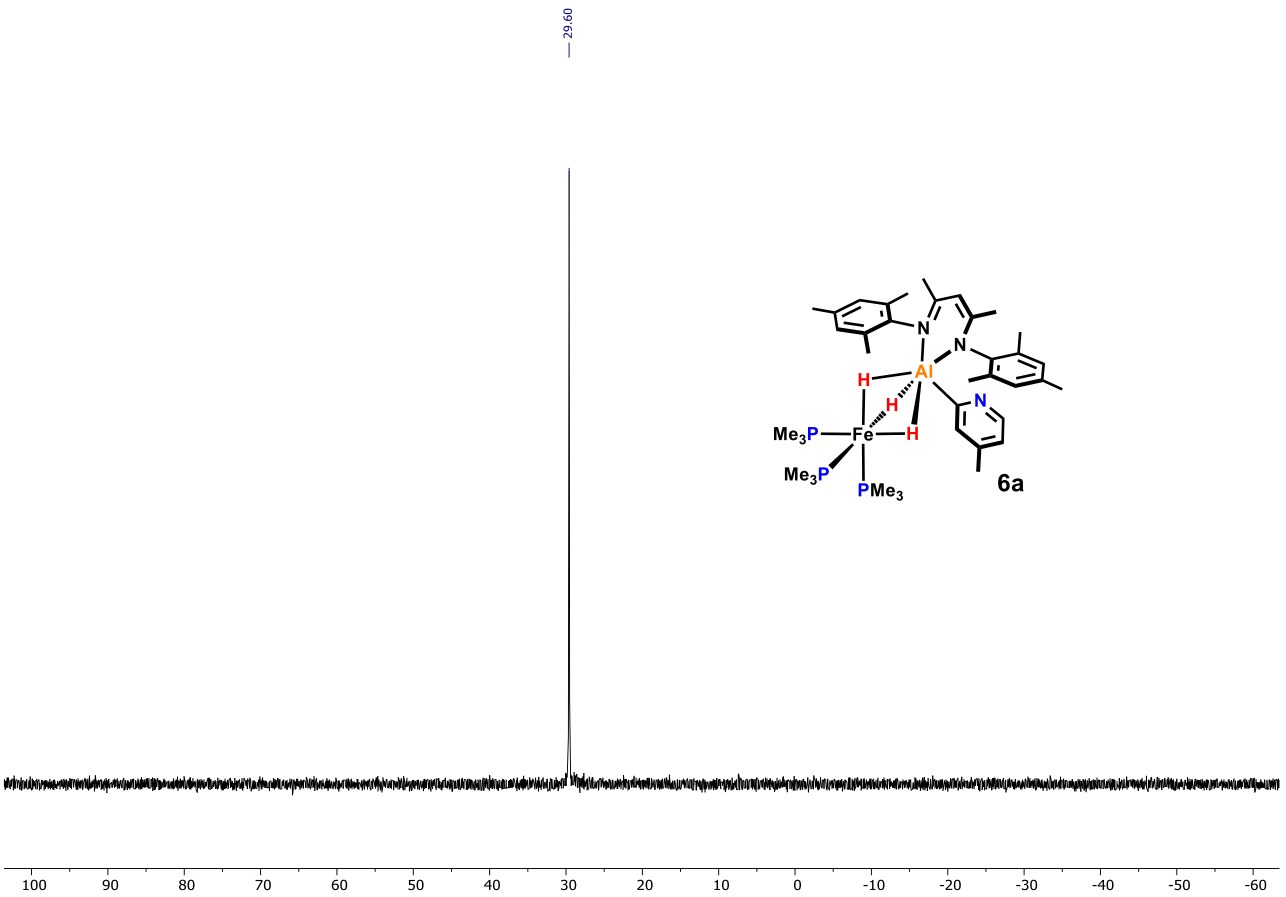

Figure S30. $^{31}P\{^{1}H\}$ NMR of **6a** (162 MHz, C_6D_6, 298 K).

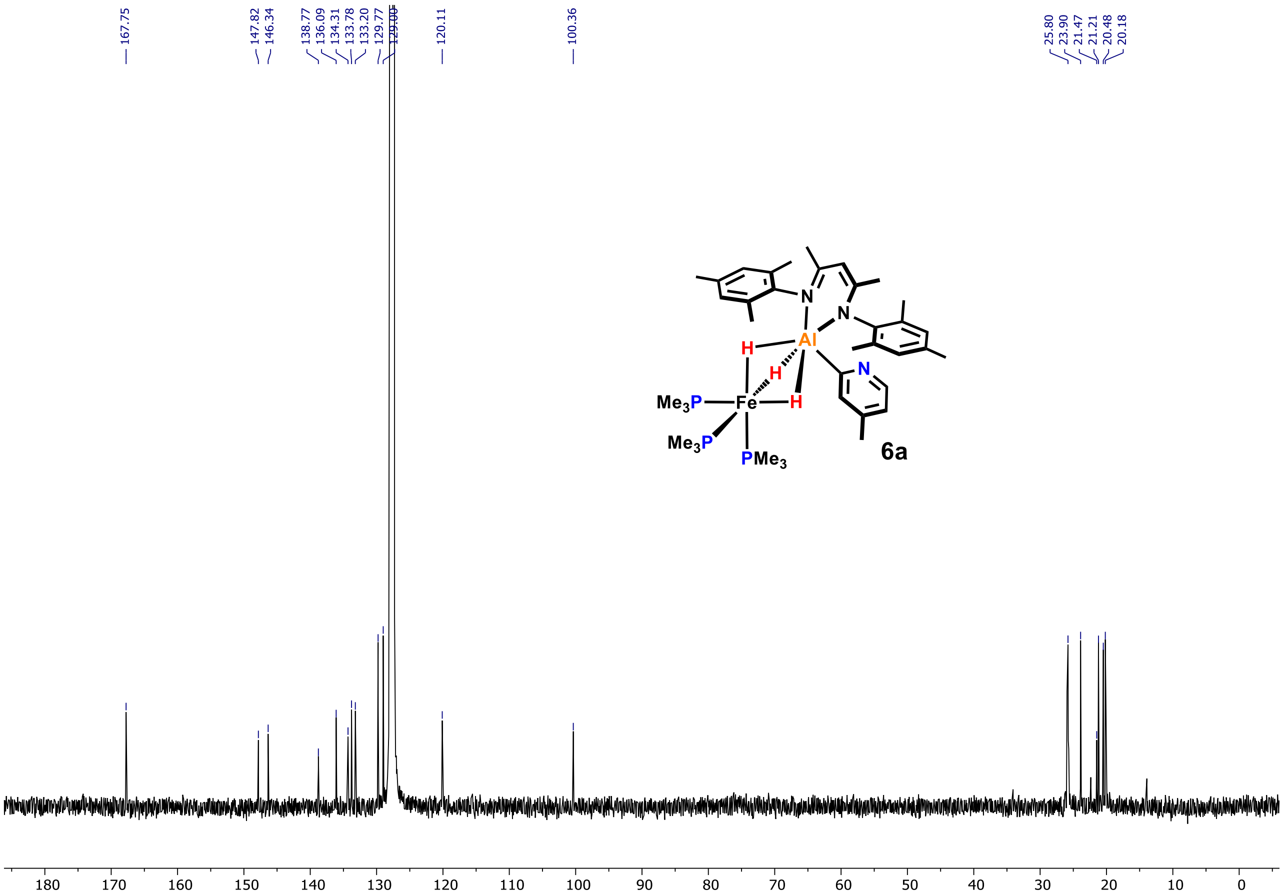

Figure S31. $^{13}C\{^{1}H\}$ NMR of **6a** (101 MHz, C_6D_6, 298 K).

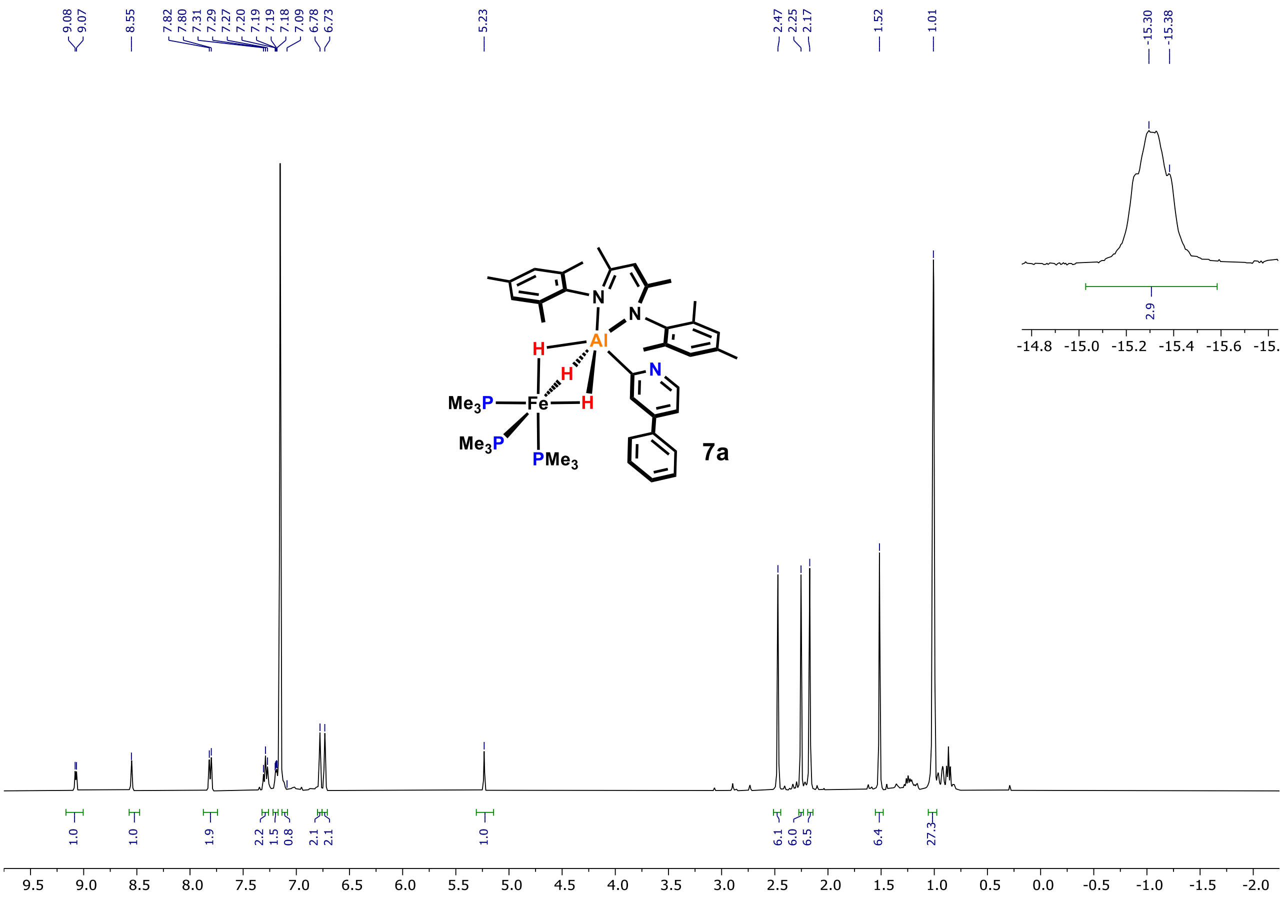

Figure S32. ^{1}H NMR of **7a** (400 MHz, C_6D_6, 298 K).

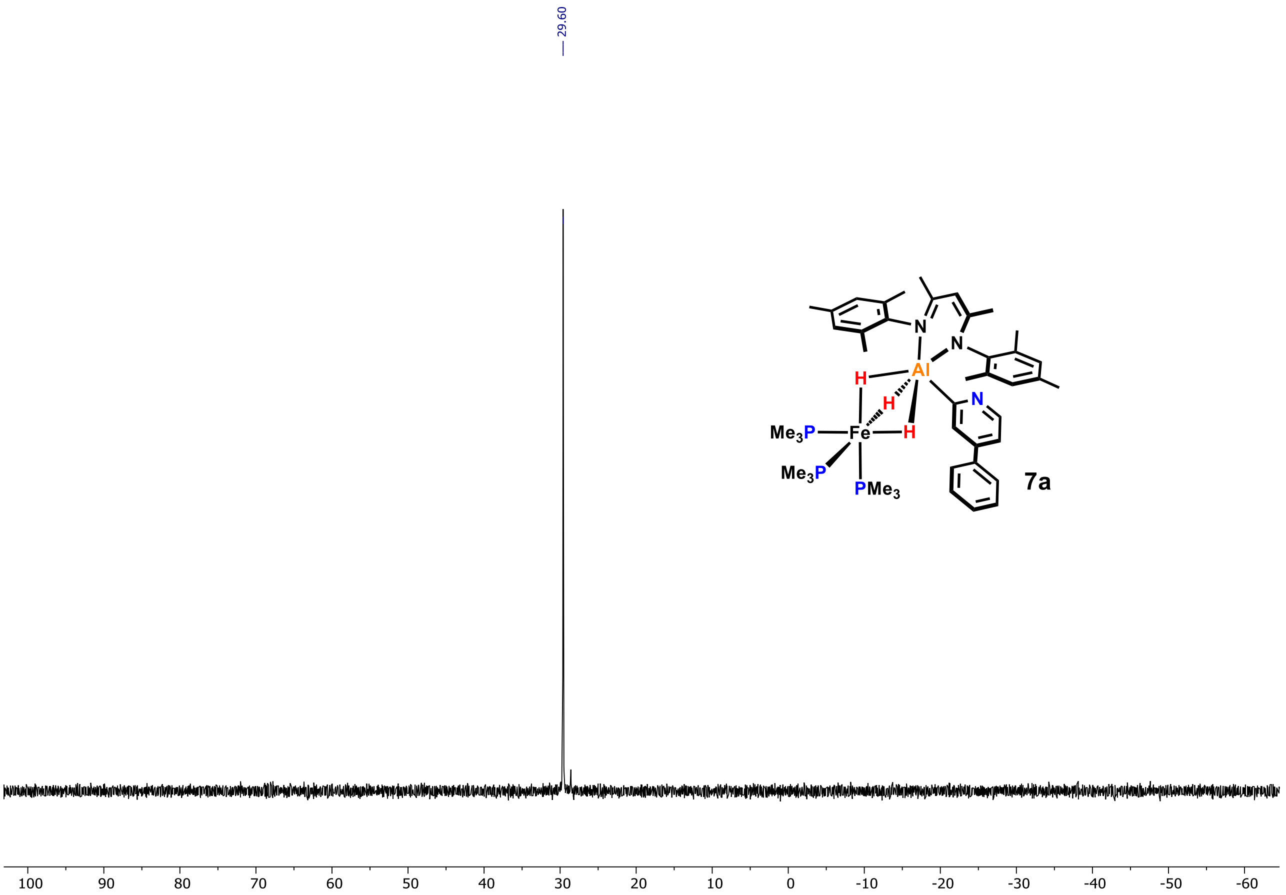

Figure S33. $^{31}P\{^{1}H\}$ NMR of **7a** (162 MHz, C_6D_6, 298 K).

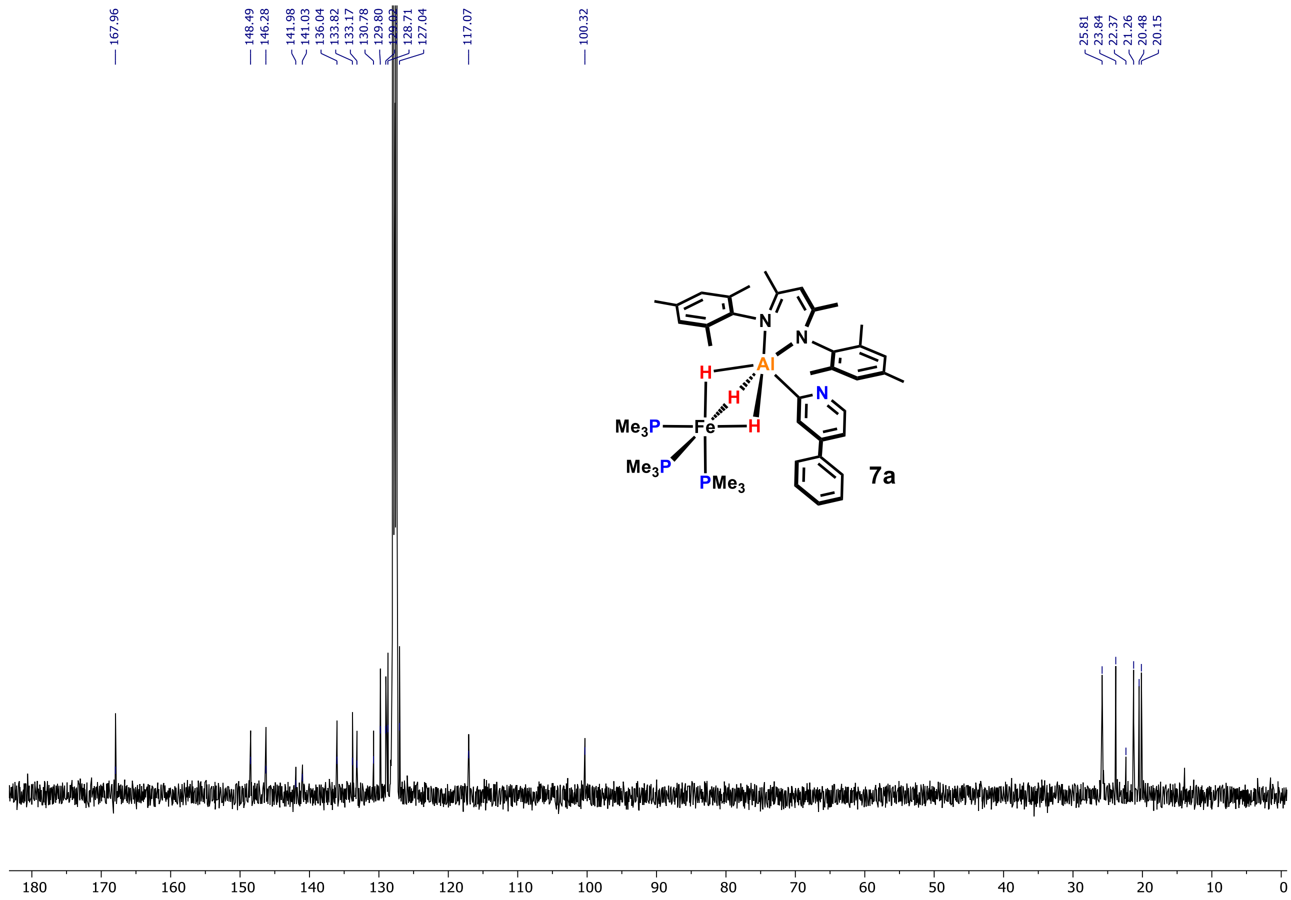

Figure S34. $^{13}C\{^{1}H\}$ NMR of **7a** (101 MHz, C_6D_6, 298 K).

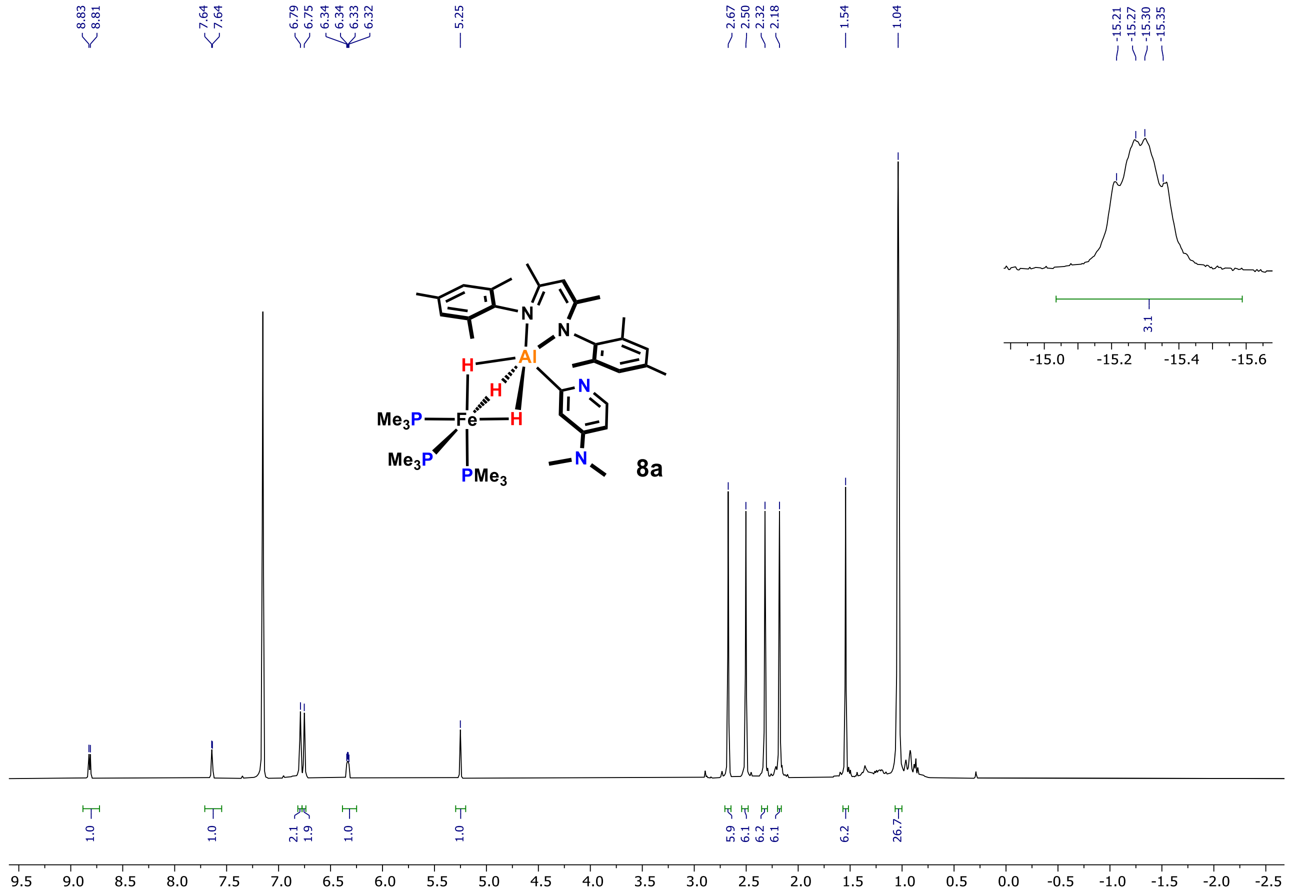

Figure S35. ^{1}H NMR of **8a** (400 MHz, C_6D_6, 298 K).

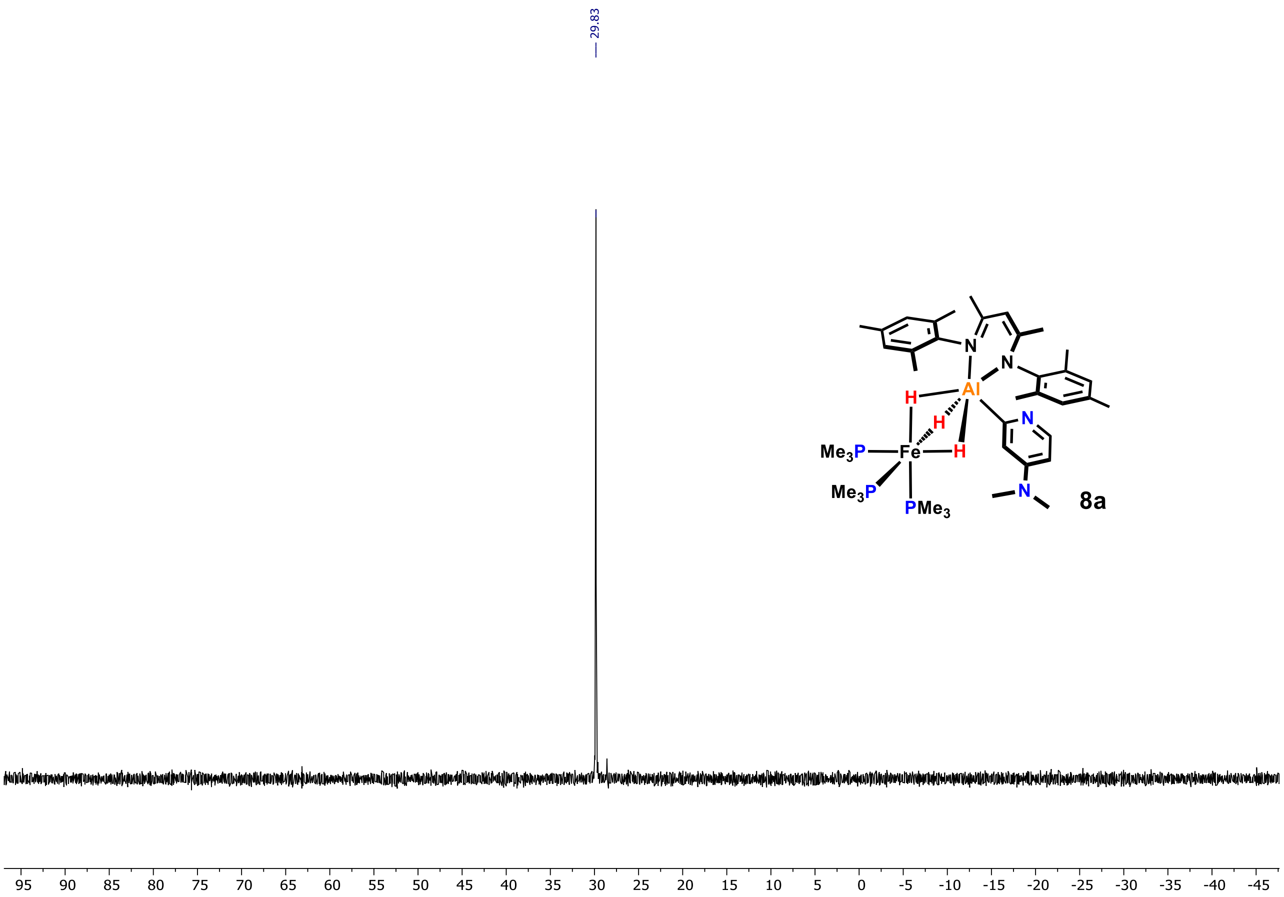

Figure S36. $^{31}P\{^{1}H\}$ NMR of **8a** (162 MHz, C_6D_6, 298 K).

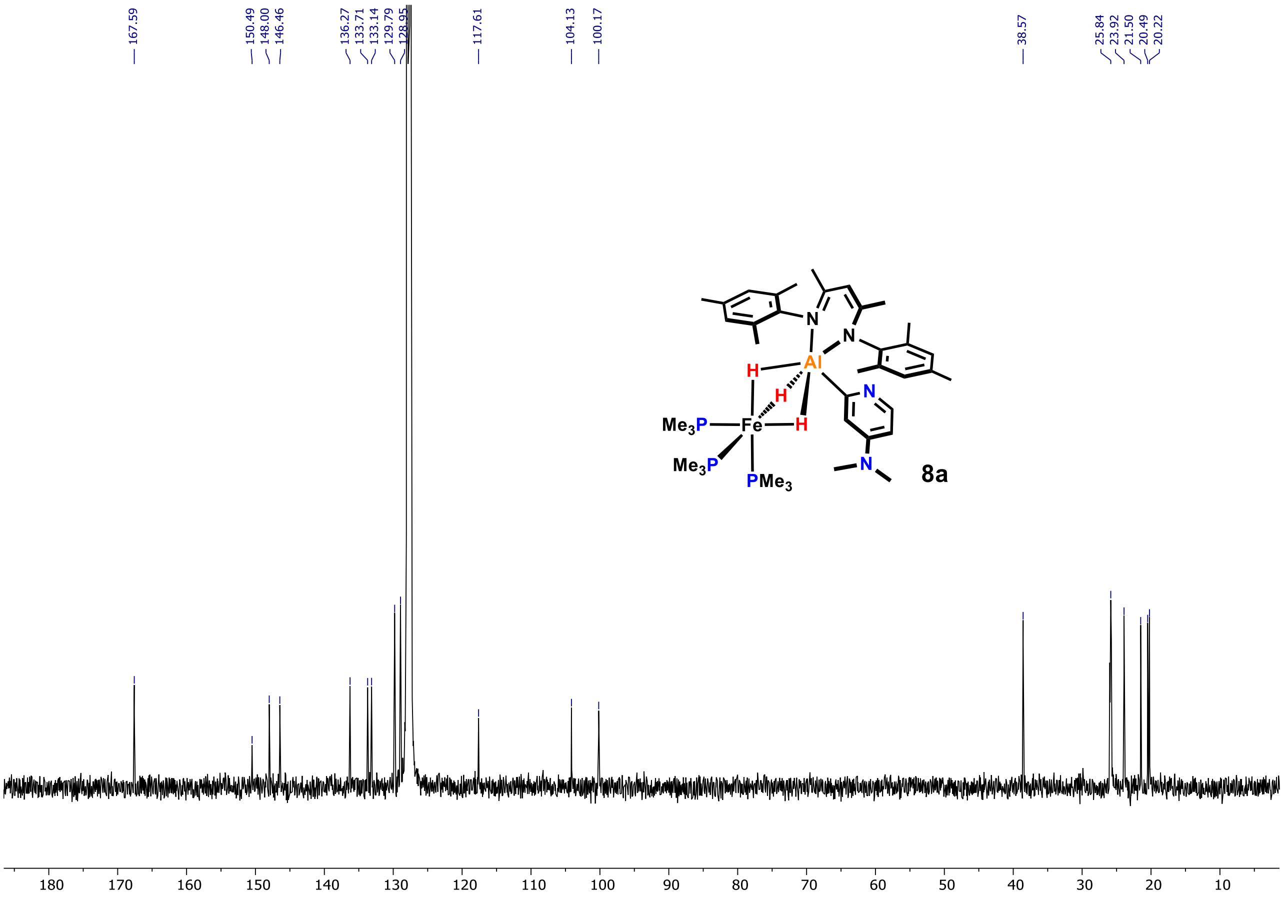

Figure S37. $^{13}C\{^{1}H\}$ NMR of **8a** (101 MHz, C_6D_6, 298 K).

7. Selected IR Spectra

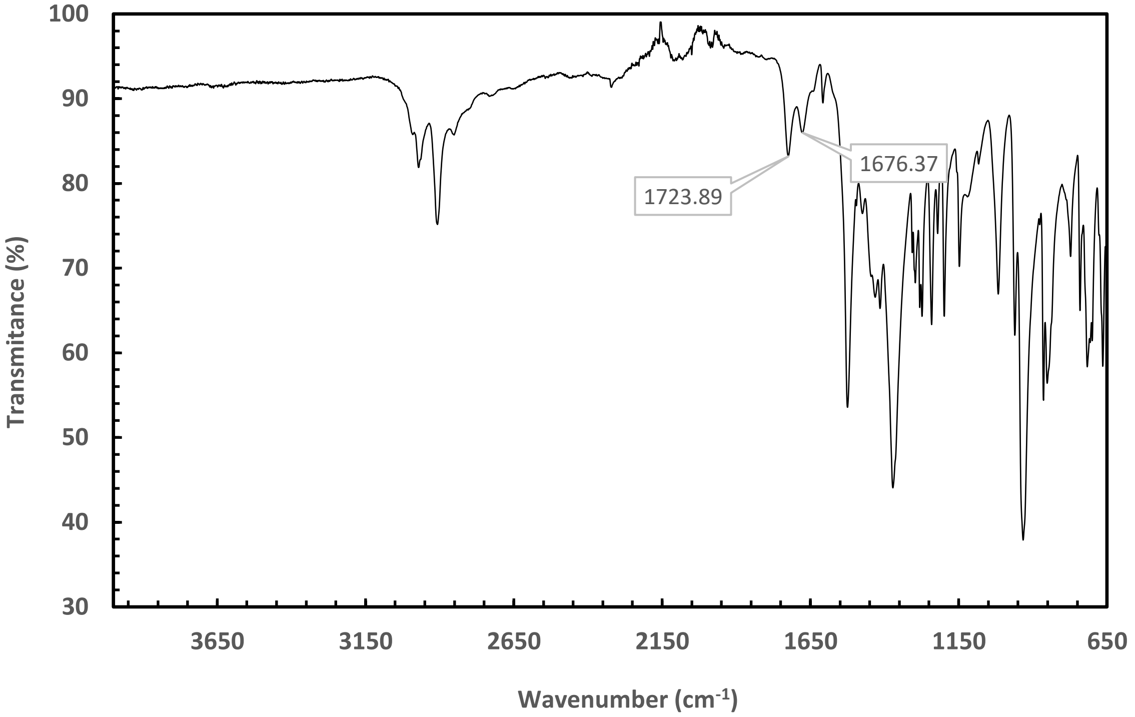

Figure S38. ATR-IR spectrum of complex **2a**. The annotated bands refer to the M–H stretching frequencies.

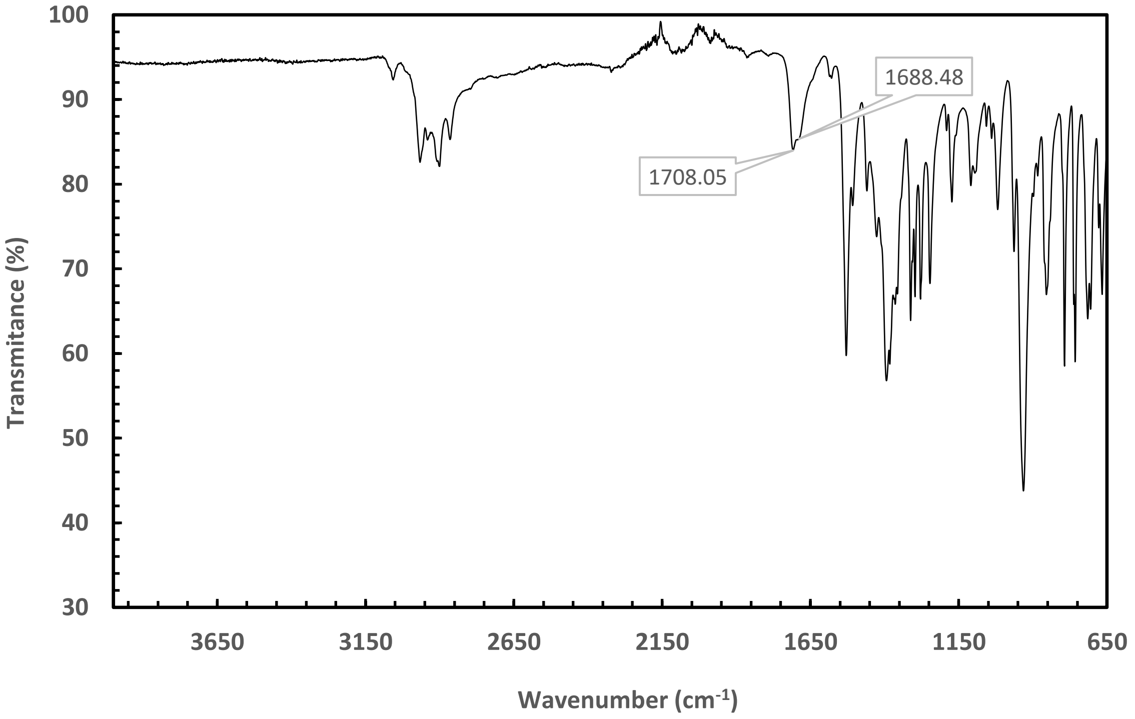

Figure S39. ATR-IR spectrum of complex **2b**. The annotated bands refer to the M–H stretching frequencies.

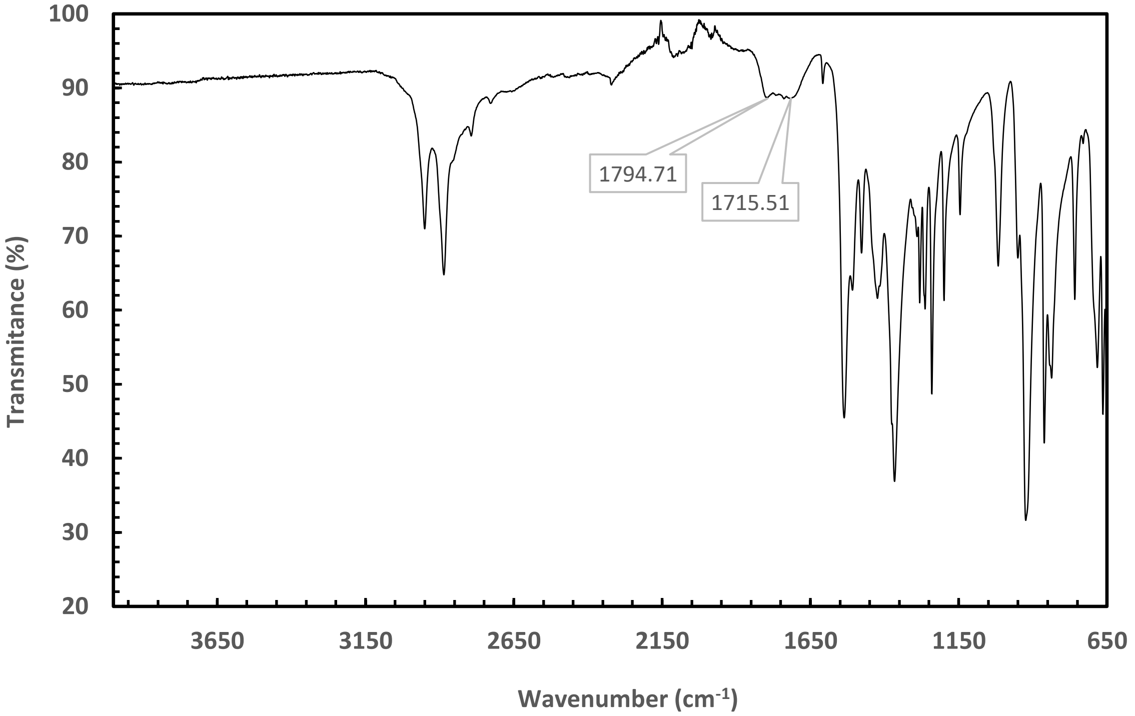

Figure S40. ATR-IR spectrum of complex **3a**. The annotated bands refer to the M–H stretching frequencies.

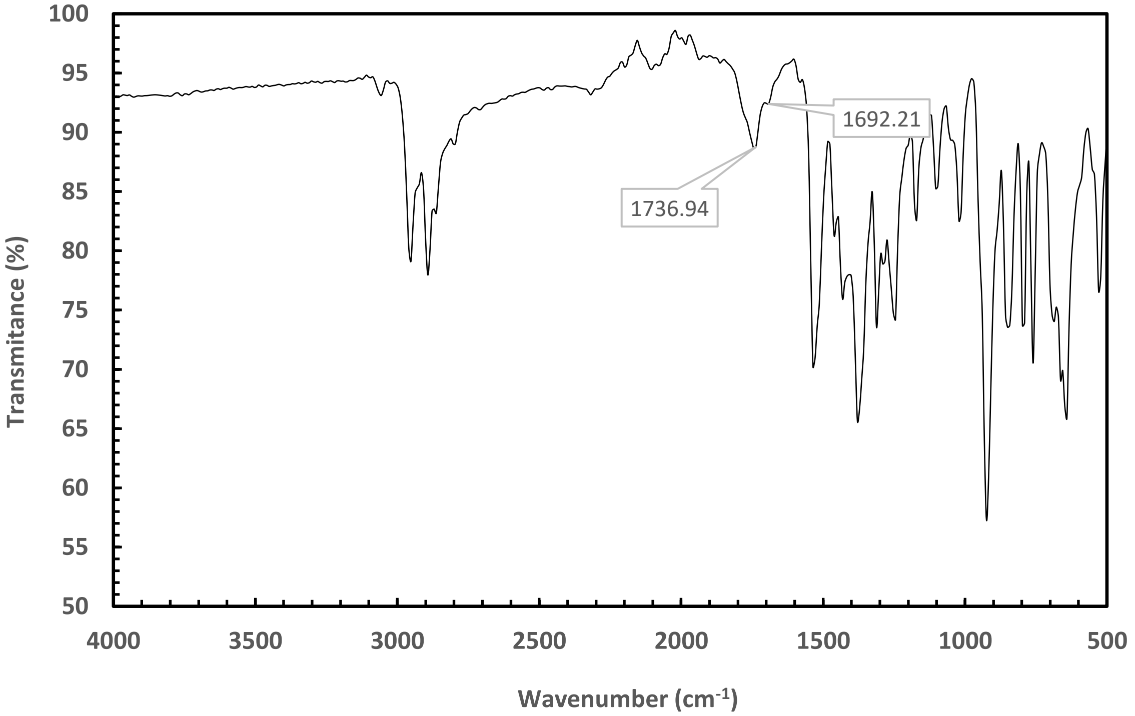

Figure S41. ATR-IR spectrum of complex **3b**. The annotated bands refer to the M–H stretching frequencies.

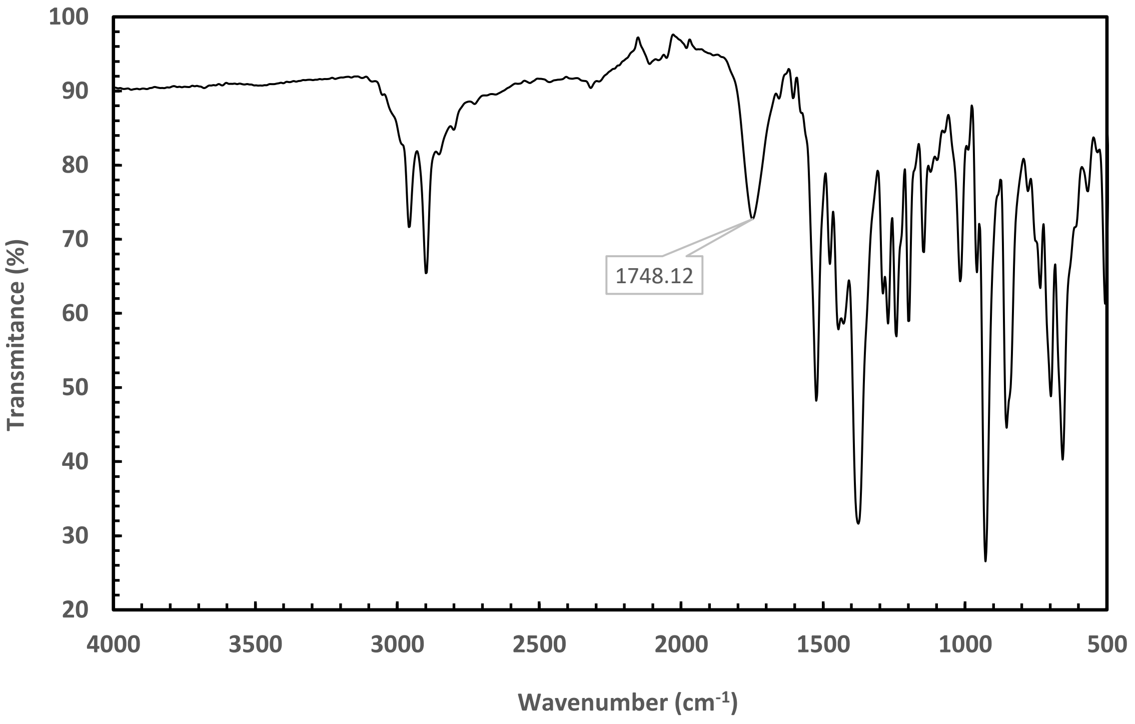

Figure S42. ATR-IR spectrum of complex **5a**. The annotated bands refer to the M–H stretching frequencies.

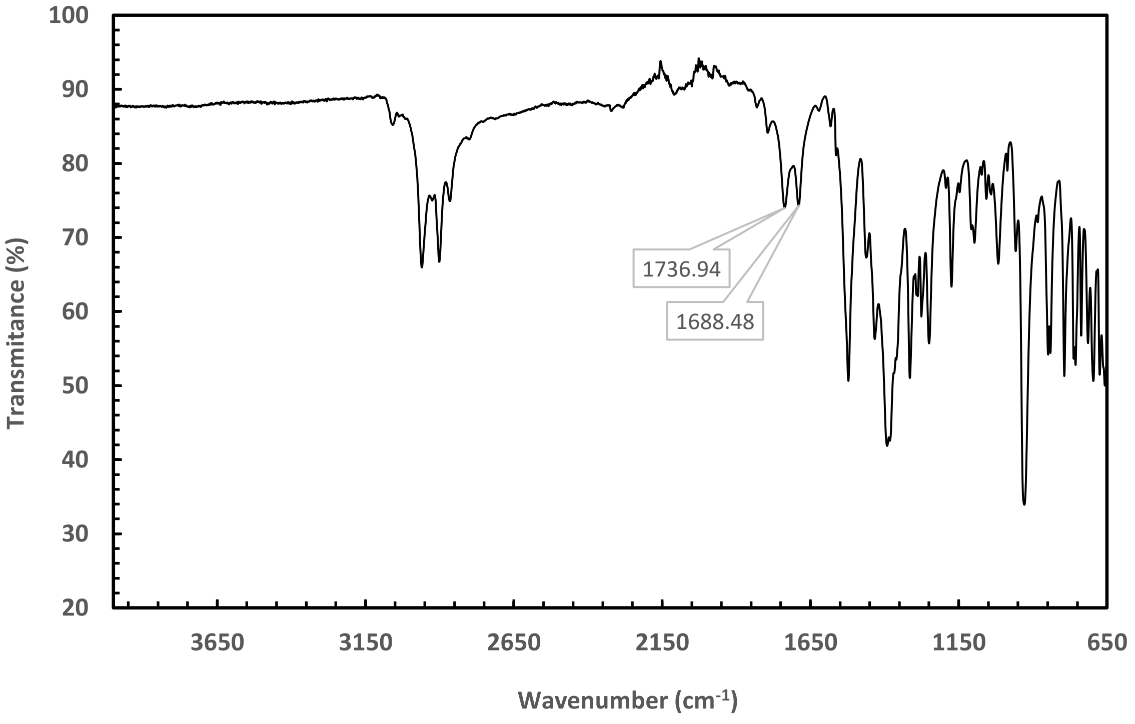

Figure S43. ATR-IR spectrum of complex **5b**. The annotated bands refer to the M–H stretching frequencies.

8. References

[1] Yow, S.; Gates, S. J.; White, A. J. P.; Crimmin, M. R. Zirconocene Dichloride Catalyzed Hydrodefluorination of C(sp^2)–F Bonds. *Angew. Chemie Int. Ed.* **2012**, *51* (50), 12559–12563.

[2] SHELXTL v5.1, Bruker AXS, Madison, WI, **1998**.

[3] SHELX-2013, G.M. Sheldrick, Acta Cryst., **2015**, C71, 3-8.

[4] Frisch, M. J.; Trucks, G. W.; Schlegel, H. B.; Scuseria, G. E.; Robb, M. A.; Cheeseman, J. R.; Scalmani, G.; Barone, V.; Mennucci, B.; Petersson, G. A.; Nakatsuji, H.; Caricato, M.; Li, X.; Hratchian, H. P.; Izmaylov, A. F.; Bloino, J.; Zheng, G.; Sonnenberg, J. L.; Hada, M.; Ehara, M.; Toyota, K.; Fukuda, R.; Hasegawa, J.; Ishida, M.; Nakajima, T.; Honda, Y.; Kitao, O.; Nakai, H.; Vreven, T.; Montgomery, J. A., Jr.; Peralta, J. E.; Ogliaro, F.; Bearpark, M.; Heyd, J. J.; Brothers, E.; Kudin, K. N.; Staroverov, V. N.; Kobayashi, R.; Normand, J.; Raghavachari, K.; Rendell, A.; Burant, J. C.; Iyengar, S. S.; Tomasi, J.; Cossi, M.; Rega, N.; Millam, J. M.; Klene, M.; Knox, J. E.; Cross, J. B.; Bakken, V.; Adamo, C.; Jaramillo, J.; Gomperts, R.; Stratmann, R. E.; Yazyev, O.; Austin, A. J.; Cammi, R.; Pomelli, C.; Ochterski, J. W.; Martin, R. L.; Morokuma, K.; Zakrzewski, V. G.; Voth, G. A.; Salvador, P.; Dannenberg, J. J.; Dapprich, S.; Daniels, A. D.; Farkas, Ö.; Foresman, J. B.; Ortiz, J. V.; Cioslowski, J.; Fox, D. J. Gaussian 09, Revision D.01; Gaussian, Inc., Wallingford, CT, **2009**.

[5] NBO 6.0. Glendening, E. D.; Badenhoop, J. K.; Reed, A. E.; Carpenter, J. E.; Bohmann, J. A.; Morales, C. M.; Landis, C. R.; Weinhold, F. Theoretical Chemistry Institute, University of Wisconsin, Madison (2013).

[6] AIMAll (Version 13.10.19), Todd A. Keith, TK Gristmill Software, Overland Park KS, USA, 2013 (aim.tkgristmill.com)

[7] Neese, F. Software Update: The ORCA Program System, Version 4.0. *WIREs Comput. Mol. Sci.* **2018**, *8* (1).

[8] Neese, F.; Wennmohs, F.; Becker, U.; Riplinger, C. The ORCA Quantum Chemistry Program Package. *J. Chem. Phys.* **2020**, *152* (22), 224108.

[9] Neese, F.; Wennmohs, F.; Hansen, A.; Becker, U. Efficient, Approximate and Parallel Hartree–Fock and Hybrid DFT Calculations. A 'Chain-of-Spheres' Algorithm for the Hartree–Fock Exchange. *Chem. Phys.* **2009**, *356* (1–3), 98–109.
